# Reversible male contraception by targeted inhibition of serine/threonine kinase 33

**DOI:** 10.1126/science.adl2688

**Published:** 2024-05-23

**Authors:** Angela F. Ku, Kiran L. Sharma, Hai Minh Ta, Courtney M. Sutton, Kurt M. Bohren, Yong Wang, Srinivas Chamakuri, Ruihong Chen, John M. Hakenjos, Ravikumar Jimmidi, Katarzyna Kent, Feng Li, Jian-Yuan Li, Lang Ma, Chandrashekhar Madasu, Murugesan Palaniappan, Stephen S. Palmer, Xuan Qin, Matthew B. Robers, Banumathi Sankaran, Zhi Tan, Yasmin M. Vasquez, Jian Wang, Jennifer Wilkinson, Zhifeng Yu, Qiuji Ye, Damian W. Young, Mingxing Teng, Choel Kim, Martin M. Matzuk

**Affiliations:** 1 Center for Drug Discovery, Department of Pathology & Immunology, Baylor College of Medicine, Houston, TX 77030, USA.; 2 Verna and Marrs McLean Department of Biochemistry and Molecular Pharmacology, Baylor College of Medicine, Houston, TX 77030, USA.; 3 Department of Molecular and Human Genetics, Baylor College of Medicine, Houston, TX 77030, USA.; 4 Promega Corporation, Madison, WI 53711, USA.; 5 Molecular Biophysics and Integrated Bioimaging, Berkeley Center for Structural Biology, Lawrence Berkeley National Laboratory, Berkeley, CA 94720, USA.

## Abstract

Men or mice with homozygous serine/threonine kinase 33 (*STK33*) mutations are sterile owing to defective sperm morphology and motility. To chemically evaluate STK33 for male contraception with STK33-specific inhibitors, we screened our multibillion-compound collection of DNA-encoded chemical libraries, uncovered potent STK33-specific inhibitors, determined the STK33 kinase domain structure bound with a truncated hit CDD-2211, and generated an optimized hit CDD-2807 that demonstrates nanomolar cellular potency (half-maximal inhibitory concentration = 9.2 nanomolar) and favorable metabolic stability. In mice, CDD-2807 exhibited no toxicity, efficiently crossed the blood-testis barrier, did not accumulate in brain, and induced a reversible contraceptive effect that phenocopied genetic *STK33* perturbations without altering testis size. Thus, STK33 is a chemically validated, nonhormonal contraceptive target, and CDD-2807 is an effective tool compound.

In the past 60 years, the world’s population has increased by more than 2.6-fold, growing from 3 billion people in 1960 to 8 billion in 2022, with projections of reaching 9 billion by 2037 (1, 2). Increased population growth leads to unpredictable outcomes for future children (3). Contraception is an important strategy for family planning, particularly individual choice of when and whether they plan to conceive and how many children they will have (2, 4). However, there have been limited breakthroughs in contraception in recent decades. Despite this clear need for more affordable, long-acting, reversible, and safe contraceptives, there are no effective oral contraceptive pills available for men (4–7); clinical trials in men are ongoing for hormonal analogs (progestogenic androgens) or mixtures (8, 9), and some are revealing promising results in phase 1 and 2 studies (10, 11). Although more than 800 knockouts of testis-enriched genes have been created and about 250 of them have revealed a male fertility phenotype (12, 13), few small-molecule (nonhormonal) inhibitors of these validated targets have been developed to date, and even fewer of them have demonstrated a contraceptive effect in preclinical models (14–20).

There are 538 kinases within the human genome (21, 22); however, only ~15% of these kinases have US Food and Drug Administration (FDA)–approved inhibitor drugs or drug candidates (23–26). Most approved kinase inhibitors have been developed for treating cancer. Nevertheless, given their pivotal roles in human biology, kinases are attractive targets that extend beyond cancer. Traditional drug discovery paradigms based on high-throughput screening are costly and inefficient, which attenuates the development of kinase inhibitors to enhance human health. As such, targeting kinases offers a vast number of therapeutic opportunities. A subset of 160 kinases would benefit from efficient small-molecule development because they are poorly understood or studied and are hence designated as “dark kinases” (27). One such dark kinase is STK33, which is evolutionarily conserved in mammals, birds, reptiles, fish, and chordates, and testis-enriched (fig. S1) (13, 28) and expressed in pachytene spermatocytes through transformation of spermatids (29, 30). STK33 is specifically required in male germ cells for spermatid differentiation, and *Stk33* knockout male mice are sterile secondary to teratozoospermia and sperm immotility (30). Men in a single family were discovered to have a frameshift mutation in the *STK33* gene leading to infertility (31) that phenocopied the *Stk33* knockout mice (30). Mice and men with these *STK33* mutations are grossly normal (30, 31), without marked perturbations in testis size (30). STK33 is therefore considered a viable target with minimal safety concerns for contraception in men. STK33 inhibitors have been described (32–34), but none are STK33 specific for in vivo chemical perturbation of STK33 function.

## Discovery of potent STK33 inhibitors from DNA-encoded chemical library screening and hit optimization

**Fig. 1.**
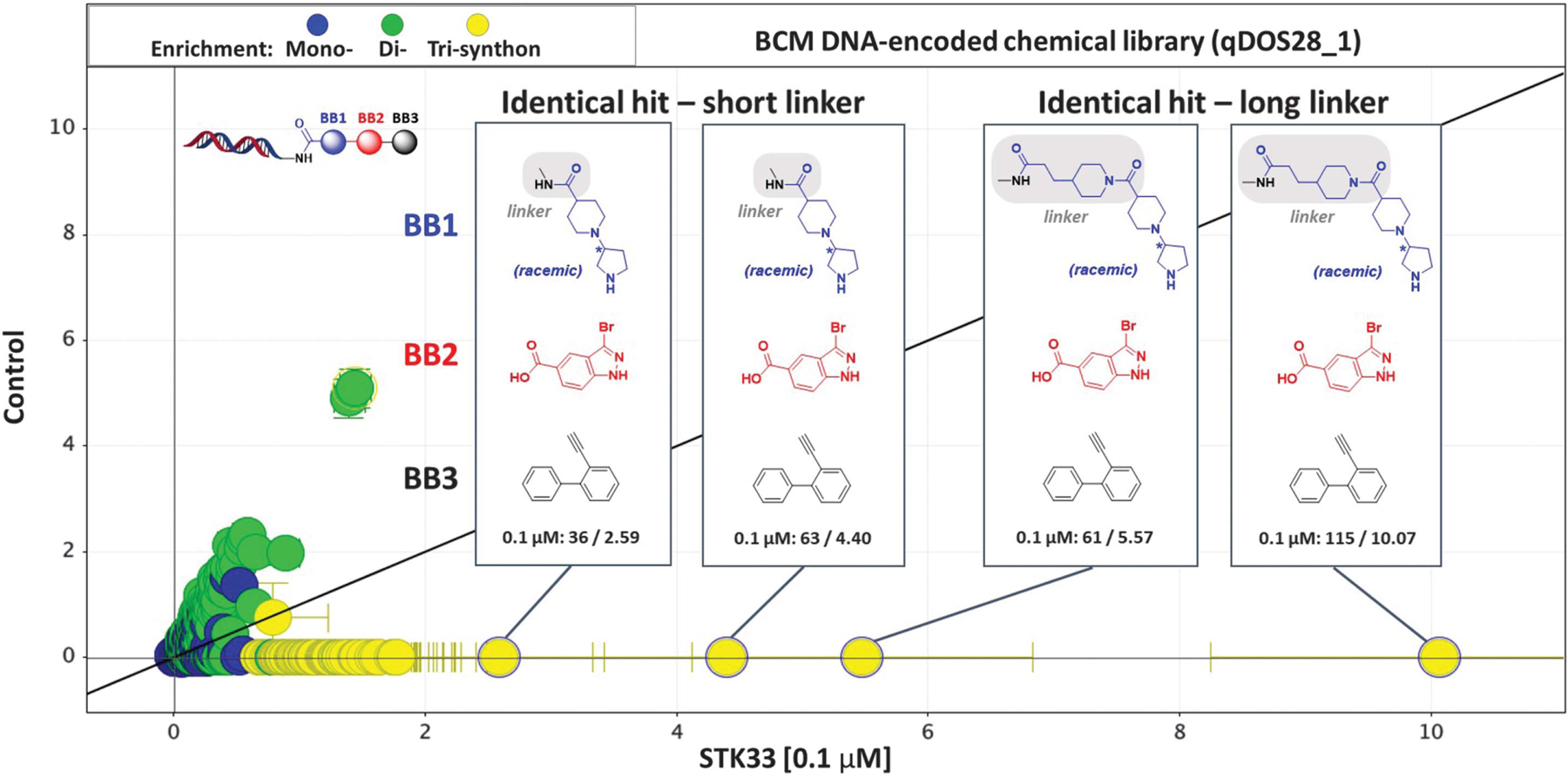
DEC-Tec selection. Enrchment profile of Baylor College of Medicine (BCM) DNA-encoded chemical library qDOS28_1 against STK33 at 0.1 µM (*x* axis, z-score) versus no target control (*y* axis, z-score). A series of hit compounds were identified with similar building block 1 (BB1 in blue; attached to the DNA), same building block 2 (BB2 in red), and same building block 3 (BB3 in black). The enrichment of each tri-synthon is shown as sequencing counts/z-score at 0.1 µM in the box.

**Fig. 2.**
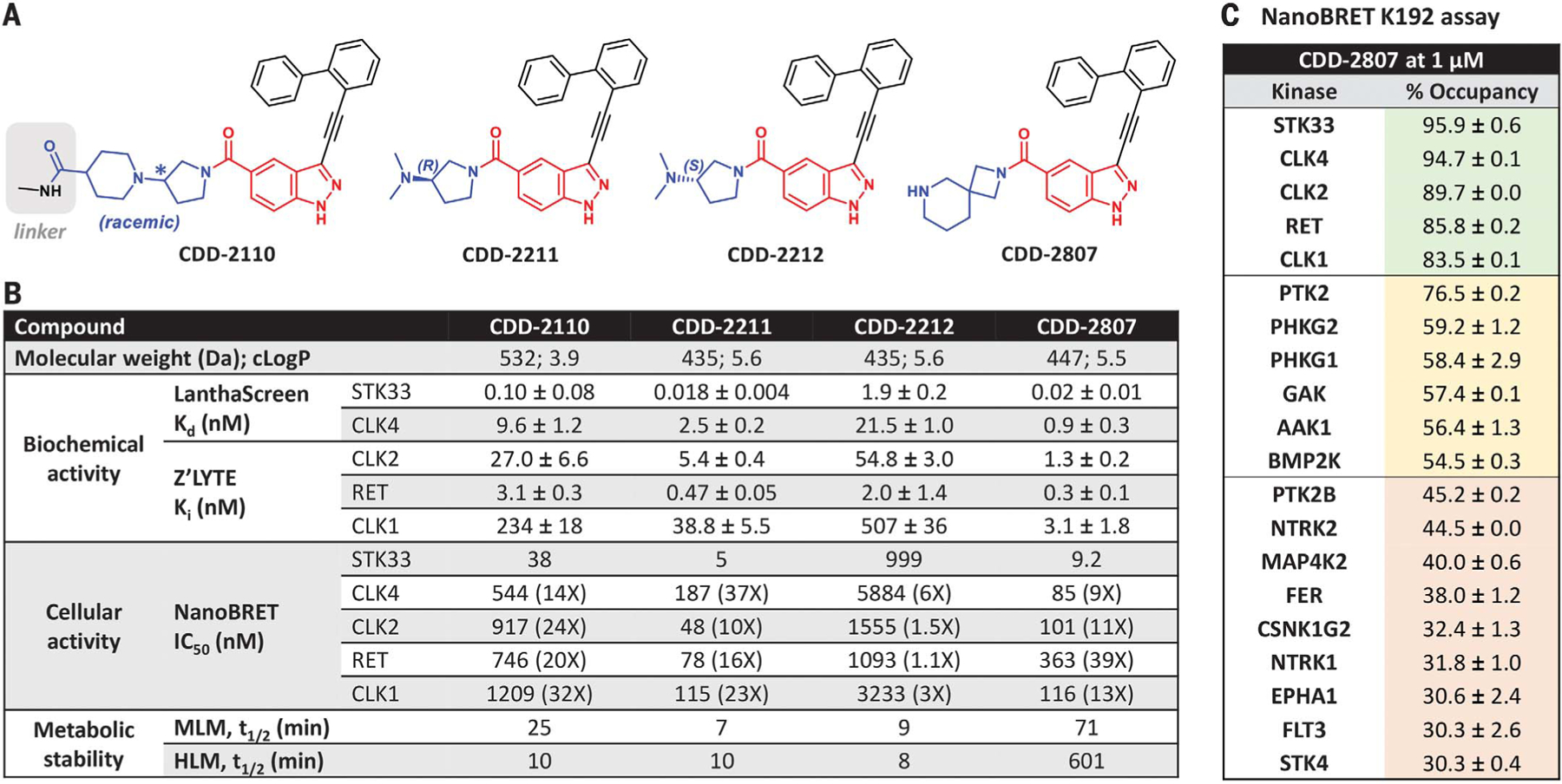
STK33 hits, analogs, and biological characteristics. (**A**) Chemical structures of CDD-2110, CDD-2211, CDD-2212, and CDD-2807. CDD-2110 is the hit with a short linker enriched in the STK33 selection. Blue, red, and black moieties correspond to BB1, BB2, and BB3, respectively. (**B**) Chemical properties, biochemical activity, cellular activity, and metabolism data for CDD-2110, CDD-2211, CDD-2212, and CDD-2807. Dissociation constant (*K*_d_) and inhibition constant (*K*_i_) values were calculated from LanthaScreen binding assay and Z’-LYTE assay, respectively. Dose-responses were done with at least five different concentrations in duplicate, and parameters (*K*_d_ and *K*_i_) optimized by calculations described in the supplementary materials are given with standard errors. Half-maximal inhibitory concentration (IC_50_) values were calculated from the NanoBRET assay. Half-life (*t*_1/2_) was measured using either MLM or HLM stability assays; assay data >60 min is an extrapolated estimate. (**C**) Summary of CDD-2807 fractional occupancy values for kinases with >30% occupancy (red: 30 to 49.9%; yellow, 50 to 79.9%; green: 80 to 100%) from NanoBRET K192 assay performed at 1 µM.

To identify small-molecule binders to the kinase domain (KD) of STK33, we used DNA-encoded chemistry technology (DEC-Tec) (35), which our group (36) and others (37–39) have employed to uncover potent and selective kinase inhibitors. Using full-length His-tagged STK33 protein at either 0.1 or 0.5 µM [with or without staurosporine, a broad-spectrum adenosine 5′-triphosphate (ATP)–competitive kinase inhibitor], we performed selections of 36 or 45 distinct libraries, respectively, each containing 3.9 billion specific DNA-encoded molecules. From library qDOS28_1 in both selection experiments, we discovered enriched hits that shared identical building block 2 (BB2) and building block 3 (BB3) and similar or identical building block 1 (BB1) that differed only in the linker attached to DNA (Fig. 1 and fig. S2, A and B). These hits were absent in the selection performed in the presence of staurosporine, indicating competitive binding of the hits. The hits with a short linker, CDD-2110 (Fig. 2A), and a long linker, CDD-3348 (fig. S3A), were synthesized off-DNA and confirmed to be potent binders of STK33 in a LanthaScreen binding assay performed at ThermoFisher [dissociation constant (*K*_d_) = 0.1 nM for CDD-2110 and *K*_d_ = 0.06 nM for CDD-3348; Fig. 2B and fig. S3B] and a NanoBRET (NB) target engagement intracellular kinase assay performed in-house [half-maximal inhibitory concentration (IC_50_) = 38 nM (NB) for CDD-2110 and IC_50_ = 169 nM (NB) for CDD-3348; Fig. 2B and fig. S3B]. Compared to published STK33 inhibitors [compound 1 (*K*_d_ = 1.7 nM and IC_50_ = 750 nM (NB)], ML281 [*K*_d_ = 39.6 nM and IC_50_ = 7710 nM (NB)], and BRD8899 [*K*_d_ = 1.2 nM and IC_50_ = 11,800 nM (NB) (fig. S3, A and B)] (32–34), CDD-2110 is 12- to 400-fold more potent in biochemical assays and 20- to 310-fold more potent in cellular assays.

CDD-2110 and CDD-3348 are potent but possess racemic formats, have large molecular weights (>500 Da), and are metabolically labile in mouse liver microsomes (MLM) and human liver microsomes (HLM) (Fig. 2B and fig. S3B). To potentially overcome these drawbacks, we synthesized and assayed two truncated enantiomers, CDD-2211 (*R*-isomer) and CDD-2212 (*S*-isomer) (Fig. 2A), with CDD-2211 revealing outstanding inhibitory activity against STK33 in LanthaScreen (*K*_d_ = 0.018 nM) and NanoBRET (IC_50_ = 5 nM) assays when compared to the antipode CDD-2212 [*K*_d_ = 1.9 nM and IC_50_ = 999 nM (NB)], CDD-2110, and CDD-3348 (Fig. 2B and fig. S3B); however, CDD-2211 was labile (*t*_1/2_ ≤10 min; Fig. 2B and fig. S3B) in MLM and HLM assays.

Further medicinal chemistry efforts were invested (figs. S4 to S14) to improve metabolic stability of this hit series. CDD-2807 (Fig. 2A) emerged as a potent STK33 inhibitor biochemically (*K*_d_ = 0.02 nM) and cellularly (IC_50_ = 9.2 nM) and demonstrated excellent metabolic stability [*t*_1/2_ >60 min in MLM and HLM (Fig. 2B and fig. S15)]. To access cellular kinome-wide selectivity, CDD-2807 was subjected to NanoBRET K192 assay (Promega) (40, 41) at a dose of 1 µM against 192 full-length protein kinases in human embryonic kidney 293 (HEK293) cells. In this assay, CDD-2807 engaged STK33 with highest affinity (95.9% occupancy), although other kinases [CDC-like kinase 4 (CLK4), CLK2, rearranged during transfection (RET), and CLK1] were >80% engaged by CDD-2807 (Fig. 2C). Compared to CDD-2807, biochemical and cellular inhibition against CLK4 [*K*_d_ = 0.9 nM and IC_50_ = 85 nM), CLK2 inhibition constant (*K*_i_) = 1.3 nM and IC_50_ = 101 nM], RET (*K*_i_ = 0.3 nM and IC_50_ = 363 nM), and CLK1 (*K*_i_ = 3.1 nM and IC_50_ = 116 nM), CDD-2807 was the most potent ligand of STK33 (Fig. 2B). Thus, CDD-2807 is a potent, stable, and >9-fold selective STK33 inhibitor versus other kinases (Fig. 2B).

Except for CDD-2212 (*S*-isomer), CDD-2110, CDD-3348, and CDD-2211 (*R*-isomer) showed >10-fold selectivity toward STK33 compared to CLK1, CLK2, CLK4, and RET (Fig. 2B and fig. S3B). Chirality played an important role in the selectivity of CDD-2211 versus CDD-2212. The increased selectivity of CDD-3348 versus CDD-2110 suggested that selectivity toward STK33 could be improved by modification of the BB1 linker.

## Generation of a STK33 cocrystal for structure-guided rational design

**Fig. 3.**
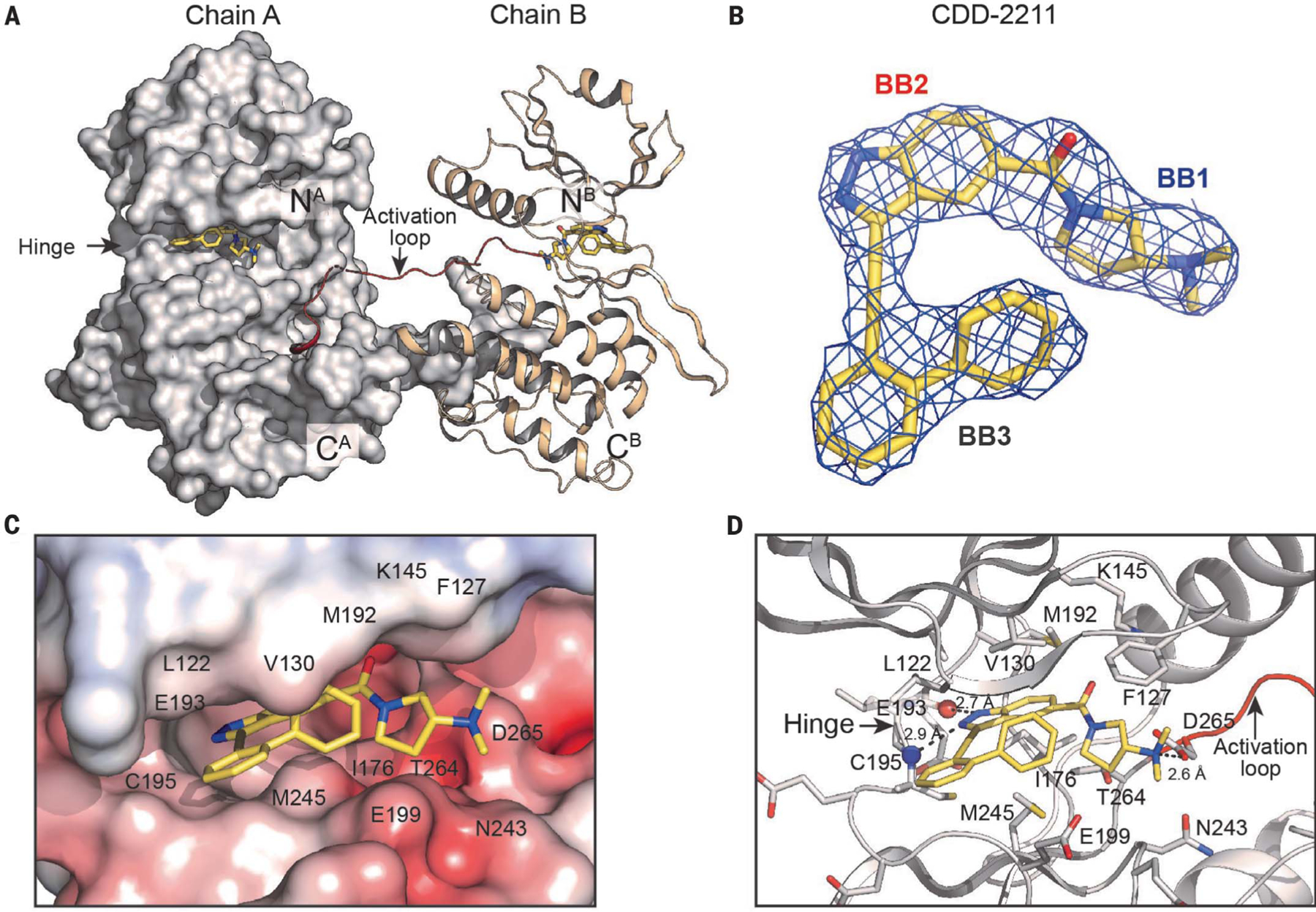
Crystal structure of the STK33/CDD-2211 complex. (**A**) Overall structure of the STK33/CDD-2211 dimer complex. Chain A is shown with surface and chain B in cartoon representation. N and C termini are labeled. The bound CDD-2211 is shown in sticks with its carbon atoms in yellow, oxygens in red, and nitrogens in blue. The activation loop is colored in red. (**B**) *F*o-*F*c omit density for CDD-2211 in the STK33/CDD-2211 complex contoured at 1σ. (**C**) Electrostatic surface of the STK33 active site with CDD-2211. (**D**) Detailed interaction between STK33 and CDD-2211. Hinge residue backbone atoms that form hydrogen bonds with the ligand are shown as spheres. Key interacting residues shown are shown as sticks. Single-letter abbreviations for the amino acid residues are as follows: A, Ala; C, Cys; D, Asp; E, Glu; F, Phe; G, Gly; I, Ile; K, Lys; L, Leu; M, Met; N, Asn; P, Pro; S, Ser; T, Thr; and V, Val.

There is a lack of available structures for STK33, which makes determining the molecular basis for our high-affinity STK33 inhibitors difficult. To address this, we determined the crystal structure of the human STK33 KD in complex with CDD-2211 at 2.7-Å resolution (Fig. 3 and table S1) [Protein Data Bank (PDB) ID: 8VF6]. The STK33/CDD-2211 cocrystal contains one dimer per asymmetric unit (Fig. 3A), with each monomer showing clear density for bound CDD-2211 (Fig. 3B). The two STK33 KD monomers are very similar, showing a root-mean-square deviation (RMSD) value of 0.46 Å between 245 shared Cα atoms (fig. S16). Each monomer shows a nearly identical pose for CDD-2211 (Fig. 3B) bound in the ATP pocket (fig. S16). All residues in the construct are ordered except for a segment of the activation loop (residues 273 to 283 in chain A and 274 to 284 in chain B). As seen in other kinases with the exchanged activation segment conformation (42), the STK33 KD dimer is formed by the activation loop segment and the following two-turn helix with the APE motif from one monomer docking onto a surface formed between αE and αG helices of the other monomer (Fig. 3A).

CDD-2211 binds in the active site formed between the small and large lobes (Fig. 3 and fig. S16). The biphenyl (BB3) binds to the outer lip of the active site near the hinge (Fig. 3C). It interacts with L122 and G123 on top and G198, E199, and M245 at the bottom through van der Waals interactions (Fig. 3, C and D). The indazole (BB2) binds the ribose pocket and forms hydrogen bonds with backbones of E193 and C195 at the hinge region (Fig. 3D). The pyrrolidine with dimethyl amine (BB1) contacts the Mg^2+^ positioning loop, glycine-rich loop, and activation loop. The dimethyl amine forms a hydrogen bond with D265 that normally interacts with Mg^2+^. BB1 is shielded by F127 at the Gly-rich loop (top) and E242 and N243 at the catalytic loop (bottom), and it interacts with these residues through van der Waals interactions.

This cocrystal structure is consistent with structure-activity relationship (SAR) studies from a set of CDD-2211 analogs (fig. S5). Replacement of the -NH group at the indazole (BB2) with the *N*-methyl group resulted in total loss of STK33 activity at 500 nM (fig. S5A), confirming that hydrogen bond interaction at the hinge is critical for STK33 potency. At the biphenyl (BB3), ortho substitution was the best orientation because STK33 potency gradually decreased with movement of the phenyl substituent from the ortho position to meta and para positions (fig. S5B). Moreover, no STK33 activity was observed at 500 nM for analogs with a zero- or one-carbon distance between the indazole and the biphenyl (fig. S5C). Therefore, the interaction at the hinge region involving the indazole fragment, the ortho orientation of the biphenyl group, and the specific distance held by the two-carbon acetylene between the indazole and biphenyl components within CDD-2211 contributed to enhanced potency against STK33.

A computational model of STK33 bound with CDD-2807 was generated on the basis of the crystal structure of the STK33/CDD-2211 complex (fig. S17). The model suggests that the nitrogen of the piperidine moiety at BB1 would interact with the side chain of E199 through a hydrogen bond and its interactions with the active site at BB2 and BB3 would be similar to those in the STK33/CDD-2211 complex.

CDD-2211, similar to CDD-2807, demonstrated some inhibition of off-target kinases CLK1, CLK2, CLK4, and RET (Fig. 2B). To understand cross activity, we aligned crystal structures of RET and CLK4 with the STK33/CDD-2211 complex. The structure comparison showed that most CDD-2211 contacting residues from STK33 are conserved in RET and CLK4 except for E199 and M245 (fig. S18). In RET and CLK4, E199 is replaced with serine (S811 in RET and S247 in CLK4) and M245 is replaced with leucine (L881 in RET and L295 in CLK4), suggesting the potential of increasing the selectivity toward STK33 by modifying BB1 or BB3 moieties of CDD-2211 near E199.

## Validation of chemical perturbation of STK33 for contraception in mice

**Fig. 4.**
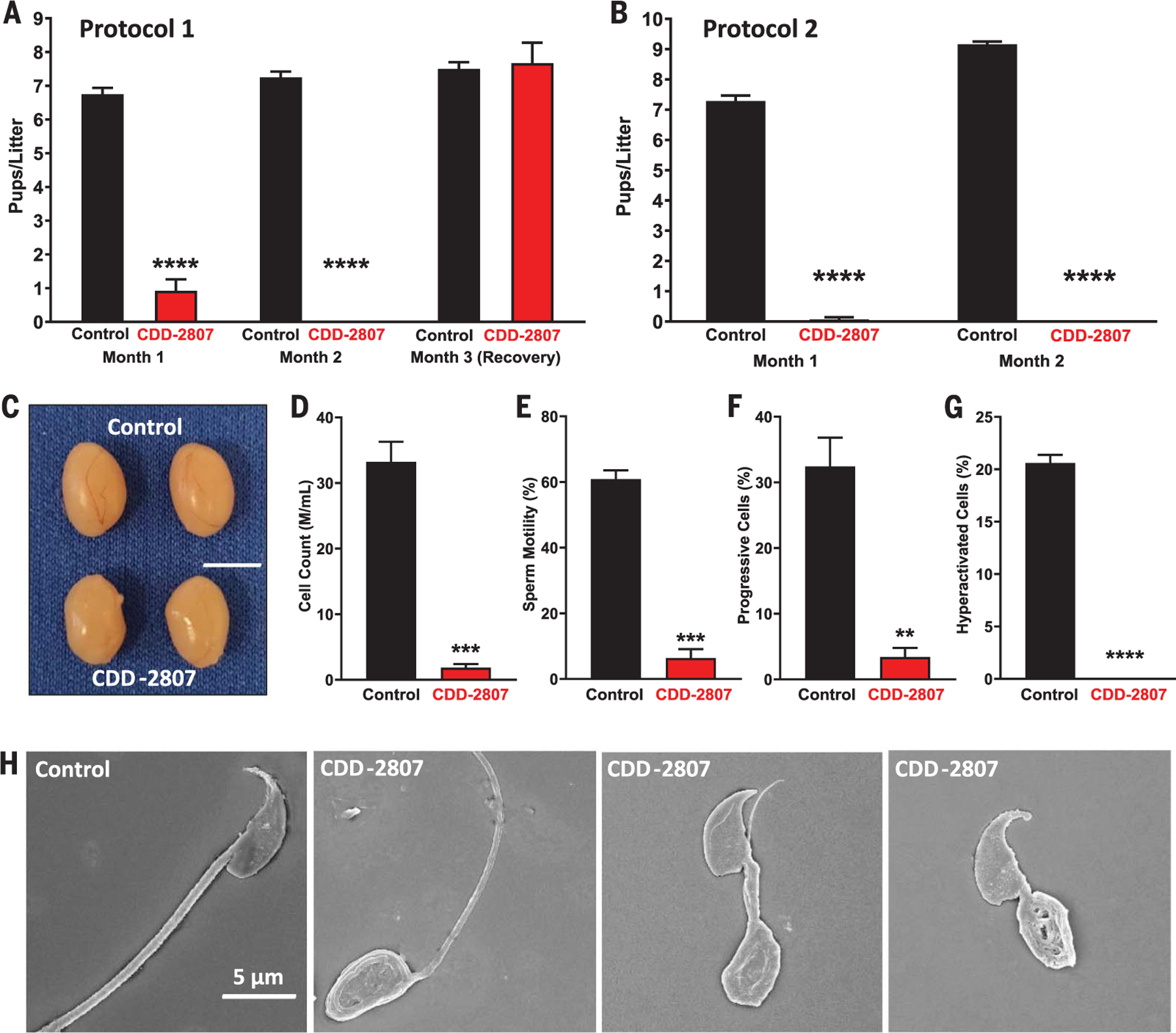
CDD-2807 treatment induces a reversible contraceptive effect. (**A** and **B**) Litter size (mean ± SEM) from the 2-month fertility assessment showed that CDD-2807–treated mice had a significant reduction in fertility in months one and two in protocol 1 [(A); *n* = 6] and protocol 2 [(B); *n* = 7]. Fertility was restored to levels equal to those of controls after halting CDD-2807 treatment for 3 weeks in protocol 1; in protocol 2, fertility was restored an average of 53.5 ± 5.1 days after completion of CDD-2807 treatment compared to the controls (3.5 ± 0.2 days). (**C**) There are no observed morphological differences in testes from control and CDD-2807 treatment in mice from protocol 2 at 63 days; testis size of CDD-2807–treated mice in protocol 2 (87.13 ± 5.87 mg) was not statistically changed versus control males (111.10 ± 6.50 mg). (**D** to **G**) CDD-2807–treated mice (*n* = 3) from protocol 2 at 63 days had a significant decrease in sperm counts (D), motility (E), progressive sperm (F), and hyperactivated sperm (G) compared to control mice (mean ± SEM; *n* = 3). (**H**) For SEM quantification, a minimum of 100 sperm per group were counted and determined to be either normal or abnormal (having head or tail defects). Counts were totaled and then turned into a percentage of normal versus abnormal sperm per group. By SEM analysis, 94.7 ± 2.7% of the sperm in the CDD-2807–treated mice (*n* = 3) in protocol 2 had morphologic defects at 63 days, including head and tail defects as shown, compared to only 10.9 ± 2.7% abnormal sperm for the controls (*n* = 3). Scale bars: 0.5 cm (C), 5 mm (H). **P < 0.01, ***P < 0.001, ****P < 0.0001.

STK33 absence is associated with asthenozoospermia (31), but other studies suggest a role of STK33 in tumorigenesis (43–46) and regulation of extracellular signal–regulated kinase (ERK) signaling pathways (47). On the basis of molecular modeling, mouse and human STK33 have identical ATP binding pockets (fig. S19), consistent with evolutionary conservation and function of STK33. To determine the consequences of STK33 inhibition in vivo, we evaluated reproductive outcomes in male mice dosed with CDD-2807. Because CDD-2807 has a plasma *t*_1/2_ of >11 hours with dose-dependent increase of the maximum concentration (*C*_max_) and area under the curve (AUC) when delivered intraperitoneally versus oral delivery (fig. S20 and table S2), adult male mice were subjected to two protocols (fig. S21). In protocol 1, adult male mice (*n* = 6 per cohort) were treated with vehicle control or CDD-2807 at 15 mg kg^−1^ intraperitoneally twice per day for 21 days, subsequently housed continuously with fertile female mice of reproductive age (two per male), and then evaluated at day 45 for effects of CDD-2807 on the male reproductive tract (after the first litter was born) or continued to be housed with the females until day 66 after the completion of the 60-day breeding data collection (fig. S21A). In protocol 2, adult male mice (*n* = 7 per cohort) were treated with vehicle control or CDD-2807 at 50 mg kg^−1^ once per day for 21 days, subsequently housed continuously with females (two per male), and then evaluated at later time points (fig. S21B). During the period of CDD-2807 or control treatment, there were no deaths and no significant weight changes in the two cohorts of mice (fig. S22), suggesting that CDD-2807 is safe. Whereas the six control-treated males in protocol 1 sired an average of 1.83 ± 0.07 litters per male and 6.75 ± 0.19 pups per female per litter in month 1, only four of the six CDD-2807–treated males sired litters (1.0 ± 0.37 litters per male) and at a lower 0.92 ± 0.34 pups per female per litter (Fig. 4A). In month 2, litters were born to all females housed with the six control males (2.0 ± 0 litters per male; 7.25 ± 0.17 pups per female per litter) (Fig. 4A). By contrast, no litters were produced by females housed with males who received CDD-2807 (Fig. 4A). For protocol 2 mice, all seven control males sired litters from both females in month 1 (7.29 ± 0.18 pups per litter per female), whereas only one female became pregnant and delivered a single pup from the seven CDD-2807–treated males (50 mg kg^−1^ day^−1^) (0.07 ± 0.07 pups per litter per female) (Fig. 4B). CDD-2807–treated males did not sire any litters in month 2, despite their ability to mate. Thus, CDD-2807 delivery in both protocols induced a contraceptive effect.

For males evaluated at day 45 of CDD-2807 treatment on protocol 1 or day 63 on protocol 2, CDD-2807 easily crossed the blood-testis barrier (89.6 ± 14.1 ng or 114.3 ± 7.9 ng of CDD-2807 per milligram of tissue, respectively), whereas it was extremely low in the brains of the protocol 1 males (0.28 ± 0.09 ng of CDD-2807 per milligram of tissue) and undetectable in the protocol 2 males (fig. S23A). Lack of detection of CDD-2807 in protocol 2 mouse brains may be due to collection at 24 hours after dosing versus 12 hours in the protocol 1 mice. Despite the contraceptive effect of CDD-2807, testis size of CDD-2807–treated mice in either protocol was statistically unchanged versus control males (Fig. 4C and fig. S23B). Serum concentrations of alanine aminotransferase (ALT) or aspartate aminotransferase (AST) did not differ in protocol 1 or protocol 2 between groups (table S3), and low levels of CDD-2807 were observed in lung, a tissue that expresses STK33 (figs. S1 and S23A), confirming the relative safety of CDD-2807.

To further understand the cause of the contraceptive effect of CDD-2807, we analyzed sperm from the cauda epididymis of each mouse using computer assisted sperm analysis (CASA). Although sperm counts were lower in the controls in protocol 1 mice versus CDD-2807–treated mice (fig. S23C), sperm motility (fig. S23D), progressive sperm (fig. S23E), and hyperactivated sperm (fig. S23F) were reduced in the CDD-2807–treated mice. In protocol 2, sperm counts (Fig. 4D), sperm motility (Fig. 4E), progressive sperm (Fig. 4F), and hyperactivated sperm (Fig. 4G) were reduced in the CDD-2807-treated mice versus control mice. Treatment with 50 mg kg^−1^ day^−1^ for 7 days (Protocol 3) did not alter mouse body weights (control, 26.5 ± 0.08 g; CDD-2807, 27.05 ± 0.79 g), testis size (fig. S23G), or sperm counts (fig. S23H), but there were statistically significant reductions in sperm motility (fig. S23I), progressive sperm (fig. S23J), and sperm hyperactivation (fig. S23K). Histologically, the testes and epididymis of the CDD-2807–treated mice in protocol 1 showed no detectable defects (fig. S24). However, histology from protocol 2 mice showed round spermatids in the epididymis (fig. S25), indicating that higher dosages over a longer period did affect spermatogenesis. By scanning electron microscopy (SEM), sperm analysis of the CDD-2807–treated mice in protocol 2 demonstrated teratozoospermia (Fig. 4H). Thus, low dose (CDD-2807 at 15 mg kg^−1^ twice per day for 45 days) or reduced duration (CDD-2807 at 50 mg kg^−1^ day^−1^ for 7 days) appears to cause a functional defect in motility, whereas high dose (CDD-2807 at 50 mg kg^−1^ day^−1^ for 63 days) causes both functional and morphologic defects, consistent with the *Stk33* knockout and likely a direct role of STK33 in phosphorylation of fibrous sheath proteins A-kinase anchoring protein 3 (AKAP3) and AKAP4 and/or other STK33 targets (48). These finding also suggest that a “loading dose” of CDD-2807 over 7 days to reach ~24 ng of CDD-2807 per milligram of testis (fig. S23A) and then less frequent, lower-maintenance doses could maintain the contraceptive effects in mice and men.

To evaluate the reversibility of the effects of CDD-2807 on male fertility, drug and control treatment of mice in protocol 1 was halted at day 63, males were removed from females for 21 days, and males were caged with new adult females (Fig. 4A). The four control males sired offspring from the females (7.50 ± 0.20 pups per litter per female); likewise, the three previously treated CDD-2807 cohort males sired offspring from the females (7.67 ± 0.61 pups per litter per female) within the first month of breeding. The time to fertility resumption was measured directly after treatments in protocol 2 mice. On average, it took control mice 3.5 ± 0.2 days to sire their first litters, and CDD-2807–treated mice took an average of 53.5 ± 5.1 days to sire their first litters after treatment. Thus, the effects of CDD-2807 are reversible regardless of the dosage used.

## Discussion

Our studies demonstrate that DEC-Tec can identify kinase inhibitors that can be used for contraceptive development. In the present example, we identified a hit from DEC-Tec screening that was developed into a potent inhibitor of STK33, showed that our inhibitor (CDD-2807; molecular weight of 447 Da) easily crossed the blood-testis barrier to cause infertility, and demonstrated that the contraceptive effects were reversible. Additionally, a proteolysistargeting chimera (PROTAC)–based chemical knockdown approach (49) is possible for testisspecific degradation of STK33 as suggested recently (50). Lastly, CDD-2807 is an excellent chemical probe that can be used to perform additional safety and toxicological studies in vivo, including analysis in nonhuman primates, and to investigate STK33 signaling in various contexts other than male contraception. This work demonstrates that kinases can serve as beneficial targets for treating human conditions beyond oncology indications.

## Supplementary Material

Ku_et_al_2024_Science_SM

MDAR Reproducibility Checklist

## Data Availability

All data associated with this study are present in the main text or the supplementary materials. Protein coordinates and structure factors of the STK33/CDD-2211 complex have been deposited in the RCSB PDB under code 8VF6.
