# Supplementary material for: Reversible male contraception by targeted inhibition of serine/threonine kinase 33
Source: Science. Author manuscript; Available in PMC 2025 Feb 20. (PMC11842024; doi:10.1126/science.adl2688)
Supplement: Ku_et_al_2024_Science_SM [file NIHMS2052210-supplement-Ku_et_al_2024_Science_SM.pdf]

Science
AAAS

Supplementary Materials for

Reversible male contraception by targeted inhibition of serine/threonine kinase 33

Angela F. Ku *et al*.

Corresponding author: Martin M. Matzuk, mmatzuk@bcm.edu

Science **384**, 885 (2024)
DOI: 10.1126/science.adl2688

The PDF file includes:

Materials and Methods
Figs. S1 to S25
Tables S1 to S3
References

Other Supplementary Material for this manuscript includes the following:

MDAR Reproducibility Checklist

Materials and Methods

DEC-Tec affinity selections. For these studies, two independent DEC-Tec selections of our in-house libraries were performed using full length recombinant human STK33 protein with an N-terminal His6 tag (Eurofins #14-671). In Experiment 1, selection of 45 libraries (3.940 billion compounds) was performed with STK33 protein at 0.5 μM in the absence or presence of staurosporine (10 μM). In Experiment 2, selection of 36 libraries (3.936 billion compounds) was performed with STK33 protein at 0.1 μM. Selection methods and informatics were performed as described previously (*36*, *51–58*).

STK33 KD protein expression and purification for crystallography. Human STK33 KD (GenBank accession number CAC29064.1, residues 99−383) with tobacco etch virus (TEV) cleavage site and cGFP-Strep-tagII was expressed in insect SF9 cells (ThermoFisher Scientific). SF9 cells were cultured in SF900III medium (Gibco) to density of about 2.5×10^6 cells/mL and infected with STK33 KD baculovirus at multiplicity of infection of 3. Cells were harvested 48 h after infection; pellets were kept at −80°C. For purification, frozen cell pellet was resuspended in lysis buffer (25 mM Tris, pH 8.0, 150 mM NaCl, and 1 mM TCEP) and lysed using homogenizer (Avestin). After centrifugation at 48000 *g* for 1 h, the soluble fraction was loaded onto pre-equilibrated Strep-Tactin Superflow resin (IBA Lifesciences GmbH) and eluted with elution buffer (25 mM Tris, pH 8.0, 150 mM NaCl, 1 mM TCEP, and 2 mM desthibiotin). Fractions containing the protein were treated with TEV protease (1:60 weight ratio, overnight) to remove the GFP tag and the mixture was subjected to Superdex 75 Increase 10/300 GL gel filtration column (Cytiva) equilibrated with the abovementioned lysis buffer. The peak corresponding to the estimated molecular mass of approximately 70 kDa was pooled and used for co-crystallization.

Crystallization, data collection, and structure solution. STK33 KD was co-crystallized with CDD-2211 by hanging drop vapor diffusion method. For crystallization, the purified STK33 at 1.8 mg/mL was mixed with 6 molar excesses of CDD-2211. The protein-inhibitor mixture was concentrated using Amicon Ultra-15 centrifugal filters (Millipore Sigma) to 14 mg/mL. Mosquito (SPT labtech) was used to dispense equal volumes of protein and reservoir (250 nL each) against 70 µL reservoir buffer in 96-well crystallization tray (96-Well clear polystyrene microplate from SPT labtech). Crystals were observed after 2 days in drop using JCSG plus A12 condition (0.2 M potassium nitrate, 20% w/v polyethylene glycol 3350). The diffraction data was collected at Advanced Light Source (ALS), Beam Line 5.0.2 (UC Berkeley, USA) at wavelength (λ) = 1.0000 Å, by using an Dectris Pilatus 6M detector. The data were integrated and scaled by using iMosflm and SCALA, respectively (*59*, *60*). The crystal structure of the STK33/CDD-2211 complex was determined by molecular replacement (*61*) using a monomeric model of STK33 KD (residues 99−383) generated by Alpha fold (*62*) as a search model. CDD-2211 was traced and fitted manually into electron density by using COOT. The final models have gone through several rounds of refinement using phenix.refine (*63*) followed by manual model building using COOT (*64*). For all structural analysis and preparation of figures, the visualization program PyMOL was used (*65*).

Computational modeling of STK33/CDD-2807 complex. The crystal structure complex of STK33/CDD-2211 generated in house was used as a starting point to build the STK33/CDD-2807 model. The STK33 protein structure was prepared using Schrödinger Suite Release 2022-1 (*66*). A grid was generated at the ATP binding site for subsequent docking studies. CDD-2807 was

prepared using the LigPrep (*67*) program, and its protonation state was assigned using the Epik (*68*) program within the Schrödinger software suite. The prepared CDD-2807 was subjected to docking into the ATP binding pocket of STK33 using the Glide (*69*) program in the extra precision mode. The resulting docking poses were then extracted and visualized using the Maestro (*70*) program.

In vitro biochemical assays. Several small molecule assays were outsourced to SelectScreen Services (ThermoFisher) for STK33 and for off-target kinases (data sets are available on demand). These assays are LanthaScreen (binding assay) and Z'-LYTE (activity assay). Dose-responses were done with at least 5 different concentrations in duplicates, and parameters were optimized by calculations described below and provided with standard errors.

Calculation of K_d values for kinase inhibitors that bind to the ATP pocket of a kinase from LanthaScreen binding data. DynaFit (*71*) was used to fit complex equilibria where a tracer is displaced by a dilution series of a kinase inhibitor. This method relies on the concentration of inhibitor, as well as the concentration of kinase and tracer, and K_d of the tracer. The latter three parameters are tabulated by ThermoFisher for each kinase in the LanthaScreen assay panel.

Calculation of K_i values for kinase inhibitors from Z'-LYTE inhibition data. The Morrison equation with [ATP] = K_m^{app} for competitive inhibition (GraphPad Prism 7, San Diego, CA) was used to get K_i values. Since K_d and K_i values are defined the same way, in other words, $K_d = [E][I] / [EI] = K_i$, K_d and K_i values can be compared.

NanoBRET Target Engagement intracellular kinase assay. HEK293T cells were cultured in DMEM (Gibco11965-092) with 10% FBS and 1% Pen-Strep (Gibco 15140-122) in 5% CO_2, 37°C. The cell line underwent authentication by short tandem repeat (STR) profiling at Cytogenetics and Cell Authentication Core (CCAC) at MD Anderson Cancer Center. NanoBRET Target Engagement intracellular kinase assay, K-10 assay (Cat. # N2641), K-9 assay (Cat. # N2631), transfection reagent FuGENE HD (Cat. # E2311), transfection carrier DNA (Cat. # E4881), NanoLuc-STK33 fusion vector (Cat. # NV2111), NanoLuc-RET fusion vector (Cat. # NV1951), NanoLuc-CLK1 fusion vector (Cat. # NV1131), NanoLuc-CLK2 fusion vector (Cat. # NV1141), and NanoLuc-CLK4 fusion vector (Cat. # NV1151) were purchased from Promega. The measurement of test compound engagement with cellular target protein was performed in a 384-well format according to manufacturer's instructions. HEK293 cells were transiently transfected with NanoLuc (STK33, RET, CLK1, CLK2, and CLK4) fusion vectors. After 36 hours of transfection, cells were treated with a fixed concentration of tracer (recommended by Promega) followed by compounds (inhibitors) for 2 hours of incubation. To determine the test compound affinity, cells were titrated with varying concentrations of test compounds ranging from 40 µM to 0.4 nM at a fixed tracer concentration. Staurosporine was measured in parallel as a positive control. Freshly prepared NanoBRET Nano-Glo substrate plus extracellular NanoLuc inhibitor was then added to initiate the subsequent bioluminescence resonance energy transfer (BRET) measurements using a CLARIOstar Plus BMG LABTECH plate reader. Data analysis was done by measuring the ratio of acceptor emission to donor emission (BRET ratio) and normalized by subtracting no-tracer-control-background. IC_{50} values were obtained by nonlinear regression using a four-parameter equation in GraphPad Prism 7 (San Diego, CA).

NanoBRET Target Engagement K192 assay. HEK293 cells (ATCC) were cultured in DMEM (Gibco) + 10% FBS (Seradigm) and incubated in a humidified 37°C/5% CO_2 incubator. The NanoBRET Target Engagement K192 Kinase Selectivity System (Promega) contains 192 unique transfection-ready NanoLuc/Kinase fusions, pre-diluted in either transfection carrier DNA or relevant cyclin DNA. These NanoLuc/Kinase fusion constructs were transfected into HEK293 cells using Fugene HD (Promega) according to the manufacturer's protocol. Briefly, 10 µL of a 20 µg/mL DNA solution was added to white, TC-treated 96-well assay plates (Corning), followed by 30 µL of a diluted Fugene HD solution (20 µL Fugene/1 mL Opti-MEM). After transfection complex formation, HEK293 cells were resuspended at 3.3×10^5 c/mL in Opti-MEM + 1% FBS and added at a volume of 60 µL per well, to result in approximately 20000 cells/well. Cells were incubated in a humidified 37°C/5% CO_2 incubator for 20 hours. Tracer K-10 (Promega) was prepared at a 100× concentration in DMSO (Sigma) and diluted to a working concentration of 10× in tracer dilution buffer (12.5 mM HEPES, 31.25% PEG400, pH 7.5) (Promega). Test compounds were prepared as concentrated stock solutions in DMSO and diluted in Opti-MEM to prepare 10× working stocks. Cells were equilibrated with test compounds and Tracer K-10 for 2 hours prior to BRET measurements. For target engagement analysis, Tracer K-10 was added to cells at one of 4 different concentrations, ranging from 25 nM to 1 µM, based on target affinity, according to manufacturer's protocol. To measure BRET, NanoBRET NanoGlo Substrate and Extracellular NanoLuc Inhibitor (Promega) were added according to the manufacturer's protocol, and filtered luminescence was measured on a GloMax Discover luminometer equipped with 450 nm BP filter (donor) and 600 nm LP filter (acceptor), using 0.5 s integration time. Milli-BRET units are calculated by multiplying the raw BRET units by 1000.

For Fractional Occupancy determination, the following equation was used:
% Occupancy = $[1-(X-Z)/(Y-Z)]\times100$
Where X = BRET in the presence of the test compound and Tracer K10, Y = BRET in the presence of Tracer K10 only, and Z = Full occupancy, or BRET in the absence of the test compound and Tracer K10. In this case, we used an untagged NanoLuc construct (Promega) for the full occupancy control.

Metabolic stability assay in liver microsomes. The stabilities of CDD-2110, CDD-2211, CDD-2212, CDD-2807, and CDD-3348 in liver microsomes with/without Uridine Diphosphate Glucuronic Acid (UDP-GA, a cofactor of UDP-glucuronosyltransferases) were performed and half-life was calculated as previously reported (*36*). Briefly, each CDD compound (2.0 µM) was incubated in the mouse or human liver microsomes (0.5 mg protein/mL) at 37°C in 1× PBS fortified with NADPH (1.0 mM) and with/without UDP-GA (2.5 mM). Alamethicin (25 µg/mL) as a pore forming agent was used for the glucuronidation (Table S2A). The samples were collected at specific time-points 0, 30, and 60 min in duplicate. The reactions were terminated by adding equivalent volume of ice-cold methanol (CH_3OH) and vortexed. After centrifugation at 15000 *g* for 10 min, 3.0 µL of the supernatant was analyzed by Thermo TSQ Quantis MS coupled with a Thermo Vanquish UHPLC (San Jose, CA) equipped with a Luna C18 column (1 mm × 50 mm, 1.6 µm, Phenomenex, Torrance, CA). The column temperature was maintained at 40°C. CDD-2807 was monitored under the selected reaction monitoring (SRM) mode coupled with a positive electrospray ionization (ESI) source. The SRM ion pairs were 447→321 for CDD-2807.

Pharmacokinetics (PK) of CDD-2807 in mice. The pharmacokinetic studies (*72*) of CDD-2807 were performed at three doses via intraperitoneal (i.p.) injection and oral (p.o.) administration. CDD-2807 was dissolved in 10% Captisol containing 5% DMSO to prepare the solutions of 0.5 mg/mL, 2.5 mg/mL and 5.0 mg/mL. Twelve male mice (strain: C57BL/6NJ, 8−12 weeks) were divided equally and randomly into 4 groups (n = 3 for each dose). One group of mice were administered 5 mg/kg CDD-2807 (i.p., 10 µL/g of 0.5 mg/mL solution), the second group 25 mg/kg CDD-2807 (i.p., 10 µL/g of 2.5 mg/mL solution), the third group 5 mg/kg CDD-2807 (p.o., 10 µL/g of 0.5 mg/mL solution), and the fourth group 50 mg/kg CDD-2807 (p.o., 10 µL/g of 5.0 mg/mL solution). At 0, 5, 10, 15, 30 min, 1, 2, 4, 6, 8, 24 h post-dose, around 20 µL of blood was collected from the tail vein and anti-coagulated by heparin. The blood samples were centrifuged at 2000 *g* for 3 min at 4°C, and the plasma samples were transferred to tubes and stored at −80°C before analysis. All the samples were analyzed by the Thermo TSQ Quantis MS coupled with a Thermo Vanquish UHPLC (San Jose, CA). CDD-2807 was separated on a Luna C18 column (1 mm × 50 mm, 1.6 µm, Phenomenex, Torrance, CA), and eluted by a water-acetonitrile mobile phase system (both containing 0.1% formic acid, *v/v*) at the flow rate of 0.15 mL/min. CDD-2807 was monitored under the SRM mode coupled with a positive ESI source. The SRM ion pairs were 447→321 for CDD-2807. The ion spray voltage was set at 3500 V. High-purity nitrogen was used as the sheath gas (35 arbitrary unit), auxiliary gas (7 arbitrary unit), and high-purity argon was used as the collision gas. The temperatures of the ion transfer tube and the vaporizer were set at 300°C and 275°C, respectively. The concentration of CDD-2807 in plasma was quantified using individual calibration curves. The calibration curve was regressed with a weight of $1/x^2$ with high linearity (r^2 >0.99). PK parameters, including half-time ($t_{1/2}$), area under the plasma concentration–time curve from zero to infinity ($AUC_{0-\infty}$), clearance normalized by the bioavailability (CL/F), volume of distribution normalized by the bioavailability (Vd/F), and the mean residence time (MRT), were calculated by WinNonlin software (Certara, Princeton, NJ) by noncompartmental analysis. The plasma concentration–time curves were plotted in GraphPad Prism 9 (San Diego, CA) as mean ± S.E.M. Because CDD-2807 was more metabolically stable (~10 hours with Phase I metabolism, and ~6 hours with Phases I and II metabolism) in HLM than in MLM (~1 hour with Phase I metabolism, ~50 minutes with Phases I and II metabolism) and is capable of crossing the blood-testis barrier, CDD-2807 could be metabolically more stable in men than mice, allowing us to translate our findings to humans. For the identification of glucuronidation products of CDD-2807 in vivo, the mice were treated with CDD-2807 (25 mg/kg, i.p., n = 4). The plasma samples were collected 2 hours after the treatment and analyzed by a Thermo Q Exploris 120 MS coupled with a Thermo Vanquish UHPLC (San Jose, CA). The possible metabolites and the substrate were separated on a 2.1 mm × 100 mm BEH-C18 column (1.7 µm, Waters, Milford, MA) and ionized by a positive ESI source. MS data were acquired from 80 to 1200 Da in-profile mode. The internal calibrant was used as the reference mass during acquisition.

CDD-2807 concentrations in mouse brain, liver, lung, and testis. Tissue samples harvested from mice on day 45 of protocol 1 and at 24-hour, day 7, and day 63 of protocol 2 were weighed and snap-frozen in liquid nitrogen. The samples were stored at −80°C before analysis. Twenty-five mg of tissue was homogenized in 150 µL of 50% CH_3OH, and 50 µL of homogenate was added to 200 µL of ice-cold CH_3OH containing 0.1 µM of agomelatine (internal standard). The mixture was vortexed and centrifuged at 15000 *g* for 15 min, and supernatants were transferred into sample vials for analysis. CDD-2807 was analyzed by the same method used in the pharmacokinetic study. The concentration of CDD-2807 in the testis and brain was determined by

using individual calibration curves established with the corresponding tissue homogenates from untreated mice. The calibration curve was regressed with a weight of $1/x^2$ with high linearity (r^2 >0.99).

Spatial distribution of CDD-2807 in testis. The testis from the mice treated with CDD-2807 were attached to the Leica CM3050 S cryostat (Leica Biosystems, Deer Park, IL) with water and then sectioned at the thickness of 10 µm at −20°C. The sections were attached to electrically conductive glass slides coated with indium tin oxide (5*75*1.1 mm, 70-100 ohms, Delta Technologies, Loveland, CO). Tissue sections were sprayed with a matrix solution containing 35 mg/mL 2,5-dihydroxybenzoic acid (DHB, Sigma, St. Louis, MO) in 70% aqueous CH_3OH with 0.1% trifluoroacetic acid using an HTX TM-Sprayer (HTX Technologies LLC, Carrboro, NC). Sprayer nozzle temperature was set at 70°C, tray temperature at 40°C, nozzle velocity at 1200 mm/min, track spacing at 2 mm, drying time for 10 sec and nitrogen gas pressure at 10 psi. The data were collected on the Bruker TimsTOF MS MALDI-2 instrument with the resolution at 40 µm/pixel (mass range 100−1000 Da under the positive mode) using DHB ion as the on-line calibrant. The data was analyzed, and the images were plotted by SciLs Lab software (Bruker, Billerica, MA). The ions were extracted within mass accuracy of ±3.5 ppm.

Alanine transaminase (ALT) and aspartate transaminase (AST) assays. On day 45 of protocol 1 and day 7 and day 63 of protocol 2, three mice from each protocol were euthanized, and blood was collected through cardiac puncture. The serum was isolated by centrifuging the whole blood samples at 1500 *g* for 10 min and then maintained on wet ice before analysis. ALT and AST levels were measured in duplicates using assay kits (StanBio, Boerne, TX) according to the manufacturer's instructions. ALT and AST levels were determined using the rate of NADH oxidation, which was quantified by measuring absorbance at 340 nm using a CLARIOstar plate reader (BMG Labtech in Cary, NC). ALT and AST were tested for significance at multiple collection time points, tests for significance included a test for normality (Shapiro-Wilk test) and following the test for normality were then analyzed using an unpaired t-test with significance determined by an alpha of $P = 0.05$. For the ALT and AST measurements we did not have observe any significant differences between groups.

Fertility analysis. Sexually mature male C57/129 hybrid mice were randomly assigned to either the vehicle control treatment or the CDD-2807 treatment groups. Drug formulation and injections were as follows: for protocol 1 mice receiving the 15 mg/kg CDD-2807 twice daily dosing had the compound dissolved in DMSO, which was injected at 5% of the final volume in 10% Captisol and water. For protocol 2 mice receiving a single daily dose of 50 mg/kg CDD-2807, the compound was dissolved in DMSO and injected at 5% in 20% Captisol and water. All injections were administered as i.p. injections, and for the 15 mg/kg CDD-2807 and DMSO control mice, the injections were delivered in the morning and the evening. Mice in the 50 mg/kg dosing received i.p. injections once in the evening. Weights were measured daily prior to drug administration and monitored throughout the entirety of the injection period. Injections were given for 21 days prior to introduction of female mice. During the fertility assessment, males were housed continuously with two C57/129 sexually mature females for 45 days to generate approximately two months of fertility data. During the fertility assessment, the total number of pups born per litter sired by each male were counted and the total number of litters and pups sired per male over the entire trial was calculated. Mean pup number and SEM were used to determine differences in average number of

pups sired using a t-test in GraphPad Prism 9 (San Diego, CA). All mouse experiments were performed according to the guidelines from the Institutional Animal Care and Use Committee at BCM (protocol AN-716).

Histological analysis. Details of the process for obtaining histology images of testes tissue and epididymis tissue that were collected can be found in this publication (*73*). Briefly, tissue was fixed in Bouin's fixative, washed with 70% ethanol, embedded in paraffin, sectioned at 4-μm thickness, and stained by periodic acid-Schiff (PAS)-hematoxylin.

Computer-assisted sperm analysis (CASA). Sperm were extracted by mincing both segments of the cauda epididymis 25 times with dissection scissors in 1 mL of Enhance Sperm Wash w/Gentamicin (Vitrolife, Sweden) medium. All medium was maintained at 37°C throughout the procedure. After incubation for 15 minutes, the supernatant was diluted, and 6 μL of diluted sample was added into a single chamber of a dual chambered 20 μm-depth Leja semen analysis slide (Spectrum Technologies). Sperm parameters were measured using the Hamilton Thorne CEROS II system. Per sample, a minimum total of 200 sperm were measured, which typically consisted of recording a minimum of five non-overlapping fields. An additional measurement was taken following a 90-minute incubation at 37°C to allow for sperm capacitation.

Sperm analysis for scanning electron microscopy (SEM). Sperm were extracted by mincing the cauda of the epididymis 25 times with dissection scissors in 1 mL of Enhance Sperm Wash w/Gentamicin (Vitrolife, Sweden) medium incubated at 37°C. Sperm were allowed to remain in the medium at 37°C for a minimum of 15 minutes to allow sperm to disperse from the tissue. Media and sperm were then collected and moved to a separate tube and then sperm were centrifuged at 300 *g* for 5 minutes at room temperature, allowing sperm to collect at the bottom without any epididymis segments being present. Medium was removed and sperm cells were resuspended in DPBS to wash. Following the wash, sperm were centrifuged a second time and then resuspended in fresh 2.5% glutaraldehyde/PBS and fixed for 30 minutes at room temperature on a rocker. After fixation, sperm were dehydrated in increasing concentrations of ethanol from 20%, 30%, 50%, 60%, 70%, 90%, and 100%. During each dehydration step, sperm were incubated at room temperature for 15 minutes. After each increase in percentage of ethanol, the density of the sperm changed and required higher speeds for each subsequent centrifugation; when sperm were in 100% ethanol, it required ≥1200 *g* to pellet them. Sperm were incubated overnight in 100% ethanol at 4°C, and the following day the samples were split and then centrifuged at 1500 *g* for 5 minutes at room temperature and then resuspended in 50% tert-butanol/ethanol and incubated for 15 minutes. Samples were then imaged at the Houston Methodist Research Institute Microscopy SEM/AFM core on a FEI Nova NanoSEM 230 instrument.

Materials and instrumentation for the synthesis of compounds. Reagents and solvents purchased from commercial supplies were used as received. All reactions involving air-sensitive reagents were carried out in anhydrous solvents under an atmosphere of nitrogen. Reactions were monitored by thin-layer chromatography (TLC) on Baker-flex silica gel plates (IB2-F) using UV-light (254 and 365 nm) detection or liquid chromatography-mass spectrometry (LC-MS). Column chromatography was carried out using Teledyne ISCO CombiFlash system equipped with either a silica or C-18 column. NMR spectra were recorded at room temperature using a Bruker Avance III HD 600 MHz spectrometer (^{1}H NMR at 600 MHz and ^{13}C NMR at 150 MHz) or Bruker Avance

800 MHz spectrometer (^{1}H NMR at 800 MHz and ^{13}C NMR at 200 MHz). Chemical shifts (δ) are reported in parts per million (ppm) with reference to solvent signals [^{1}H-NMR: $CDCl_3$ (7.26 ppm), CD_3OD (3.31 ppm) and DMSO-d_6 (2.50 ppm); ^{13}C-NMR: $CDCl_3$ (77.00 ppm), CD_3OD (49.15 ppm) and DMSO-d_6 (39.51 ppm)]. Signal patterns are reported as s (singlet), d (doublet), t (triplet), q (quartet), h (heptet), m (multiplet), and br (broad). Coupling constants (*J*) are given in Hz. High-resolution mass spectra (HRMS) were recorded on a Thermo Q Exploris 120 mass spectrometer using ESI under the positive mode.

Experimental procedures and characterization data of the synthesized compounds. The synthesis of compounds was achieved by following the synthetic schemes shown in figs. S6–S9. ^{1}H and ^{13}C NMR spectra of the key compounds (CDD-2110, CDD-2211, CDD-2212, CDD-2807, and CDD-3348) are shown in figs. S10–S14. Purities of assayed compounds were greater than 95% as determined by reverse-phase high performance liquid chromatography analysis.

General synthetic procedures

(a) General procedure for Sonogashira coupling:

An oven-dried microwave vial equipped with magnetic stir bar was charged with halide (0.5 mmol, 1.0 equiv.), substituted alkyne (0.6 mmol, 1.2 equiv.), copper(I) iodide (CuI) (0.05 mmol, 0.1 equiv.), and bis(triphenylphosphine)palladium(II) dichloride ($PdCl_2(PPh_3)_2$) (0.05 mmol, 0.1 equiv.). The vial was sealed with a microwave cap, evacuated and backfilled with nitrogen, followed by addition of anhydrous *N*,*N*-dimethylformamide (DMF) (1.0 mL) and trimethylamine (TEA) (1.0 mL) via syringe. The mixture was vacuum-purged and refilled with nitrogen for three cycles and then heated by microwave reactor at 80°C for 1 h (or at indicated temperature for indicated period of time). After completion of the reaction (monitored by TLC or LC-MS), the reaction mixture was filtered through a pad of celite, and the filtrate was extracted between ethyl acetate (EtOAc) and 1N hydrochloric acid (HCl) aqueous solution. The organic layer was washed with saturated aqueous sodium bicarbonate ($NaHCO_3$) and brine, dried over anhydrous sodium sulfate (Na_2SO_4), filtered, and concentrated under reduced pressure. The residue was purified by flash chromatography on silica with EtOAc/hexanes or CH_3OH/dichloromethane (CH_2Cl_2) gradient elution to afford the desired product.

(b) General procedure for amide coupling:

To a solution of carboxylic acid (0.1 mmol, 1.0 equiv.), substituted amine (0.12 mmol, 1.2 equiv.), and *O*-(7-azabenzotriazol-1-yl)-*N*,*N*,*N'*,*N'*-tetramethyluronium hexafluorophosphate (HATU) (0.12 mmol, 1.2 equiv.) in anhydrous DMF (0.5 mL) was added *N*,*N*-diisopropylethylamine (DIEA) (0.15 mmol, 1.5 equiv.; additional 1.5 equiv. per acid for the amine salt) under nitrogen. The reaction mixture was stirred at room temperature for 16 h. The mixture was quenched by the addition of water and extracted twice with EtOAc. The combined organic layers were washed with saturated aqueous $NaHCO_3$ and brine, dried over anhydrous Na_2SO_4, filtered, and evaporated under reduced pressure. The residue was purified by flash chromatography on silica (CH_3OH/CH_2Cl_2, 0:100 to 10:90) or reverse-phase column chromatography on C-18 (CH_3OH/water, 5:95 to 100:0) to afford the desired product.

(c) General procedure for hydrolysis without tetrahydropyranyl (THP)-protected indazole:

To a solution of methyl ester (0.5 mmol, 1.0 equiv.) in tetrahydrofuran (THF)/water (2.0 mL,

1:1, *v/v*) was added potassium hydroxide (KOH) (5.0 mmol, 10.0 equiv.), and the mixture was vigorously stirred at 40°C for 48 h or until the disappearance of the starting material (monitored by LC-MS). The reaction mixture was neutralized to pH 7 with 4N HCl aqueous solution and concentrated in vacuo. The crude carboxylic acid was directly used in the next step.

(d) General procedure for THP protection:

A mixture of indazole (5.0 mmol, 1.0 equiv.), 3,4-dihydro-2*H*-pyran (DHP) (7.5 mmol, 1.5 equiv.), and *p*-toluenesulfonic acid monohydrate (*p*-TsOH·H_2O) (1.0 mmol, 0.2 equiv.) in anhydrous CH_2Cl_2 (10.0 mL) was stirred under nitrogen at room temperature for 16 h. The mixture was quenched by the addition of saturated aqueous $NaHCO_3$ and extracted twice with EtOAc. The combined organic layers were washed with brine, dried over anhydrous Na_2SO_4, filtered, and concentrated. The residue was purified by flash chromatography on silica (EtOAc/hexanes, 0:100 to 40:60) to afford the desired product.

(e) General procedure for hydrolysis with THP-protected indazole:

To a solution of methyl ester (0.5 mmol, 1.0 equiv.) in THF/water (2.0 mL, 1:1, *v/v*) was added lithium hydroxide monohydrate (LiOH·H_2O) (1.0 mmol, 2.0 equiv.), and the mixture was vigorously stirred at room temperature for 1 h. After completion of the reaction (monitored by LC-MS), the reaction mixture was neutralized to pH 7 with 4N HCl aqueous solution and concentrated in vacuo. The residue was purified by reverse-phase column chromatography on C-18 (CH_3OH/water, 5:95 to 100:0) to afford the desired product.

(f) General procedure for removal of THP or *t*-butyloxycarbonyl (Boc)-protecting group:

A mixture of THP-protected indazole with or without Boc-protected indazole (0.1 mmol) and 4N HCl solution in 1,4-dioxane (0.5 mL) was stirred at room temperature for 1 h. After completion of the reaction (monitored by LC-MS), the resulting mixture was neutralized to pH 7 with 4M NaOH aqueous solution and concentrated in vacuo. The residue was purified by reverse-phase column chromatography on C-18 (CH_3OH/water, 5:95 to 100:0) to afford the desired product.

Preparation of CDD-2110, CDD-2211, and CDD-2212 (also see fig. S6)

1-(1-(3-([1,1'-Biphenyl]-2-ylethynyl)-1*H*-indazole-5-carbonyl)pyrrolidin-3-yl)-*N*-methylpiperidine-4-carboxamide (CDD-2110)

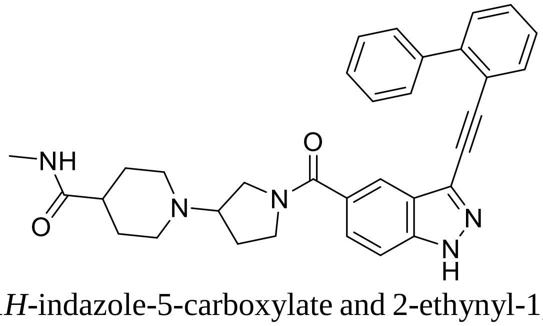

In step 1, methyl 3-bromo-1*H*-indazole-5-carboxylate and 2-ethynyl-1,1'-biphenyl were utilized in the general procedure for Sonogashira coupling to give methyl 3-([1,1'-biphenyl]-2-ylethynyl)-1*H*-indole-5-carboxylate (52%) as a pale yellow solid; ^{1}H NMR (600 MHz, CD_3OD) δ 8.19 (s, 1H), 8.02 (d, *J* = 8.8 Hz, 1H), 7.74 (d, *J* = 7.7 Hz, 1H), 7.69 (d, *J* = 7.6 Hz, 2H), 7.58–7.38 (m, 7H), 3.99 (s, 3H). In step 2, the cross-coupling intermediate was subjected to the general procedure for hydrolysis without THP-protected indazole to yield 3-([1,1'-biphenyl]-2-ylethynyl)-1*H*-

indazole-5-carboxylic acid. In step 3, the resulting acid and *N*-methyl-1-(pyrrolidin-3-yl)piperidine-4-carboxamide were utilized in the general procedure for amide coupling to afford the title compound (49% over two steps) as a light yellow oil; ^{1}H NMR (600 MHz, DMSO-d_6, mixture of diastereomers and rotamers) δ 7.78 (d, *J* = 7.6 Hz, 1H), 7.74–7.58 (m, 4H), 7.56–7.45 (m, 6H), 7.45–7.34 (m, 2H), 3.89–3.77 (m, 0.5H), 3.75–3.66 (m, 0.5H), 3.56–3.46 (m, 1H), 3.34–3.14 (m, 3H), 3.01–2.81 (m, 2H), 2.79–2.68 (m, 0.5H), 2.54 (dd, *J* = 27.7, 4.4 Hz, 3H), 2.23–2.14 (m, 0.5H), 2.12–1.72 (m, 4H), 1.71–1.44 (m, 4H); ^{13}C NMR (150 MHz, DMSO-d_6, mixture of diastereomers and rotamers) δ 174.8, 174.7, 168.4, 168.3, 143.4, 140.2, 140.0, 132.8, 130.4, 130.0, 129.7, 129.4, 129.1 (2 ×), 128.6, 128.3 (2 ×), 127.8, 127.7, 126.2, 126.1, 123.5, 120.2, 118.7, 110.8, 92.7, 84.0, 64.2. 62.9, 52.8, 51.7, 51.6, 50.8, 50.7, 49.9, 48.1, 44.9, 42.0, 41.9, 40.5, 29.9, 28.5, 28.1, 25.4; HRMS (ESI) *m/z* calcd for $C_{33}H_{34}N_5O_2$ [M + H]$^+$ 532.2713, found 532.2700.

(*R*)-(3-([1,1'-Biphenyl]-2-ylethynyl)-1*H*-indazol-5-yl)(3-(dimethylamino)pyrrolidin-1-yl)methanone (CDD-2211)

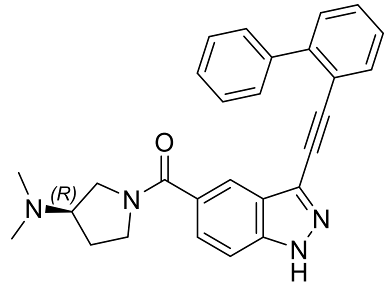

The same procedure for the synthesis of CDD-2110 was followed except that the amine was replaced with (*R*)-*N*,*N*-dimethyl-3-pyrrolidinamine in step 3 to afford the title compound (77% over two steps; steps 2 and 3) as a colorless oil; ^{1}H NMR (600 MHz, DMSO-d_6, two rotamers) δ 7.78 (dd, *J* = 7.7, 1.4 Hz, 1H), 7.68 (d, *J* = 7.6 Hz, 2H), 7.60 (d, *J* = 8.6 Hz, 1H), 7.55–7.44 (m, 6H), 7.43–7.37 (m, 2H), 3.81–3.77 (m, 0.5H), 3.73–3.67 (m, 0.5H), 3.56–3.50 (m, 0.5H), 3.43–3.28 (m, 2H), 3.21–3.12 (m, 0.5H), 2.81–2.68 (m, 0.5H), 2.68–2.58 (m, 0.5H), 2.22 (s, 3H), 2.14–1.98 (m, 4H), 1.85–1.75 (m, 0.5H), 1.75–1.63 (m, 0.5H); ^{13}C NMR (150 MHz, DMSO-d_6, two rotamers) δ 168.4, 143.2, 140.6, 140.0, 132.7, 130.1, 129.6, 129.3, 129.0 (2 ×), 128.4, 128.2 (2 ×), 127.6 (2 ×), 125.9, 125.7, 123.6, 120.3, 118.6, 111.0, 92.6, 84.3, 65.1, 63.9, 53.0, 50.2, 48.2, 45.0, 43.9, 43.6, 30.5, 28.4; HRMS (ESI) *m/z* calcd for $C_{28}H_{27}N_4O$ [M + H]$^+$ 435.2185, found 435.2174.

(*S*)-(3-([1,1'-Biphenyl]-2-ylethynyl)-1*H*-indazol-5-yl)(3-(dimethylamino)pyrrolidin-1-yl)methanone (CDD-2212)

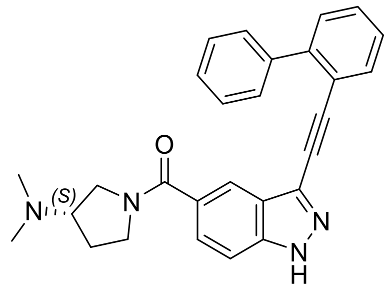

The same procedure for the synthesis of CDD-2110 was followed except that the amine was replaced with (*S*)-*N*,*N*-dimethyl-3-pyrrolidinamine in step 3 to afford the title compound (19% over two steps; steps 2 and 3) as a colorless oil; ^{1}H NMR (600 MHz, CD_3OD, two rotamers) δ 7.73 (dd, *J* = 7.7, 1.4 Hz, 1H), 7.66 (d, *J* = 7.0 Hz, 2H), 7.59 (dd, *J* = 8.6, 6.0 Hz, 1H), 7.54 (dd, *J* = 8.6,

1.5 Hz, 1H), 7.51–7.44 (m, 4H), 7.44–7.38 (m, 2H), 7.30 (d, *J* = 14.4 Hz, 1H), 3.98 (dd, *J* = 12.1, 7.3 Hz, 0.5H), 3.94–3.85 (m, 0.5H), 3.76–3.64 (m, 0.5H), 3.56–3.36 (m, 2H), 3.26–3.20 (m, 0.5H), 2.99–2.92 (m, 0.5H), 2.84–2.77 (m, 0.5H), 2.38 (s, 3H), 2.35–2.12 (m, 4H), 2.00–1.91 (m, 0.5H), 1.87–1.77 (m, 0.5H); ^{13}C NMR (150 MHz, DMSO-d_6, two rotamers) δ 168.3, 143.3, 140.3, 140.0, 132.8, 130.3, 129.9, 129.6, 129.4, 129.1 (2 ×), 128.6, 128.2 (2 ×), 127.7, 127.7, 126.2, 126.0, 123.5, 120.2, 118.7, 110.9, 92.7, 84.0, 65.2, 64.0, 53.0, 50.3, 48.2, 45.0, 43.9, 43.7, 30.5, 28.5; HRMS (ESI) *m/z* calcd for $C_{28}H_{27}N_4O$ [M + H]$^+$ 435.2185, found 435.2172.

*Preparation of compounds **S1** and **S2** (also see fig. S7)*

(*R*)-(3-([1,1'-Biphenyl]-2-ylethynyl)-1*H*-indazol-5-yl)(3-aminopyrrolidin-1-yl)methanone (**S1**, CDD-2579)

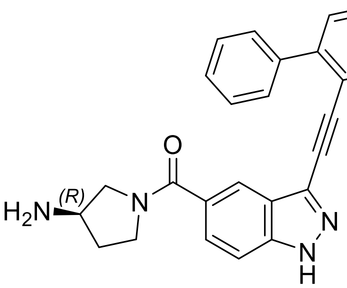

In step 1, 3-iodo-1*H*-indazole-5-carboxylic acid and *tert*-butyl (*R*)-pyrrolidin-3-ylcarbamate were utilized in the general procedure for amide coupling. In step 2, the general procedure for Sonogashira coupling was employed using 2-ethynyl-1,1'-biphenyl. In step 3, the cross-coupling intermediate was subjected to the general procedure for removal of Boc-protecting group to afford the title compound (52% over three steps) as a white foam; ^{1}H NMR (600 MHz, DMSO-d_6, two rotamers) 8.27 (br s, 1H), 8.11 (br s, 1H), 7.78 (d, *J* = 7.7 Hz, 1H), 7.67 (d, *J* = 7.5 Hz, 2H), 7.64 (d, *J* = 8.6 Hz, 1H), 7.57–7.53 (m, 2H), 7.51–7.38 (m, 6H), 3.97–3.75 (m, 2H), 3.72–3.60 (m, 2H), 3.42-3.28 (m, 1H), 2.38–2.15 (m, 1H), 2.13–1.91 (m, 1H); ^{13}C NMR (150 MHz, DMSO-d_6, two rotamers) δ 168.7, 158.5, 143.5, 140.3, 140.0, 133.0, 129.8, 129.7, 129.2 (2 ×), 128.8, 128.4 (2 ×), 127.9, 127.8, 126.3, 123.5, 120.2, 119.0, 111.1, 92.8, 83.9, 52.4, 50.0, 49.7, 48.7, 46.8, 44.0, 30.2, 28.2; HRMS (ESI) *m/z* calcd for $C_{26}H_{23}N_4O$ [M + H]$^+$ 407.1872, found 407.1861.

(*R*)-(3-([1,1'-Biphenyl]-2-ylethynyl)-1*H*-indazol-5-yl)(3-hydroxypyrrolidin-1-yl)methanone (**S2**, CDD-2594)

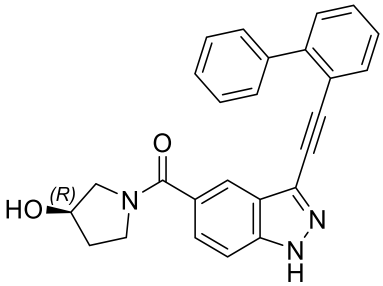

The same procedure for the synthesis of **S1** (CDD-2579) was followed except that the amine was replaced with (*R*)-pyrrolidin-3-ol in step 1 to afford the title compound (43% over three steps) as a white foam; ^{1}H NMR (600 MHz, DMSO-d_6, two rotamers) δ 7.79–7.75 (m, 1H), 7.65 (t, *J* = 7.3 Hz, 2H), 7.62–7.59 (m, 2H), 7.56–7.52 (m, 2H), 7.50–7.45 (m, 4H), 7.44–7.38 (m, 1H), 7.36 (d, *J* = 3.7 Hz, 1H), 4.40 (br s, 0.5H), 4.23 (br s, 0.5H), 3.68–3.62 (m, 2H), 3.43–3.41 (m, 1H), 3.29–

3.23 (m, 0.5H), 3.11–3.07 (m, 0.5H), 2.04–1.98 (m, 0.5H), 1.94–1.83 (m, 1H), 1.84–1.75 (m, 0.5H); ^{13}C NMR (150 MHz, DMSO-*d*$_6$, two rotamers) δ 169.0, 143.9, 143.9, 140.5, 140.4, 133.2, 133.1, 132.5, 131.9, 131.9, 130.9, 130.1, 129.9, 129.5, 129.4, 129.2, 129.2, 128.7, 128.2, 128.2, 128.1, 126.7, 123.9, 120.5, 119.1, 118.9, 111.2, 93.3, 84.3, 69.8, 68.6, 57.7, 54.9, 47.6, 44.6, 34.8, 32.7; HRMS (ESI) *m/z* calcd for $C_{26}H_{22}N_3O_2$ $[M + H]^+$ 408.1712, found 408.1700.

Preparation of CDD-2807, CDD-3348, and compounds ***S3*–*S7*** *(also see fig. S8)*

(3-([1,1'-Biphenyl]-2-ylethynyl)-1*H*-indazol-5-yl)(2,6-diazaspiro[3.5]nonan-2-yl)methanone (CDD-2807)

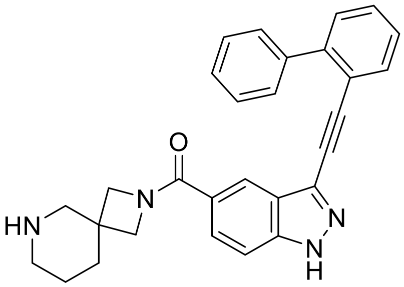

In step 1, methyl 3-iodo-1*H*-indazole-5-carboxylate was subject to the general procedure for THP protection to yield methyl 3-iodo-1-(tetrahydro-2H-pyran-2-yl)-1*H*-indazole-5-carboxylate. In step 2, the THP-protected indazole and 2-ethynyl-1,1'-biphenyl were utilized in the general procedure for Sonogashira coupling. In step 3, the general procedure for hydrolysis with THP-protected indazole was applied to yield 3-([1,1'-biphenyl]-2-ylethynyl)-1-(tetrahydro-2*H*-pyran-3-yl)-1*H*-indazole-5-carboxylic acid (75% over three steps) as a pale yellow solid; ^{1}H NMR (600 MHz, DMSO-*d*$_6$) δ 13.06 (s, 1H), 8.15 (s, 1H), 8.01 (dd, *J* = 8.8, 1.5 Hz, 1H), 7.87 (d, *J* = 8.8 Hz, 1H), 7.81 (dd, J = 7.7, 1.3 Hz, 1H), 7.76–7.65 (m, 2H), 7.60–7.51 (m, 4H), 7.48 (td, *J* = 7.5, 1.5 Hz, 1H), 7.46–7.40 (m, 1H), 5.93 (dd, *J* = 9.5, 2.5 Hz, 1H), 3.91–3.83 (m, 1H), 3.80–3.70 (m, 1H), 2.40–2.27 (m, 1H), 2.08–1.89 (m, 2H), 1.80–1.66 (m, 1H), 1.65–1.52 (m, 2H). In step 4, the acid and *tert*-butyl 2,6-diazaspiro[3.5]nonane-6-carboxylate were utilized in the general procedure for amide coupling. In step 5, the general procedure for removal of THP and Boc-protecting group was employed to afford the title compound (31% over two steps) as a white solid; ^{1}H NMR (600 MHz, DMSO-*d*$_6$) δ 7.80 (dd, *J* = 7.7, 1.3 Hz, 1H), 7.72 (dd, *J* = 8.2, 1.3 Hz, 2H), 7.69–7.64 (m, 2H), 7.61 (dd, *J* = 8.6, 0.9 Hz, 1H), 7.58–7.50 (m, 4H), 7.48 (td, *J* = 7.4, 1.6 Hz, 1H), 7.46–7.42 (m, 1H), 3.84 (d, *J* = 8.4 Hz, 1H), 3.81–3.74 (m, 2H), 3.70 (d, *J* = 9.7 Hz, 1H), 2.79–2.65 (m, 2H), 2.59–2.51 (m, 2H), 1.68 (br s, 2H), 1.46–1.23 (m, 2H); ^{13}C NMR (150 MHz, DMSO-*d*$_6$) δ 169.4, 143.1, 141.3, 139.8, 132.9, 129.7, 129.4, 129.0 (2 ×), 128.6, 128.3 (2 ×), 127.8, 127.6, 126.5, 126.0, 123.7, 120.2, 119.6, 111.2, 92.5, 84.4, 62.2, 57.7, 54.6, 45.3, 34.7, 34.1, 23.2; HRMS (ESI) *m/z* calcd for $C_{29}H_{27}N_4O$ $[M + H]^+$ 447.2185, found 447.2173.

3-(1-(1-(1-(3-([1,1'-Biphenyl]-2-ylethynyl)-1*H*-indazole-5-carbonyl)pyrrolidin-3-yl)piperidine-4-carbonyl)piperidin-4-yl)-*N*-methylpropanamide (CDD-3348)

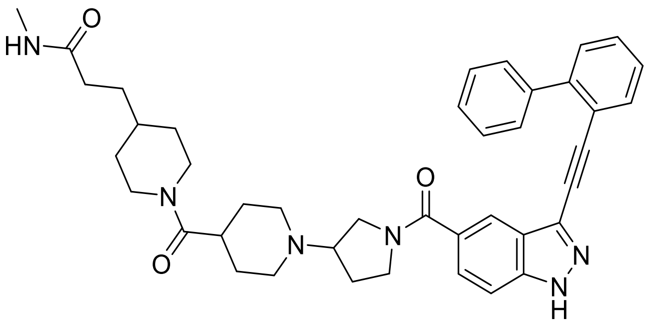

The same procedure for the synthesis of CDD-2807 was followed except that the amine was replaced with *N*-methyl-3-(1-(1-(pyrrolidin-3-yl)piperidine-4-carbonyl)piperidin-4-yl)propanamide, derived from *N*-methyl-3-(piperidin-4-yl)propenamide and 1-(1-(*tert*-butoxycarbonyl)pyrrolidin-3-yl)piperidine-4-carboxylic acid via amide coupling and subsequent removal of Boc-protecting group, in step 4 to afford the title compound (71% over two steps; steps 4 and 5) as a pale yellow solid; ^{1}H NMR (800 MHz, CD_3OD, mixture of diastereomers and rotamers) δ 7.73 (d, *J* = 7.6 Hz, 1H), 7.65 (d, *J* = 7.5 Hz, 2H), 7.59 (t, *J* = 8.2 Hz, 1H), 7.53 (t, *J* = 7.7 Hz, 1H), 7.50–7.45 (m, 4H), 7.42 (t, *J* = 7.3 Hz, 2H), 7.30 (d, *J* = 7.4 Hz, 1H), 4.54–4.43 (m, 1H), 4.08–3.96 (m, 1H), 3.96–3.84 (m, 1H), 3.70–3.33 (m, 3H), 3.28–2.85 (m, 4H), 2.77–2.49 (m, 5H), 2.38–2.27 (m, 1H), 2.26–2.14 (m, 3H), 2.13–2.02 (m, 1H), 1.99–1.45 (m, 10H), 1.19–0.93 (m, 2H); ^{13}C NMR (200 MHz, CD_3OD, mixture of diastereomers and rotamers) δ 176.8, 176.7, 175.3, 172.1, 171.9, 145.9, 145.8, 145.7, 142.4, 142.4, 142.1, 141.8, 134.5, 134.0, 131.5, 131.2, 130.9, 130.9, 130.8, 130.6, 130.6, 130.5, 130.4, 129.6, 129.6, 129.6, 129.5, 129.2, 129.0, 128.9, 128.7, 128.5, 127.6, 127.5, 125.5, 125.3, 125.2, 122.2, 122.2, 120.9, 120.8, 112.1, 95.0, 84.4, 66.0, 64.9, 54.4, 54.4, 53.3, 53.0, 52.5, 52.2, 51.3, 50.0, 47.0, 46.9, 46.6, 43.5, 43.4, 39.4, 39.3, 37.1, 37.0, 34.3, 34.3, 34.2, 33.5, 33.5, 33.0, 33.0, 31.3, 29.7, 29.6, 29.5, 29.4, 26.5, 26.5; HRMS (ESI) *m/z* calcd for $C_{41}H_{47}N_6O_3$ $[M + H]^+$ 671.3710, found 671.3694.

(3-([1,1'-Biphenyl]-2-ylethynyl)-1*H*-indazol-5-yl)(2,6-diazaspiro[4.5]decan-2-yl)methanone (**S3**, CDD-2765)

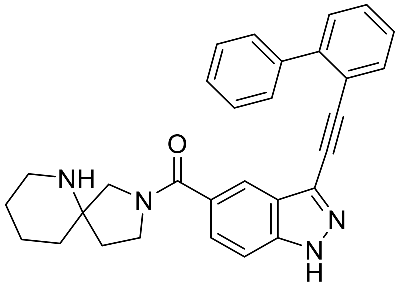

The same procedure for the synthesis of CDD-2807 was followed except that the amine was replaced with *tert*-butyl 2,6-diazaspiro[4.5]decane-6-carboxylate hydrochloride in step 4 to afford the title compound (77% over two steps; steps 4 and 5) as a light yellow oil; ^{1}H NMR (600 MHz, DMSO-d_6) δ 7.76 (d, *J* = 7.6 Hz, 1H), 7.68 (d, *J* = 7.5 Hz, 2H), 7.57 (t, *J* = 7.0 Hz, 1H), 7.54–7.36 (m, 8H), 3.67–3.51 (m, 2H), 3.22–3.13 (m, 2H), 2.75 (s, 1H), 2.63 (s, 1H), 2.01–1.85 (m, 1H), 1.82–1.67 (m, 1H), 1.62–1.55 (m, 1H), 1.53–1.47 (m, 1H), 1.45–1.17 (m, 4H); HRMS (ESI) *m/z* calcd for $C_{30}H_{29}N_4O$ $[M + H]^+$ 461.2341, found 461.2328.

(3-([1,1'-Biphenyl]-2-ylethynyl)-1*H*-indazol-5-yl)(2,7-diazaspiro[4.5]decan-2-yl)methanone (**S4**, CDD-2766)

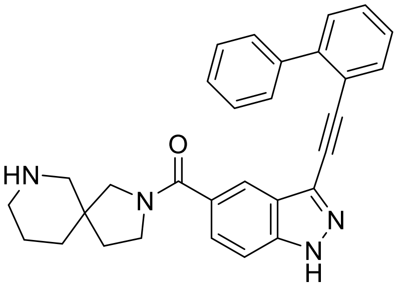

The same procedure for the synthesis of CDD-2807 was followed except that the amine was replaced with *tert*-butyl 2,7-diazaspiro[4.5]decane-7-carboxylate hydrochloride in step 4 to afford the title compound (51% over two steps; steps 4 and 5) as a light yellow oil; ^{1}H NMR (600 MHz, DMSO-d_6) δ 7.84–7.64 (m, 3H), 7.57 (d, J = 8.5 Hz, 1H), 7.53–7.33 (m, 8H), 3.63–3.55 (m, 2H), 3.11–2.96 (m, 2H), 2.46–2.33 (m, 2H), 2.04–1.85 (m, 1H), 1.83–1.68 (m, 1H), 1.59 (s, 1H), 1.52–1.29 (m, 4H), 1.15 (s, 1H); HRMS (ESI) *m/z* calcd for $C_{30}H_{29}N_4O$ [M + H]$^+$ 461.2341, found 461.2329.

(3-([1,1'-Biphenyl]-2-ylethynyl)-1*H*-indazol-5-yl)(2,8-diazaspiro[4.5]decan-2-yl)methanone (**S5**, CDD-2674)

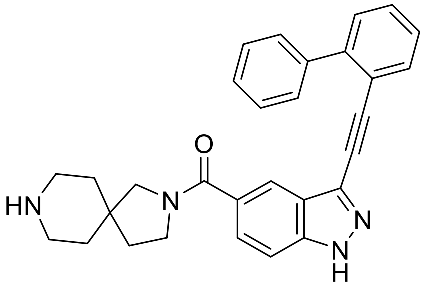

The same procedure for the synthesis of CDD-2807 was followed except that the amine was replaced with *tert*-butyl 2,8-diazaspiro[4.5]decane-8-carboxylate in step 4 to afford the title compound (82% over two steps; steps 4 and 5) as a colorless oil; ^{1}H NMR (600 MHz, DMSO-d_6) δ 7.81–7.76 (m, 1H), 7.71–7.66 (m, 2H), 7.63–7.57 (m, 1H), 7.57–7.32 (m, 8H), 3.65–3.59 (m, 2H), 3.09 (s, 2H), 2.79–2.65 (m, 2H), 2.61 (s, 1H), 2.45 (s, 1H), 1.87–1.78 (m, 1H), 1.78–1.65 (m, 1H), 1.60–1.42 (m, 2H), 1.39–1.22 (m, 2H); HRMS (ESI) *m/z* calcd for $C_{30}H_{29}N_4O$ [M + H]$^+$ 461.2341, found 461.2327.

(3-([1,1'-Biphenyl]-2-ylethynyl)-1*H*-indazol-5-yl)(2,5-diazaspiro[3.5]nonan-2-yl)methanone (**S6**, CDD-2808)

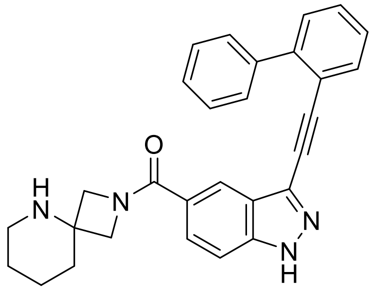

The same procedure for the synthesis of CDD-2807 was followed except that the amine was replaced with *tert*-butyl 2,5-diazaspiro[3.5]nonane-5-carboxylate oxalate in step 4 to afford the title compound (85% over two steps; steps 4 and 5) as a white solid; ^{1}H NMR (600 MHz, DMSO-

d_6) δ 7.79 (d, *J* = 7.6 Hz, 1H), 7.74–7.69 (m, 3H), 7.67 (d, *J* = 8.8 Hz, 1H), 7.62 (d, *J* = 8.6 Hz, 1H), 7.58–7.50 (m, 4H), 7.50–7.42 (m, 2H), 3.98–3.92 (m, 1H), 3.92–3.84 (m, 2H), 3.82–3.77 (m, 1H), 2.68–2.62 (m, 1H), 2.61–2.54 (m, 1H), 1.67–1.58 (m, 2H), 1.55–1.48 (m, 1H), 1.48–1.40 (m, 1H), 1.38–1.31 (m, 2H); ^{13}C NMR (150 MHz, DMSO-d_6) δ 169.4, 143.3, 140.7, 139.8, 133.1, 129.7, 129.6, 129.0 (2 ×), 128.9, 128.3 (2 ×), 127.9, 127.7, 127.0, 126.5, 123.6, 120.1, 119.8, 110.9, 92.7, 83.9, 64.3, 60.1, 53.3, 42.3, 34.5, 24.9, 21.3; HRMS (ESI) *m/z* calcd for $C_{29}H_{27}N_4O$ $[M + H]^+$ 447.2185, found 447.2171.

(3-([1,1'-Biphenyl]-2-ylethynyl)-1*H*-indazol-5-yl)(2,7-diazaspiro[3.5]nonan-2-yl)methanone (**S7**, CDD-2806)

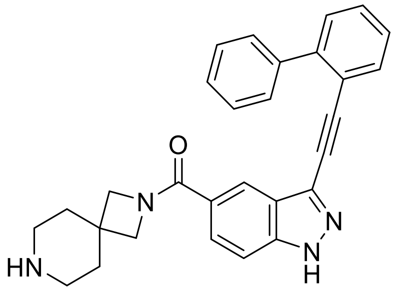

The same procedure for the synthesis of CDD-2807 was followed except that the amine was replaced with *tert*-butyl 2,7-diazaspiro[3.5]nonane-7-carboxylatein in step 4 to afford the title compound (26% over two steps; steps 4 and 5) as a colorless oil; ^{1}H NMR (600 MHz, DMSO-d_6) δ 7.79 (d, *J* = 7.7 Hz, 1H), 7.72 (d, *J* = 7.6 Hz, 2H), 7.69 (s, 1H), 7.66 (d, *J* = 8.2 Hz, 1H), 7.61 (d, *J* = 8.7 Hz, 1H), 7.58–7.50 (m, 4H), 7.48 (td, *J* = 7.4, 1.7 Hz, 1H), 7.43 (t, *J* = 7.4 Hz, 1H), 3.85 (s, 2H), 3.79 (s, 2H), 2.68–2.52 (m, 4H), 1.62 (t, *J* = 5.4 Hz, 4H); ^{13}C NMR (150 MHz, DMSO-d_6) δ 169.2, 143.2, 140.8, 139.8, 133.0, 129.7, 129.5, 129.0 (2 ×), 128.8, 128.3 (2 ×), 127.8, 127.7, 126.7, 126.4, 123.6, 120.1, 119.7, 110.9, 92.6, 84.0, 63.4, 59.0, 42.8 (2 ×), 36.1 (2 ×), 34.3; HRMS (ESI) *m/z* calcd for $C_{29}H_{27}N_4O$ $[M + H]^+$ 447.2185, found 447.2173.

Preparation of compounds ***S8*****–*****S10*** *(also see fig. S7)*

(*R*)-(3-([1,1'-Biphenyl]-2-ylethynyl)-1-methyl-1*H*-indazol-5-yl)(3-dimethylamino)pyrrolidin-1-yl)methanone (**S8**, CDD-2533)

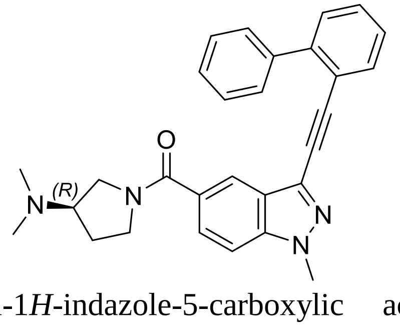

In step 1, 3-iodo-1-methyl-1*H*-indazole-5-carboxylic acid and (*R*)-*N*,*N*-dimethyl-3-pyrrolidinamine were utilized in the general procedure for amide coupling. In step 2, the general procedure for Sonogashira coupling was employed using 2-ethynyl-1,1'-biphenyl to afford the title compound (16% over two steps) as a light yellow oil; ^{1}H NMR (600 MHz, DMSO-d_6, two rotamers) δ 7.77 (dd, *J* = 7.7, 1.4 Hz, 1H), 7.72 (dd, *J* = 8.6, 2.9 Hz, 1H), 7.66 (d, *J* = 7.5 Hz, 2H), 7.59–7.51 (m, 2H), 7.51–7.44 (m, 4H), 7.43–7.37 (m, 2H), 4.07 (s, 3H), 3.84–3.76 (m, 0.5H), 3.75–3.65 (m, 0.5H), 3.59–3.48 (m, 0.5H), 3.36–3.27 (m, 2H), 3.22–3.12 (m, 0.5H), 2.80–2.69 (m,

0.5H), 2.67–2.59 (m, 0.5H), 2.21 (s, 3H), 2.16–1.96 (m, 4H), 1.84–1.78 (m, 0.5H), 1.74–1.64 (m, 0.5H); ^{13}C NMR (150 MHz, DMSO-d_6, two rotamers) δ 168.3, 168.2, 143.3, 140.0, 139.9, 132.8, 130.5, 130.1, 129.6, 129.4, 129.1, 129.0, 128.3, 127.7, 127.7, 127.4, 126.2, 126.0, 124.2, 124.1, 120.1, 118.8, 118.8, 110.4, 93.1, 83.5, 65.2, 63.9, 53.0, 50.3, 48.2, 45.0, 43.9, 43.7, 36.0, 30.5, 28.5; HRMS (ESI) *m/z* calcd for $C_{29}H_{29}N_4O$ $[M + H]^+$ 449.2341, found 449.2328.

(*R*)-(3-([1,1'-Biphenyl]-3-ylethynyl)-1*H*-indazol-5-yl)(3-(dimethylamino)pyrrolidin-1-yl)methanone (**S9**, CDD-2532)

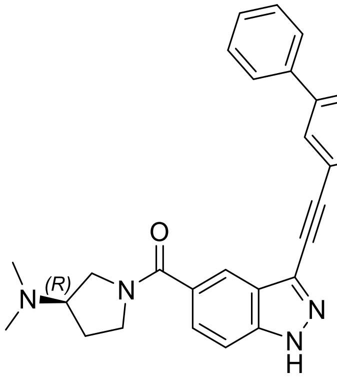

In step 1, 3-iodo-1*H*-indazole-5-carboxylic acid and (*R*)-*N*,*N*-dimethyl-3-pyrrolidinamine were utilized in the general procedure for amide coupling. In step 2, the general procedure for Sonogashira coupling was employed using 3-ethynyl-1,1'-biphenyl to afford the title compound (26% over two steps) as a light yellow oil; ^{1}H NMR (600 MHz, DMSO-d_6, two rotamers) δ 8.06 (d, *J* = 4.8 Hz, 1H), 7.97 (s, 1H), 7.77–7.72 (m, 3H), 7.71–7.64 (m, 2H), 7.61–7.54 (m, 2H), 7.49 (t, *J* = 7.7 Hz, 2H), 7.40 (t, *J* = 7.2 Hz, 1H), 3.76–3.71 (m, 0.5H), 3.66–3.62 (m, 0.5H), 3.58–3.51 (m, 0.5H), 3.51–3.44 (m, 0.5H), 3.36–3.34 (m, 1H), 3.27–3.22 (m, 1H), 2.75–2.66 (m, 0.5H), 2.66–2.58 (m, 0.5H), 2.17 (s, 3H), 2.10–1.95 (m, 4H), 1.81–1.72 (m, 0.5H), 1.72–1.62 (m, 0.5H); ^{13}C NMR (150 MHz, DMSO-d_6, two rotamers) δ 168.4, 140.8, 140.3, 139.1, 130.5, 130.2, 129.6, 129.5, 129.0 (2 ×), 128.6, 128.3, 127.9, 127.5, 126.9 (2 ×), 126.3, 126.2, 123.5, 122.5, 119.0, 110.8, 92.8, 81.4, 65.1, 63.9, 53.0, 50.3, 48.2, 45.1, 43.8, 43.6, 30.4, 28.3; HRMS (ESI) *m/z* calcd for $C_{28}H_{27}N_4O$ $[M + H]^+$ 435.2185, found 435.2172.

(*R*)-(3-([1,1'-Biphenyl]-4-ylethynyl)-1*H*-indazol-5-yl)(3-(dimethylamino)pyrrolidin-1-yl)methanone (**S10**, CDD-2563)

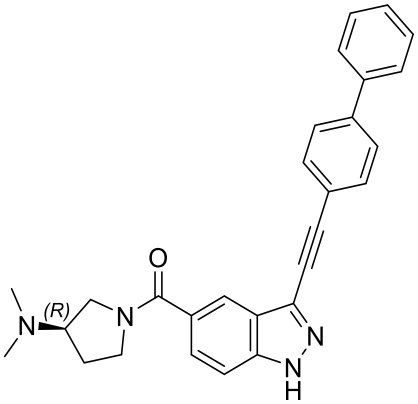

The same procedure for the synthesis of **S9** (CDD-2532) was followed except that the alkyne was replaced with 4-ethynyl-1,1'-biphenyl in step 2 to afford the title compound (8% over two steps)

as a light yellow oil; ^{1}H NMR (600 MHz, DMSO-d_6, two rotamers) δ 8.03 (s, 1H), 7.78 (s, 4H), 7.74 (d, J = 7.2 Hz, 2H), 7.66 (d, J = 8.6 Hz, 1H), 7.59 (t, J = 8.8 Hz, 1H), 7.50 (t, J = 7.6 Hz, 2H), 7.41 (t, J = 7.3 Hz, 1H), 3.80–3.71 (m, 0.5H), 3.69–3.61 (m, 0.5H), 3.60–3.46 (m, 2H), 3.29–3.22 (m, 1H), 2.79–2.69 (m, 0.5H), 2.69–2.60 (m, 0.5H), 2.19 (s, 3H), 2.13–1.96 (m, 4H), 1.83–1.63 (m, 1H); ^{13}C NMR (150 MHz, DMSO-d_6, two rotamers) δ 168.3, 140.6, 140.3, 139.1, 132.1 (2 ×), 130.5, 130.1, 129.0 (2 ×), 128.6, 128.0, 126.9 (2 ×), 126.7 (2 ×), 126.3, 123.5, 120.8, 118.9, 110.8, 92.8, 81.8, 65.2, 63.9, 53.1, 50.3, 48.2, 45.0, 43.9, 43.6, 30.4, 28.3; HRMS (ESI) *m/z* calcd for $C_{28}H_{27}N_4O$ [M + H]$^+$ 435.2185, found 435.2173.

*Preparation of compounds **S11** and **S12** (also see fig. S9)*

2-([1,1'-Biphenyl]-2-ylmethyl)-4,4,5,5-tetramethyl-1,3,2-dioxaborolane

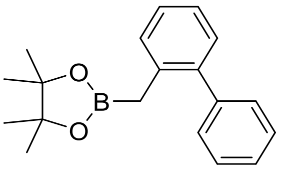

To a solution of bis(pinacolato)diborane (1.3 mmol, 1.3 equiv.) and 2-(bromomethyl)-1,1'-biphenyl (1.0 mmol, 1.0 equiv.) in anhydrous THF (2.0 mL) were added 4,5-bis(diphenylphosphino)-9,9-dimethylxanthene (Xantphos) (0.05 mmol, 0.05 equiv.) and copper(I) chloride (CuCl) (0.05 mmol, 0.05 equiv.) under nitrogen. The reaction mixture was stirred at room temperature for 16 h and filtered through a pad of celite. The filtrate was concentrated and purified by flash chromatography to afford 2-([1,1'-biphenyl]-2-ylmethyl)-4,4,5,5-tetramethyl-1,3,2-dioxaborolane (70%) as a white solid; ^{1}H NMR (600 MHz, $CDCl_3$) δ 7.42 (m, 4H), 7.35 (m, 1H), 7.31 (m, 2H), 7.26 (m, 2H), 2.34 (s, 2H), 1.20 (s, 12H).

Potassium ([1,1'-biphenyl]-2-ylmethyl)trifluoroborate

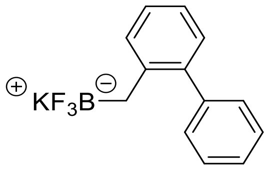

To a solution of 2-([1,1'-Biphenyl]-2-ylmethyl)-4,4,5,5-tetramethyl-1,3,2-dioxaborolane (0.3 mmol) in CH_3OH (2.0 mL) was added potassium bifluoride (KHF_2) (0.2 mL, 4.5 M in water) dropwise. The solution was stirred at room temperature for 1 h and concentrated to yield potassium ([1,1'-biphenyl]-2-ylmethyl)trifluoroborate. This product was subsequently used in the next step without undergoing any purification process.

(*R*)-(3-([1,1'-Biphenyl]-2-ylmethyl)-1*H*-indazol-5-yl)(3-(dimethylamino)pyrrolidin-1-yl)methanone (**S11**, CDD-2647)

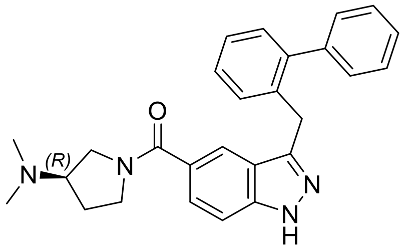

In step 1, 3-iodo-1-(tetrahydro-2*H*-pyran-2-yl)-1*H*-indazole-5-carboxylic acid and (*R*)-*N*,*N*-dimethyl-3-pyrrolidinamine were utilized in the general procedure for amide coupling. In step 2, an oven-dried microwave vial equipped with magnetic stir bar was charged with the amide (0.1 mmol, 1.0 equiv.), potassium ([1,1'-biphenyl]-2-ylmethyl)trifluoroborate (0.25 mmol, 2.5 equiv.), palladium(II) acetate ($Pd(OAc)_2$) (0.01 mmol, 0.1 equiv.), 2-dicyclohexylphosphino-2’,6’-diisopropoxybiphenyl (RuPhos) (0.02 mmol, 0.2 equiv.), and cesium carbonate (Cs_2CO_3) (0.3 mmol, 3.0 equiv.). The vial was sealed with a microwave cap, evacuated and backfilled with nitrogen, followed by addition of anhydrous toluene (3.0 mL) and water (0.3 mL, purged with nitrogen) via syringe. The mixture was vacuum-purged and refilled with nitrogen for three cycles and then heated by microwave reactor at 120°C for 1 h. After completion of the reaction (monitored by LC-MS), the reaction mixture was filtered through a pad of celite. The filtrate was concentrated and partially purified by flash chromatography. In step 3, the general procedure for removal of THP was applied to afford the title compound (29% over three steps) as a yellow oil; ^{1}H NMR (600 MHz, $CDCl_3$, two rotamers) δ 7.52 (dd, *J* = 8.6, 1.5 Hz, 1H), 7.38–7.26 (m, 10H), 7.21–7.13 (m, 1H), 4.37–4.26 (m, 2H), 4.00–3.90 (m, 0.5H), 3.87–3.76 (m, 0.5H), 3.70–3.59 (m, 0.5H), 3.53–3.37 (m, 1.5H), 3.34–3.14 (m, 1H), 2.85–2.72 (m, 0.5H), 2.71–2.56 (m, 0.5H), 2.34 (s, 3H), 2.26–1.98 (m, 4H), 1.90–1.64 (m, 1H); HRMS (ESI) *m/z* calcd for $C_{27}H_{29}N_4O$ $[M + H]^+$ 425.2341, found 425.2331.

(*R*)-(3-([1,1'-Biphenyl]-2-yl)-1*H*-indazol-5-yl)(3-(dimethylamino)pyrrolidin-1-yl)methanone (**S12**, CDD-2573)

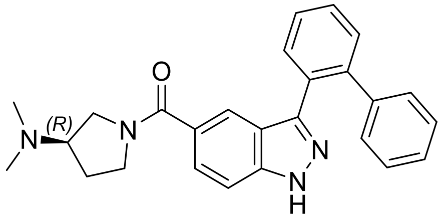

In step 1, 3-iodo-1-(tetrahydro-2*H*-pyran-2-yl)-1*H*-indazole-5-carboxylic acid and (*R*)-*N*,*N*-dimethyl-3-pyrrolidinamine were utilized in the general procedure for amide coupling. In step 2, an oven-dried microwave vial equipped with magnetic stir bar was charged with the amide (0.05 mmol, 1.0 equiv.), [1,1'-biphenyl]-2-ylboronic acid (0.1 mmol, 2.0 equiv.), tetrakis(triphenylphosphine)palladium(0) ($Pd(PPh_3)_4$) (0.5 mmol%, 0.1 equiv.), and potassium carbonate (K_2CO_3) (0.1 mmol, 2.0 equiv.). The vial was sealed with a microwave cap, evacuated and backfilled with nitrogen, followed by addition of anhydrous DMF (0.3 mL) and water (0.1 mL, purged with nitrogen) via syringe. The mixture was vacuum-purged and refilled with nitrogen for three cycles and then heated by microwave reactor at 100°C for 1 h. After completion of the reaction (monitored by LC-MS), the reaction mixture was filtered through a pad of celite. The filtrate was concentrated and partially purified by flash chromatography. In step 3, the cross-coupling intermediate was subjected to the general procedure for removal of THP to afford the title compound (45% over three steps) as a yellow oil; ^{1}H NMR (600 MHz, $CDCl_3$, two rotamers) δ 7.65 (d, *J* = 7.5 Hz, 1H), 7.54 (d, *J* = 6.1 Hz, 2H), 7.50–7.32 (m, 4H), 7.21 (br s, 2H), 7.12 (d, *J* = 7.4 Hz, 3H), 3.94–3.84 (m, 0.5H), 3.84–3.72 (m, 0.5H), 3.69–3.54 (m, 0.5H), 3.46–3.34 (m, 1H), 3.26–3.11 (m, 1.5H), 2.85–2.58 (m, 1H), 2.33 (s, 3H), 2.25–1.99 (m, 4H), 1.88–1.54 (m, 1H); HRMS (ESI) *m/z* calcd for $C_{26}H_{27}N_4O$ $[M + H]^+$ 411.2185, found 411.2176.

Supplementary figures

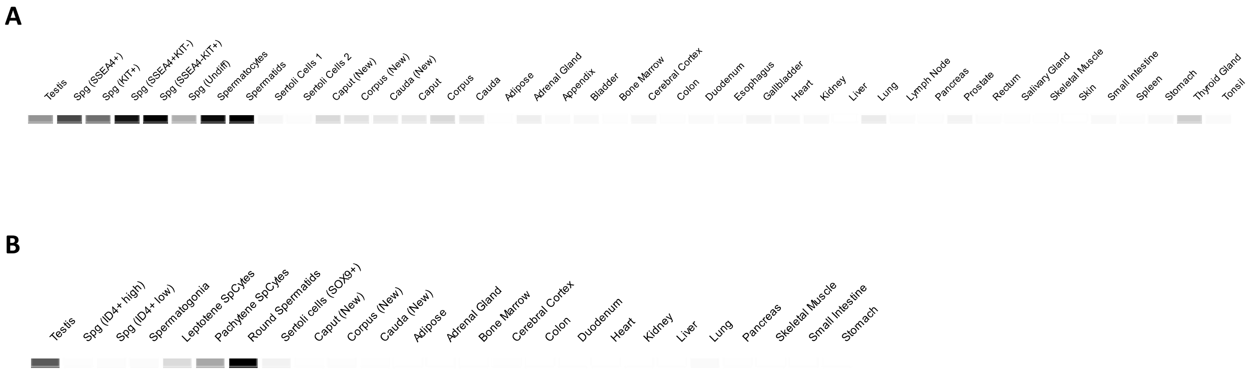

fig. S1. STK33 expression in various tissue types. The Mammalian Reproductive Genetics Database V2 (*13*, *28*) was used to search tissue expression of STK33 in both humans and mice. In humans (**A**), *STK33* has high expression in reproductive tissues especially spermatids. In the mouse (**B**), *Stk33* expression was found to be highly expressed in round spermatids. Expression data compiled in this database was collected from dPCR (RNA-seq) data. Extragonadal expression of *STK33* in human thyroid gland, lung, adrenal gland, etc. indicates that an STK33 clinical candidate inhibitor will require a battery of safety and toxicological studies, including thyroid hormone analysis.

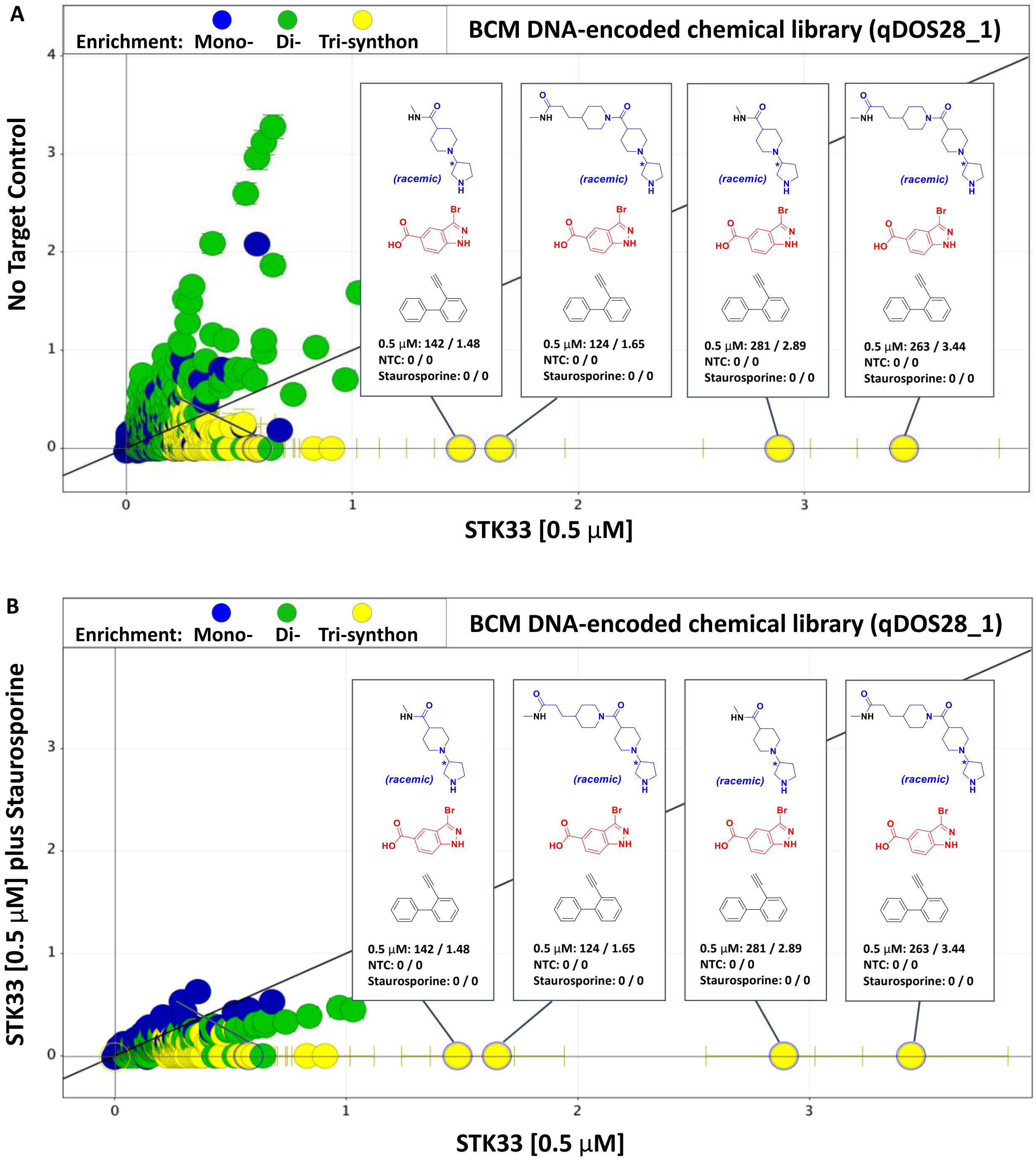

fig. S2. DEC-Tec selection and potent hits. (**A**) Enrichment profile of BCM DECL qDOS28_1 against STK33 at 0.5 µM (x-axis, z-score) versus no target control (y-axis, z-score). (**B**) Enrichment profile of BCM DECL qDOS28_1 against STK33 at 0.5 µM (x-axis, z-score) versus selection against STK33 at 0.5 µM performed in the presence of staurosporine (y-axis, z-score). Most hits are not observed in the presence of staurosporine, indicating competitive binding.

A

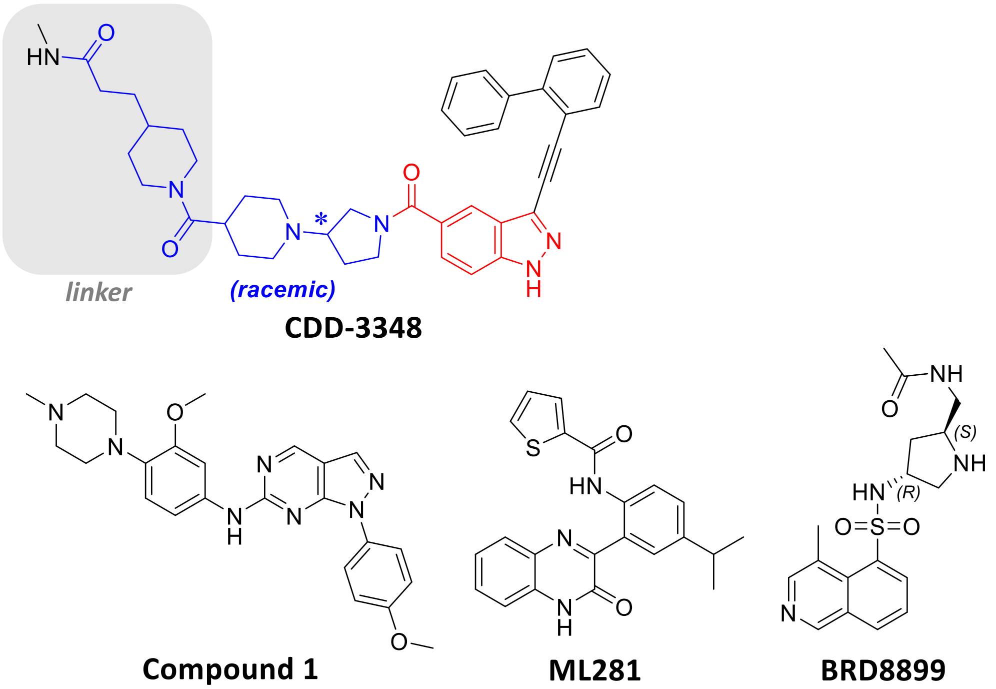

B

Compound			CDD-3348	Compound 1	ML281	BRD8899
Molecular weight (Da); cLogP			671; 3.7	446; 3.9	389; 3.2	362.5; 1.0
Biochemical activity	LanthaScreen K_d (nM)	STK33	0.06 ± 0.01	1.7 ± 0.2	39.6 ± 1.5	1.2 ± 0.1
		CLK4	26.2 ± 1.5	1.2 ± 0.1	>1000	>1000
	Z'LYTE K_i (nM)	CLK2	58.3 ± 3.3	8.7 ± 0.6	>1000	>1000
		RET	5.6 ± 0.5	2.6 ± 0.2	>1000	45.9 ± 8.7
		CLK1	477 ± 34	2.2 ± 1.7	>1000	>1000
Cellular activity	NanoBRET IC_{50} (nM)	STK33	169	750	7710	11800
		CLK4	3850 (23X)	41	ND	>40000
		CLK2	2530 (15X)	200	ND	>40000
		RET	6410 (38X)	960	ND	>40000
		CLK1	6730 (40X)	410	ND	>40000
Metabolic stability	MLM, $t_{1/2}$ (min)		14	ND	ND	ND
	HLM, $t_{1/2}$ (min)		13	ND	ND	ND

fig. S3. STK33 hits, known inhibitors, and biological characteristics. (**A**) Chemical structures of CDD-3348, compound 1, ML281, and BRD8899. CDD-3348 is the hit with a long linker enriched in the STK33 selection. Blue, red, and black moieties correspond to BB1, BB2, and BB3, respectively. Compound 1 (*32*), ML281 (*33*), and BRD8899 (*34*) are known STK33 inhibitors with reported IC_{50} values of 7, 14, and 11 nM, respectively, in biochemical assays. (**B**) Chemical properties, biochemical activity, cellular activity, and metabolism data for CDD-3348, compound 1, ML281, and BRD8899. K_d and K_i values were calculated from LanthaScreen binding assay and Z'-LYTE assay, respectively; IC_{50} values were calculated from NanoBRET assays; $t_{1/2}$ was measured using either MLM or HLM stability assay. ND, not determined.

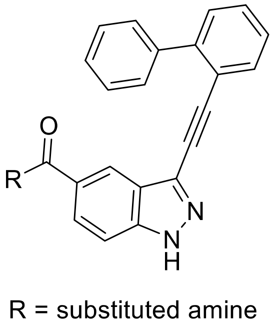

R = substituted amine

Compound	CDD-2211	S1 (CDD-2579)	S2 (CDD-2594)
**R**	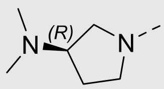	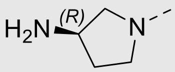	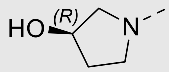
**NanoBRET, IC_{50} (nM)**	16	46	647
**MLM, $t_{1/2}$ (min)**	7	42	ND
**HLM, $t_{1/2}$ (min)**	10	277	ND

Compound	S3 (CDD-2765)	S4 (CDD-2766)	S5 (CDD-2674)
**R**	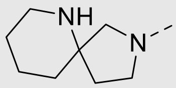	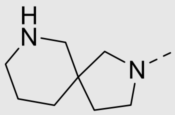	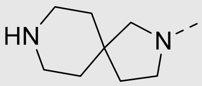
**NanoBRET, IC_{50} (nM)**	52	517	1294

Compound	S6 (CDD-2808)	CDD-2807	S7 (CDD-2806)
**R**	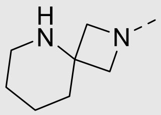	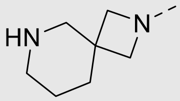	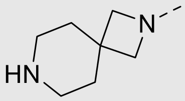
**NanoBRET, IC_{50} (nM)**	236	9.2	168
**MLM, $t_{1/2}$ (min)**	ND	71	46
**HLM, $t_{1/2}$ (min)**	ND	601	235

fig. S4. Discovery of CDD-2807 from CDD-2211 analogs. Among the analogs with different substituted amines, CDD-2807 demonstrated comparable cellular potency toward STK33 and improved microsomal stability in both MLM and HLM assays. Assay data >60 min is an extrapolated estimate. ND, not determined.

A

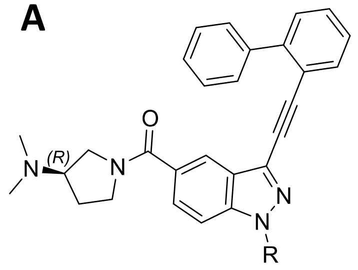

Compound	CDD-2211	S8 (CDD-2533)
**R**	H	CH_3
**NanoBRET, IC_{50} (nM)**	16	ND

B

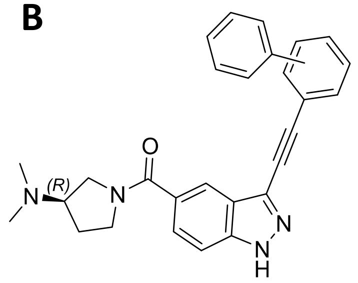

Compound	CDD-2211	S9 (CDD-2532)	S10 (CDD-2563)
**Biphenyl orientation**	*ortho*	*meta*	*para*
**LanthaScreen, K_d (nM)**	0.018 ± 0.004	9.9 ± 2.3	17.7 ± 0.7
**NanoBRET, IC_{50} (nM)**	16	289	8707

C

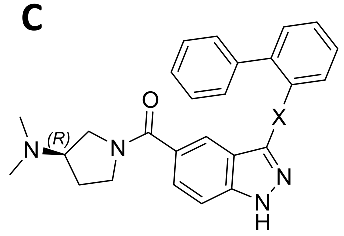

Compound	CDD-2211	S11 (CDD-2647)	S12 (CDD-2573)
**X**	C≡C	CH_2	null
**NanoBRET, IC_{50} (nM)**	16	ND	ND

fig. S5. Validation of STK33/CDD-2211 interactions by SAR analysis of CDD-2211 analogs. The hinge binding of the indazole (**A**; CDD-2211 versus **S8**), the *ortho*-orientation of the biphenyl (**B**; CDD-2211 versus **S9** and **S10**), and the two-carbon distance between the indazole and the biphenyl (**C**; CDD-2211 versus **S11** and **S12**) all contributed to the potency toward STK33. ND (not determined) for compounds not passing kinase assay screening against STK33 at 500 nM.

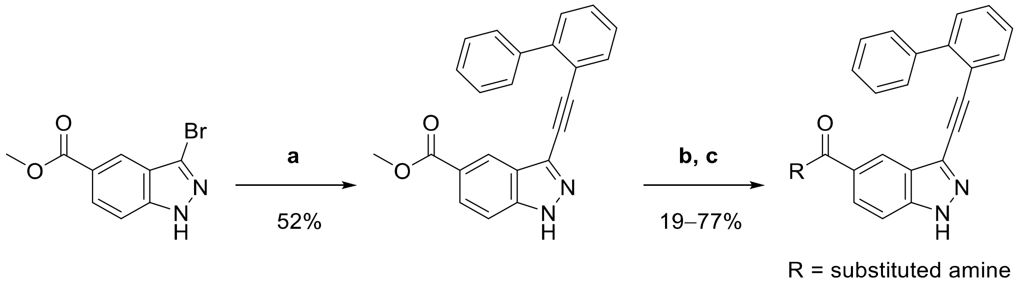

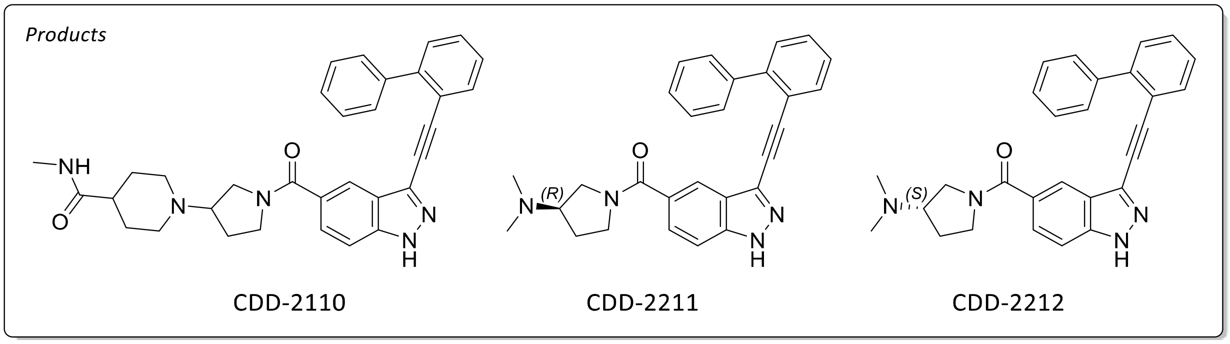

fig. S6. Synthetic scheme for the preparation of CDD-2110, CDD-2211, and CDD-2212. Reagents and conditions: (a) Sonogashira coupling: 2-ethynyl-1,1'-biphenyl (1.2 equiv.), CuI (0.1 equiv.), $PdCl_2(PPh_3)_2$ (0.1 equiv.), DMF/TEA (1:1, *v/v*), microwave 80°C, 1 h; (b) hydrolysis without THP-protected indazole: KOH (10.0 equiv.), THF/water (1:1, *v/v*), 40°C, 48 h; (c) amide coupling: different substituted amine (1.2 equiv.), HATU (1.2 equiv.), DIEA (1.5 equiv.), DMF, rt, 16 h.

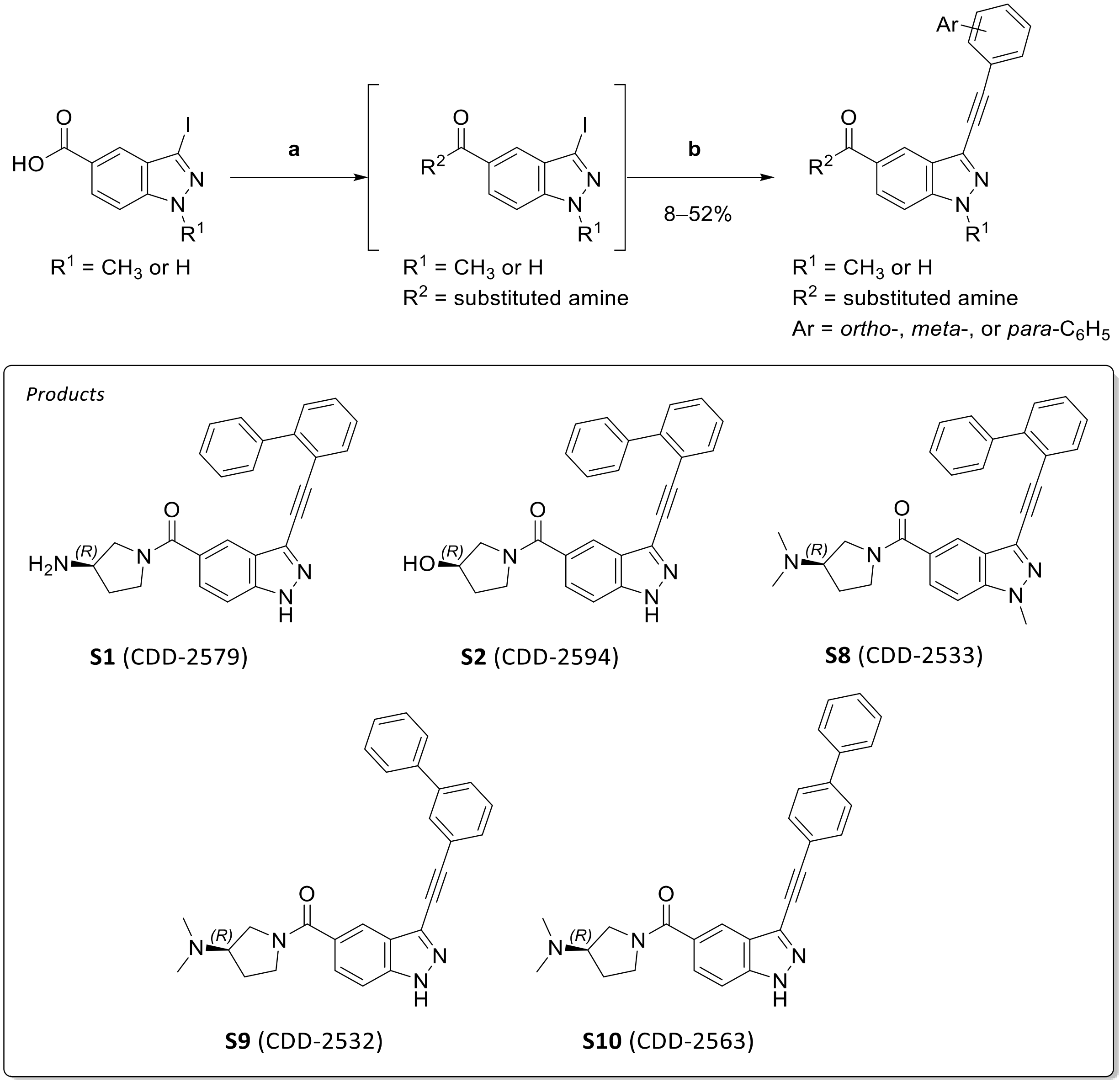

fig. S7. Synthetic scheme for the preparation of compounds S1, S2, and S8–S10. Reagents and conditions: (a) amide coupling: different substituted amine (1.2 equiv.), HATU (1.2 equiv.), DIEA (1.5 equiv.), DMF, rt, 16 h; (b) Sonogashira coupling: different substituted alkyne (1.2 equiv.), CuI (0.1 equiv.), $PdCl_2(PPh_3)_2$ (0.1 equiv.), DMF/TEA (1:1, *v/v*), microwave 80°C, 1 h.

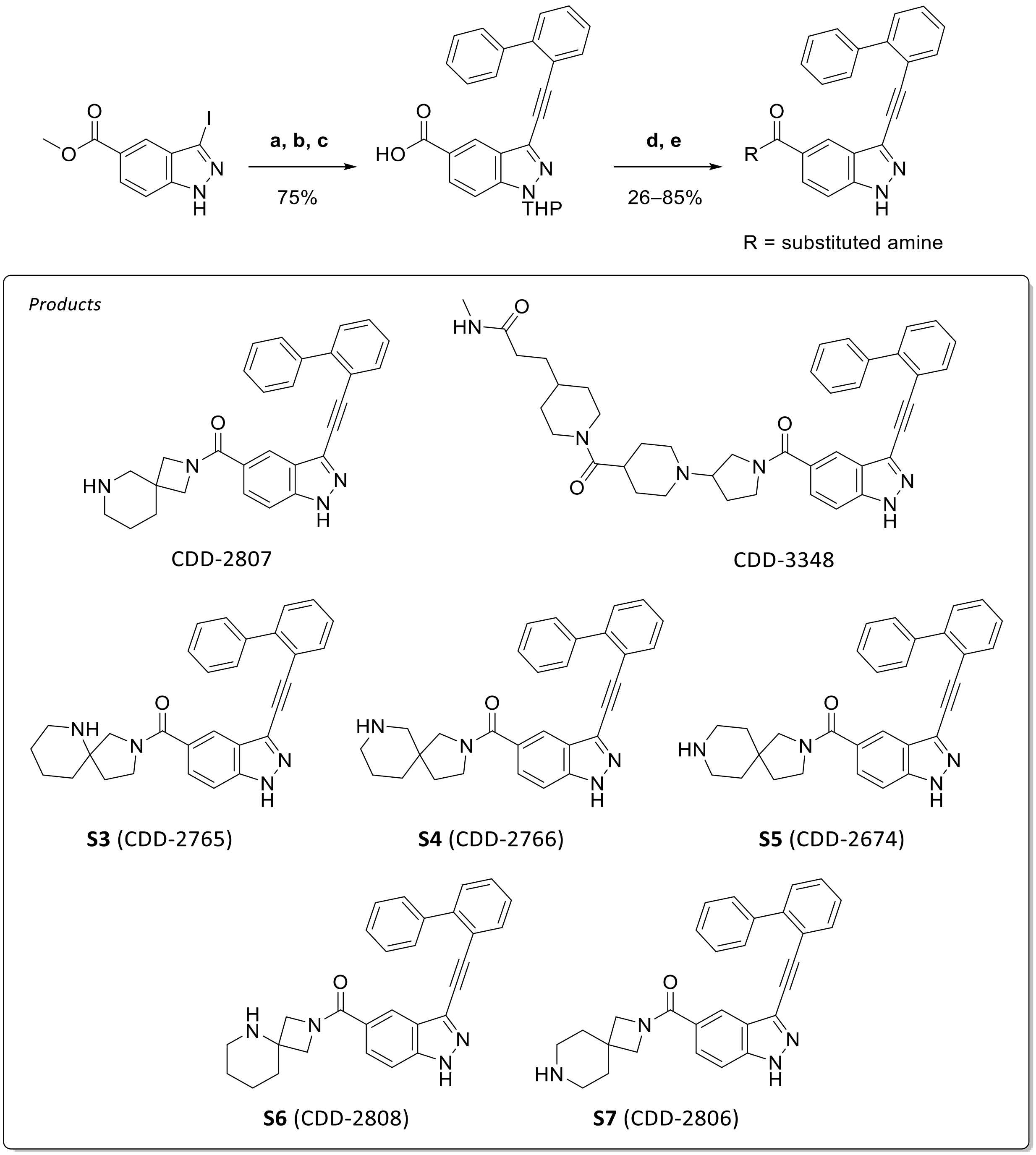

fig. S8. Synthetic scheme for the preparation of CDD-2807, CDD-3348, and compounds S3–S7. Reagents and conditions: (a) THP protection: DHP (1.5 equiv.), *p*-TsOH·H_2O (0.2 equiv.), CH_2Cl_2, rt, 16 h; (b) Sonogashira coupling: 2-ethynyl-1,1'-biphenyl (1.2 equiv.), CuI (0.1 equiv.), $PdCl_2(PPh_3)_2$ (0.1 equiv.), DMF/TEA (1:1, *v/v*), microwave 80°C, 1 h; (c) hydrolysis with THP-protected indazole: LiOH·H_2O (2.0 equiv.), THF/water (1:1, *v/v*), rt, 1 h; (d) amide coupling: different substituted amine (1.2 equiv.), HATU (1.2 equiv.), DIEA (1.5–3.0 equiv.), DMF, rt, 16 h; (e) removal of THP and/or Boc-protecting group: 4N HCl in 1,4-dioxane, rt, 1 h.

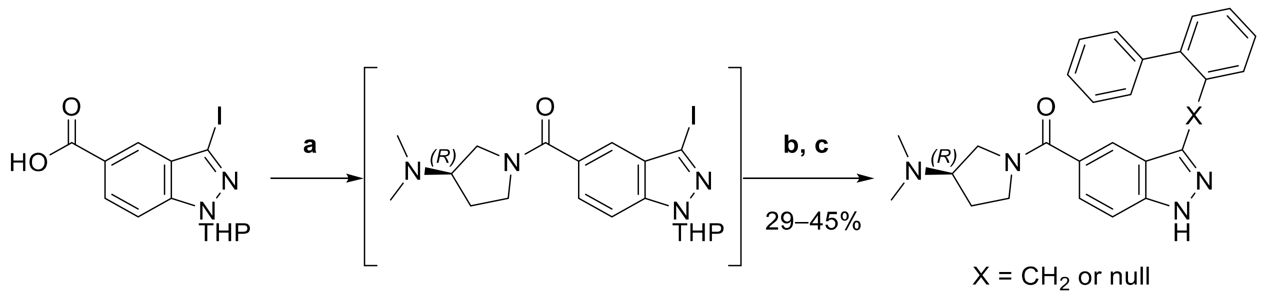

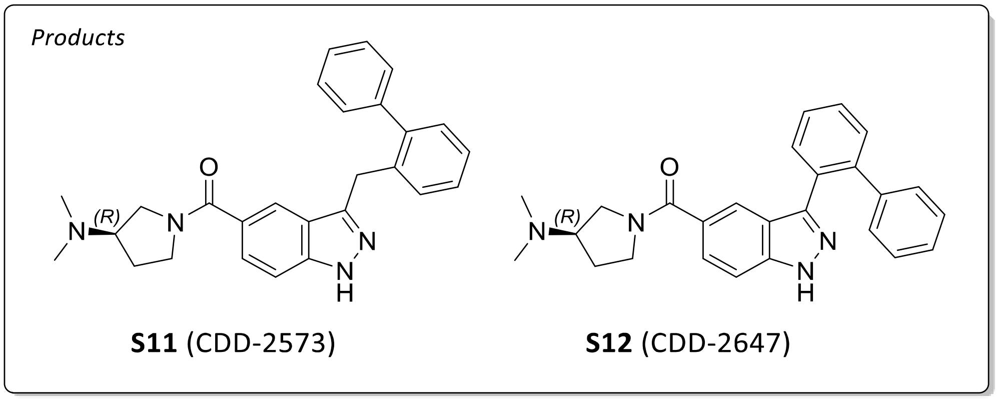

fig. S9. Synthetic scheme for the preparation of compounds S11 and S12. Reagents and conditions: (a) amide coupling: (*R*)-*N*,*N*-dimethyl-3-pyrrolidinamine (1.2 equiv.), HATU (1.2 equiv.), DIEA (1.5 equiv.), DMF, rt, 16 h; (b) X = CH_2: potassium ([1,1'-biphenyl]-2-ylmethyl)trifluoroborate (2.5 equiv.), $Pd(OAc)_2$ (0.1 equiv.), RuPhos (0.2 equiv.), Cs_2CO_3 (3.0 equiv.), toluene/water (10:1, *v/v*); X = null: boronic acid derivative (2.0 equiv.), $Pd(PPh_3)_4$ (0.1 equiv.), K_2CO_3 (2.0 equiv.), DMF/water (3:1, *v/v*), microwave 100°C, 1 h; (c) removal of THP: 4N HCl in 1,4-dioxane, rt, 1 h.

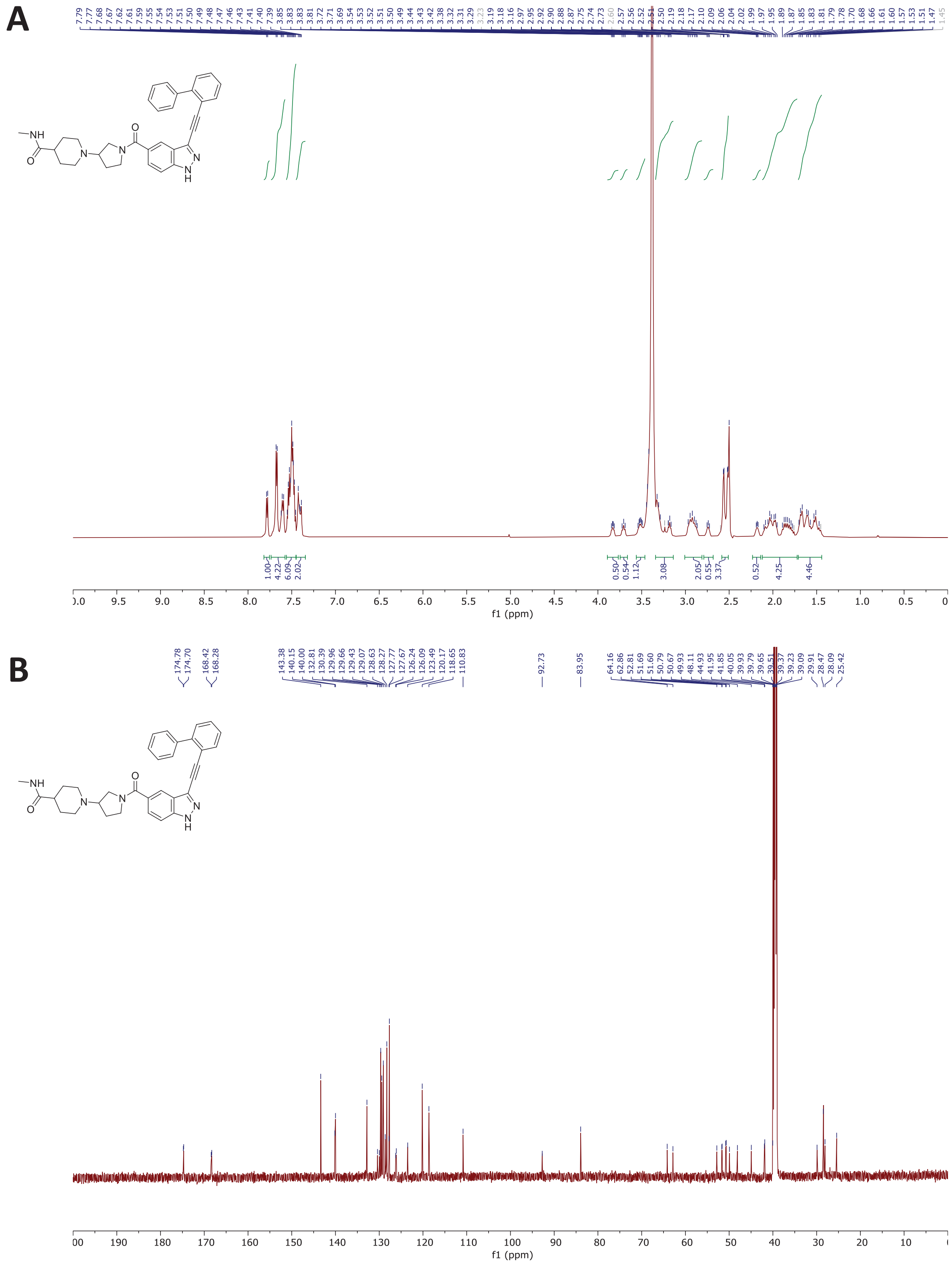

fig. S10. NMR spectra of CDD-2110. (**A**) ^{1}H NMR (DMSO-d_6, 600 MHz) spectrum. (**B**) ^{13}C NMR (DMSO-d_6, 150 MHz) spectrum.

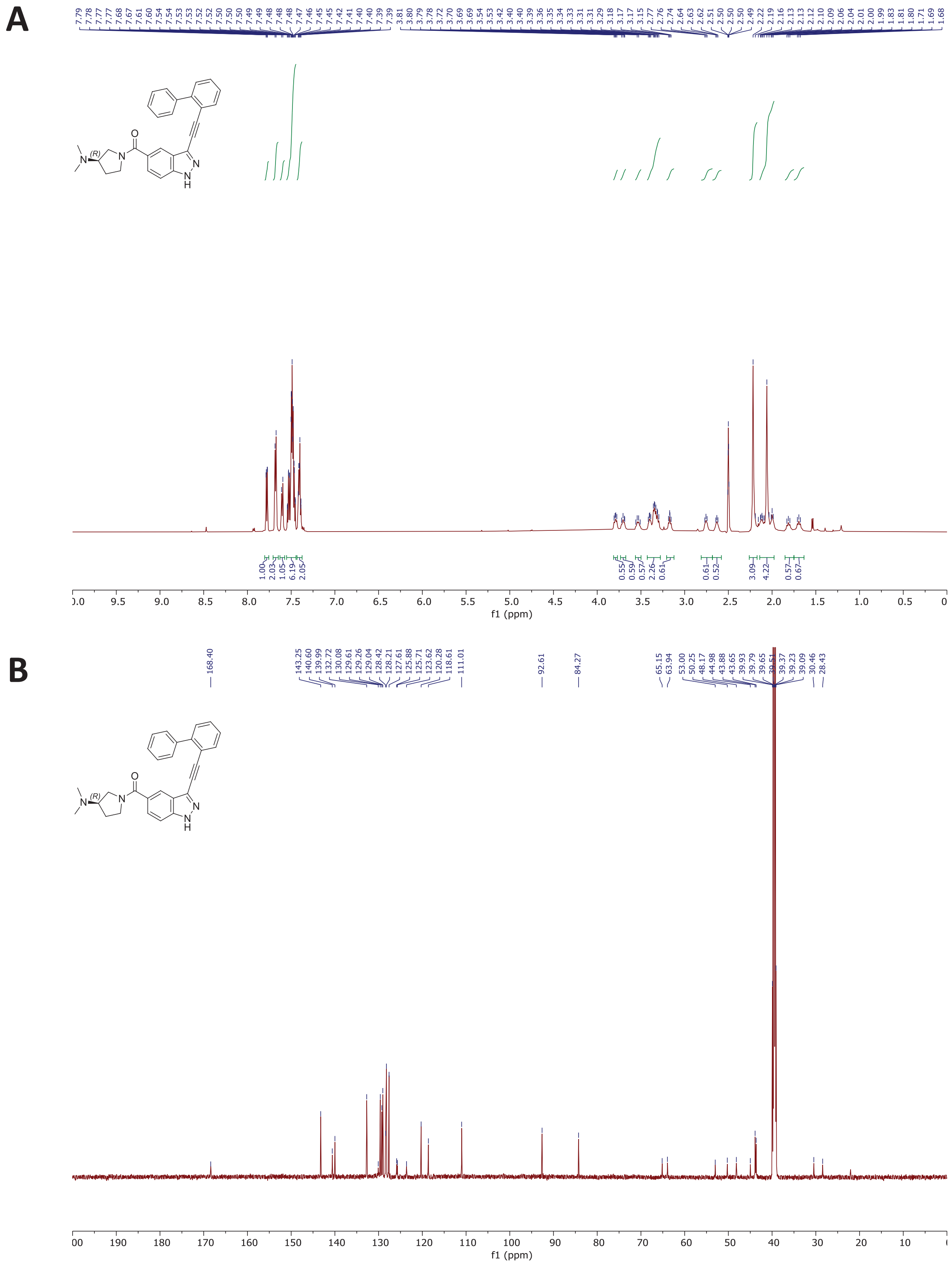

fig. S11. NMR spectra of CDD-2211. (**A**) ^{1}H NMR (DMSO-d_6, 600 MHz) spectrum. (**B**) ^{13}C NMR (DMSO-d_6, 150 MHz) spectrum.

A

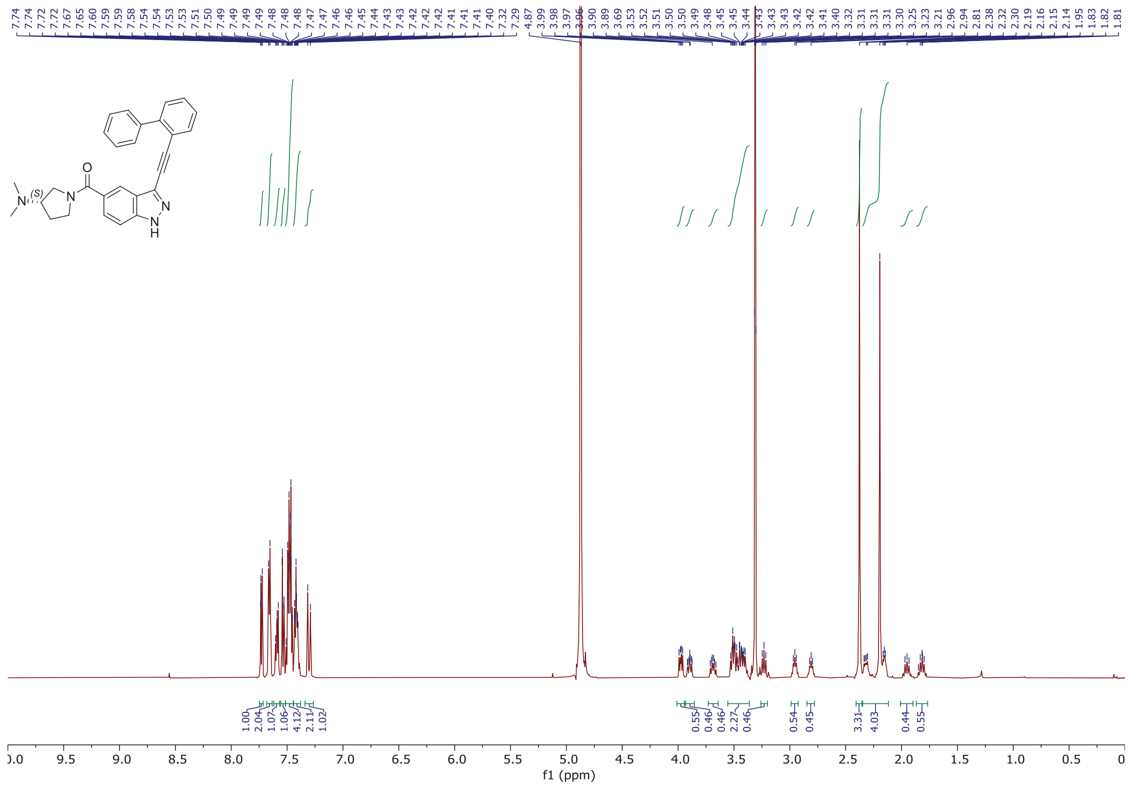

B

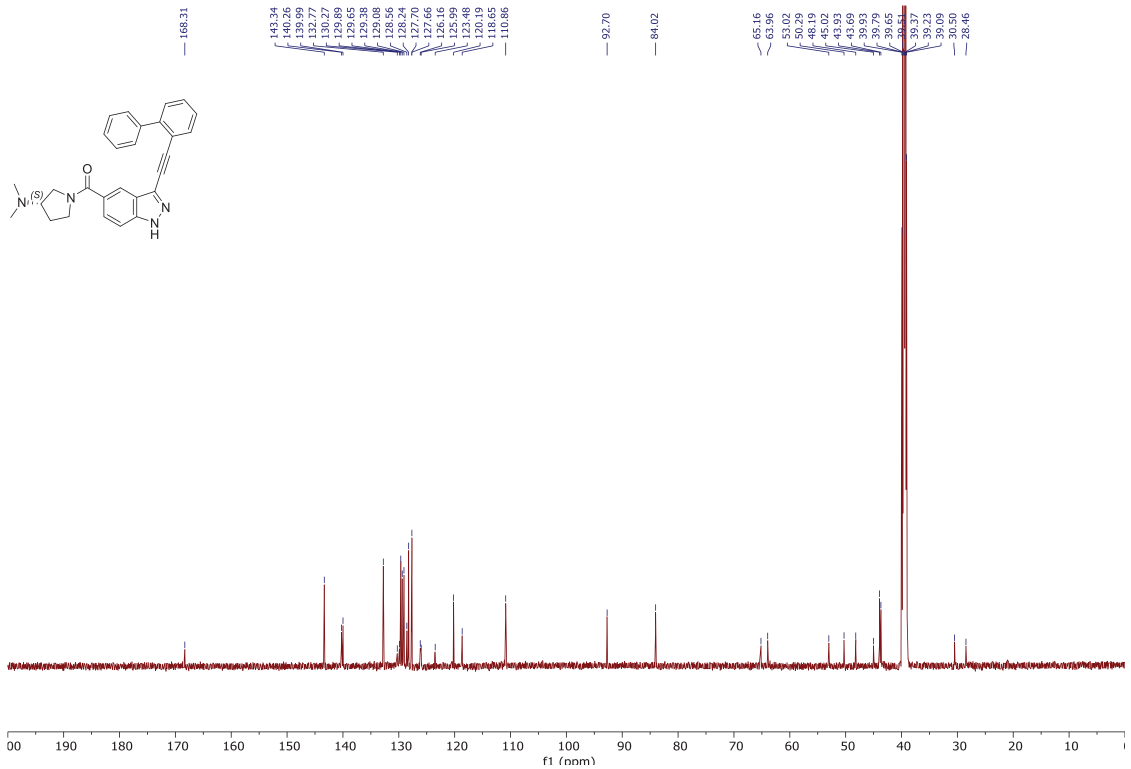

fig. S12. NMR spectra of CDD-2212. (**A**) ^{1}H NMR (DMSO-d_6, 600 MHz) spectrum. (**B**) ^{13}C NMR (DMSO-d_6, 150 MHz) spectrum.

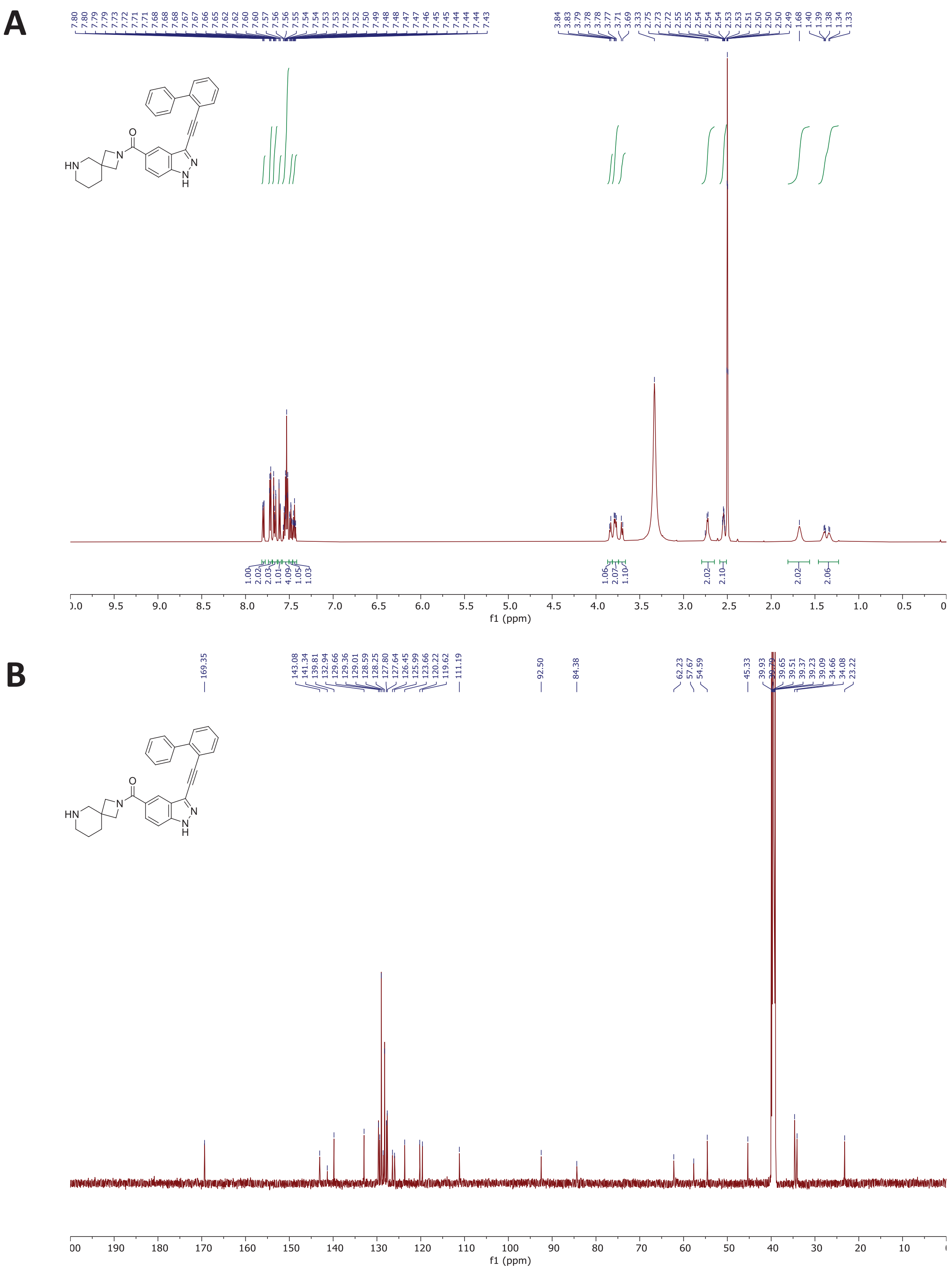

fig. S13. NMR spectra of CDD-2807. (**A**) ^{1}H NMR (DMSO-d_6, 600 MHz) spectrum. (**B**) ^{13}C NMR (DMSO-d_6, 150 MHz) spectrum.

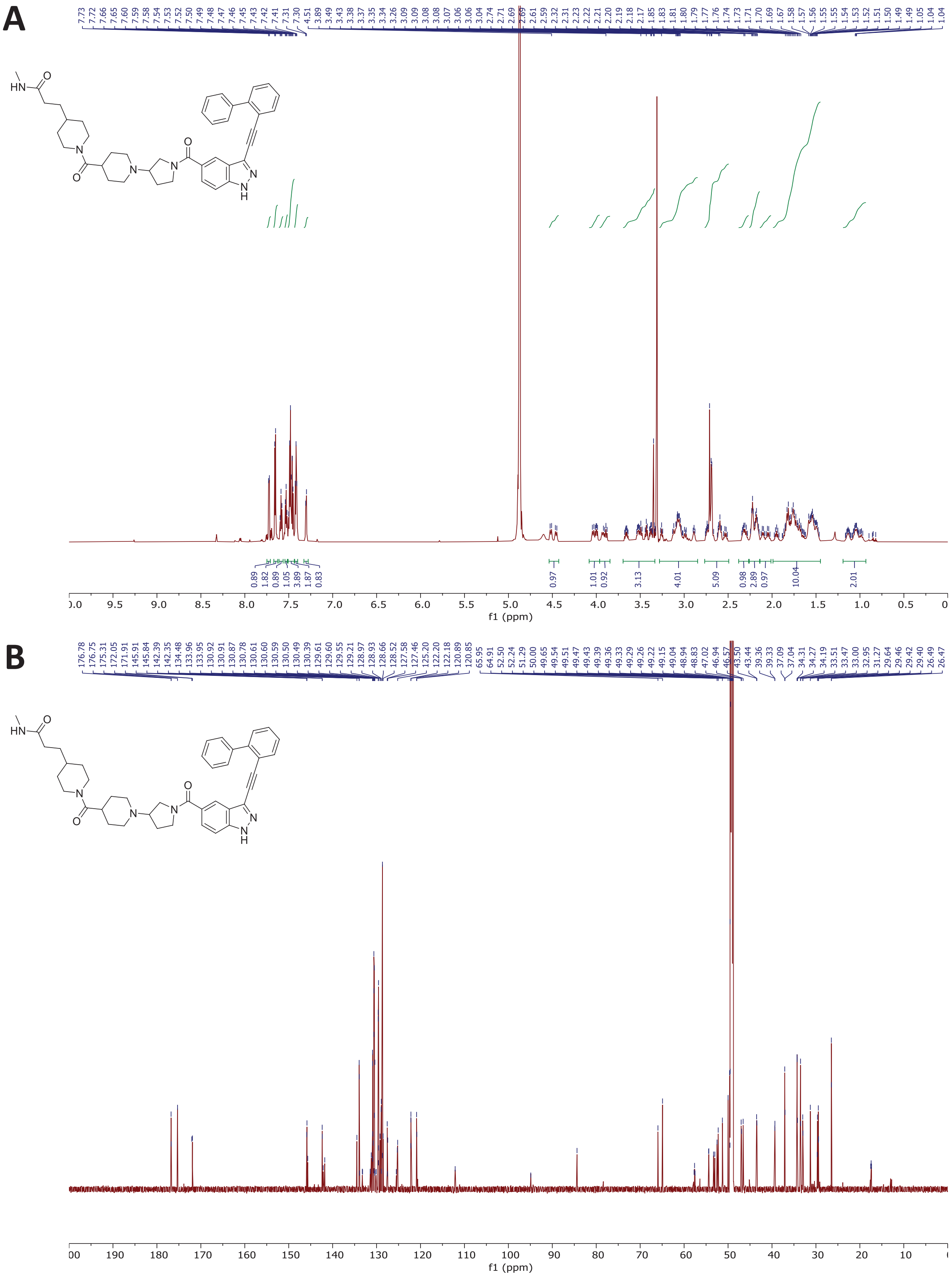

fig. S14. NMR spectra of CDD-3348. (**A**) ^{1}H NMR (CD_3OD, 800 MHz) spectrum. (**B**) ^{13}C NMR (CD_3OD, 200 MHz) spectrum.

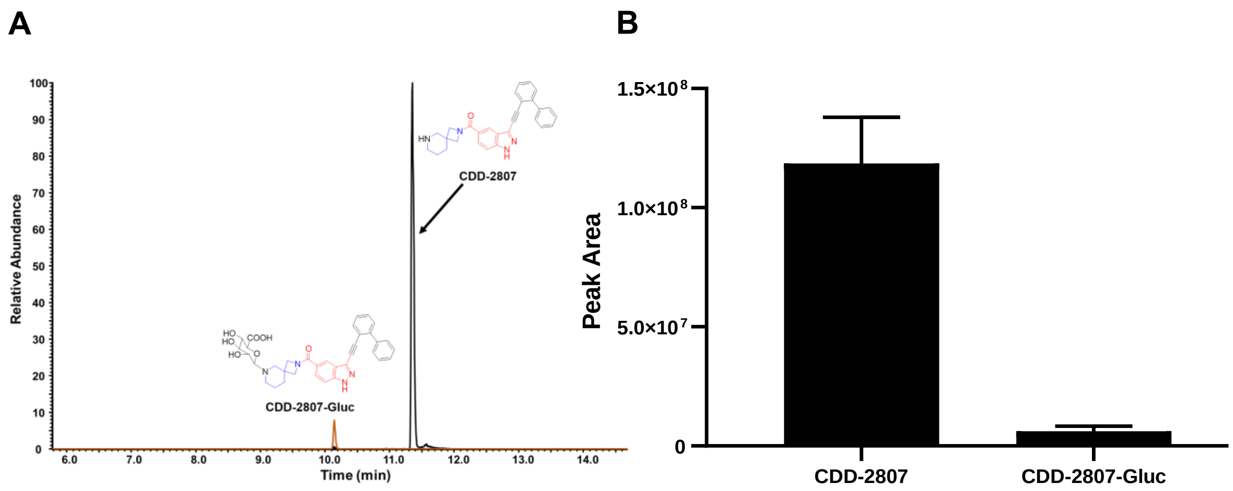

fig. S15. Glucuronidation of CDD-2807 in mice. (**A**) Representative chromatograms of CDD-2807 and CDD-2807-glucuronide (Gluc) in mouse plasma. (**B**) Relative abundances of CDD-2807 and CDD-2807-Gluc in mouse plasma. The mice were treated with CDD-2807 (25 mg/kg, i.p., n = 4). The plasma samples were collected 2 hours after the treatment and analyzed by a Thermo Q Exploris 120 MS coupled with a Thermo Vanquish UHPLC.

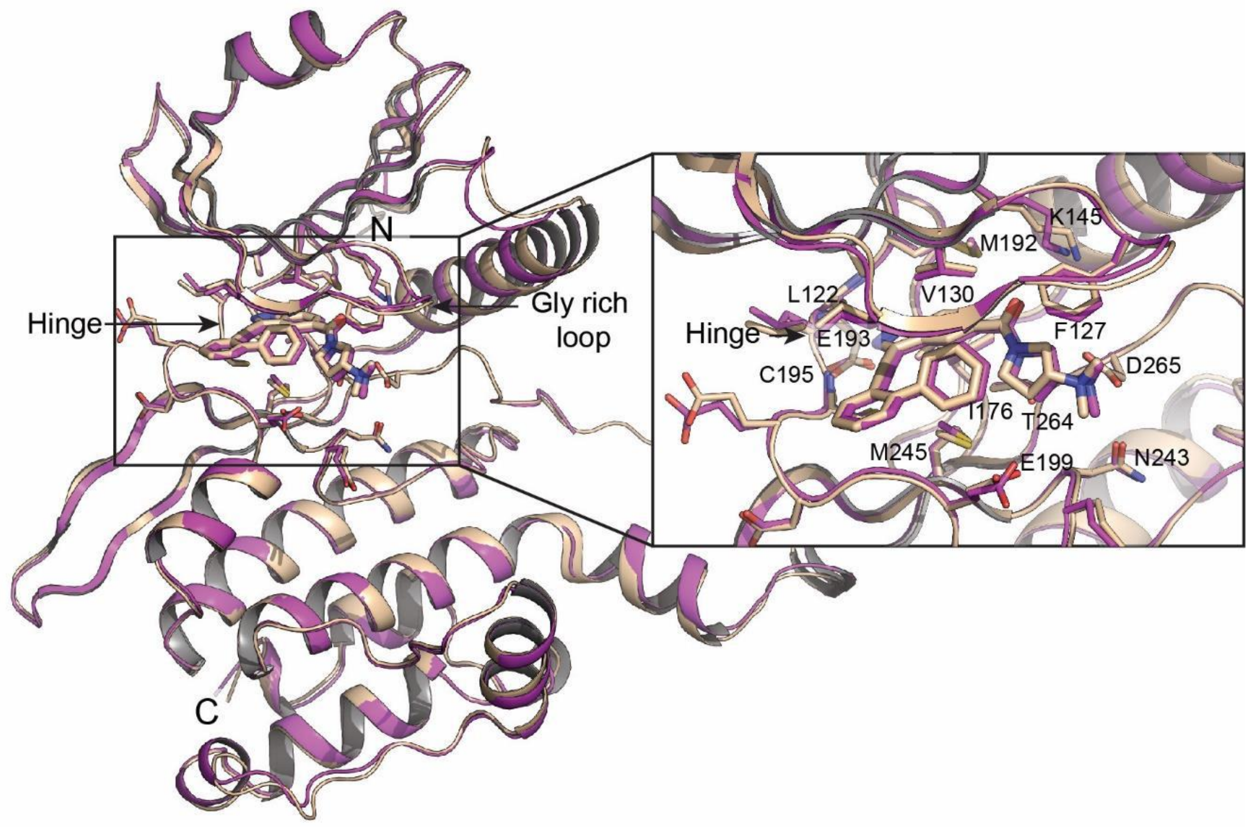

fig. S16. Superimposition of the two chains of the STK33/CDD-2211 dimer complex. A magnified image of the boxed region shows the bound CDD-2211 molecules with residues within 5 Å of CDD-2211. Chain A is colored in tan and chain B in magenta. Two chains are nearly identical showing an RMSD of 0.46 Å between shared 245 Cα atoms. Only Cα atoms are used in aligning the two chains.

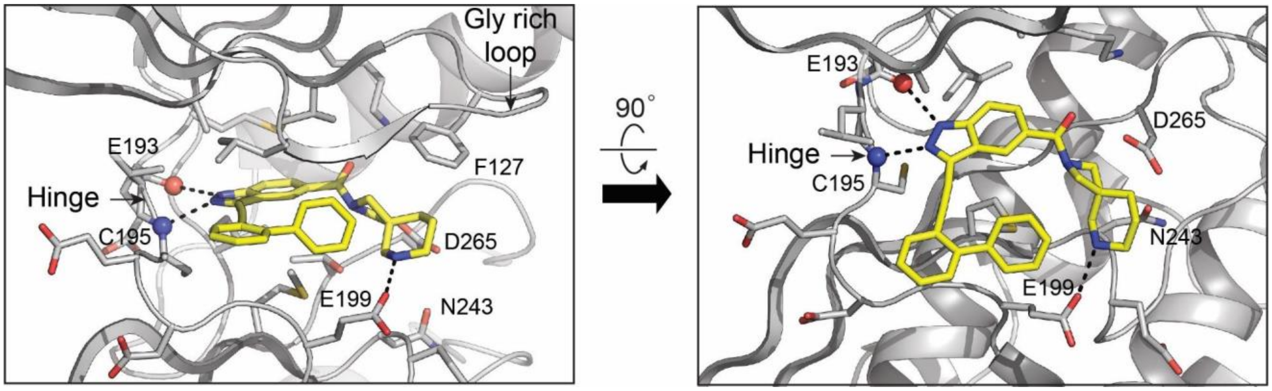

fig. S17. A model of CDD-2807 docked to the active site of human STK33. The color theme is the same as that in Fig. 3D.

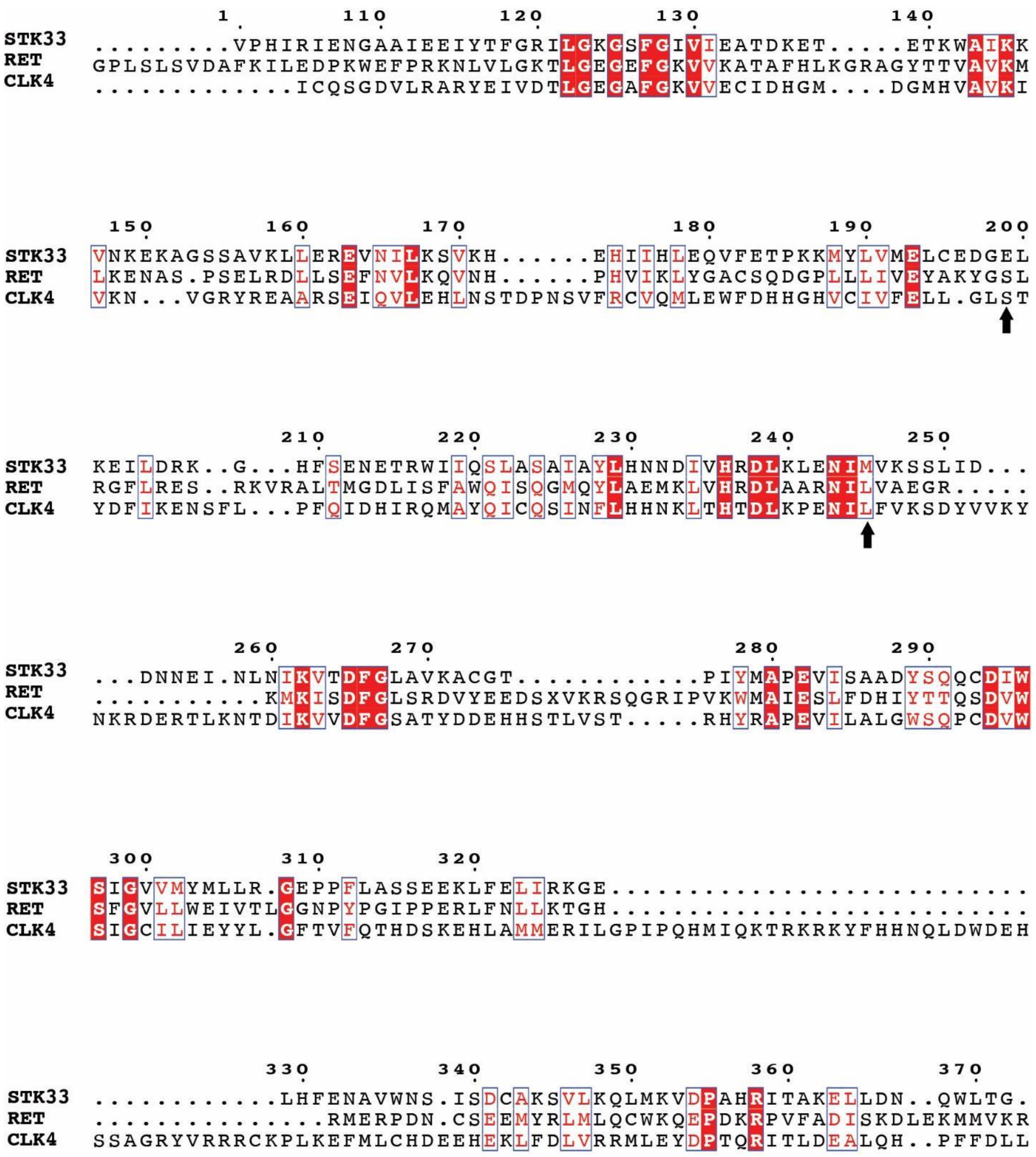

fig. S18. Structure based sequence alignment of STK33, RET, and CLK4. The CDD-2211 contact residues, E199 and M245, which are unique to STK33, are marked with arrows. The alignment was performed using PROMALS3D employing FAST and TM-align (*74*). PDB IDs 4CJK (RET) and 6FYV (CLK4) are used for aligning with STK33 KD.

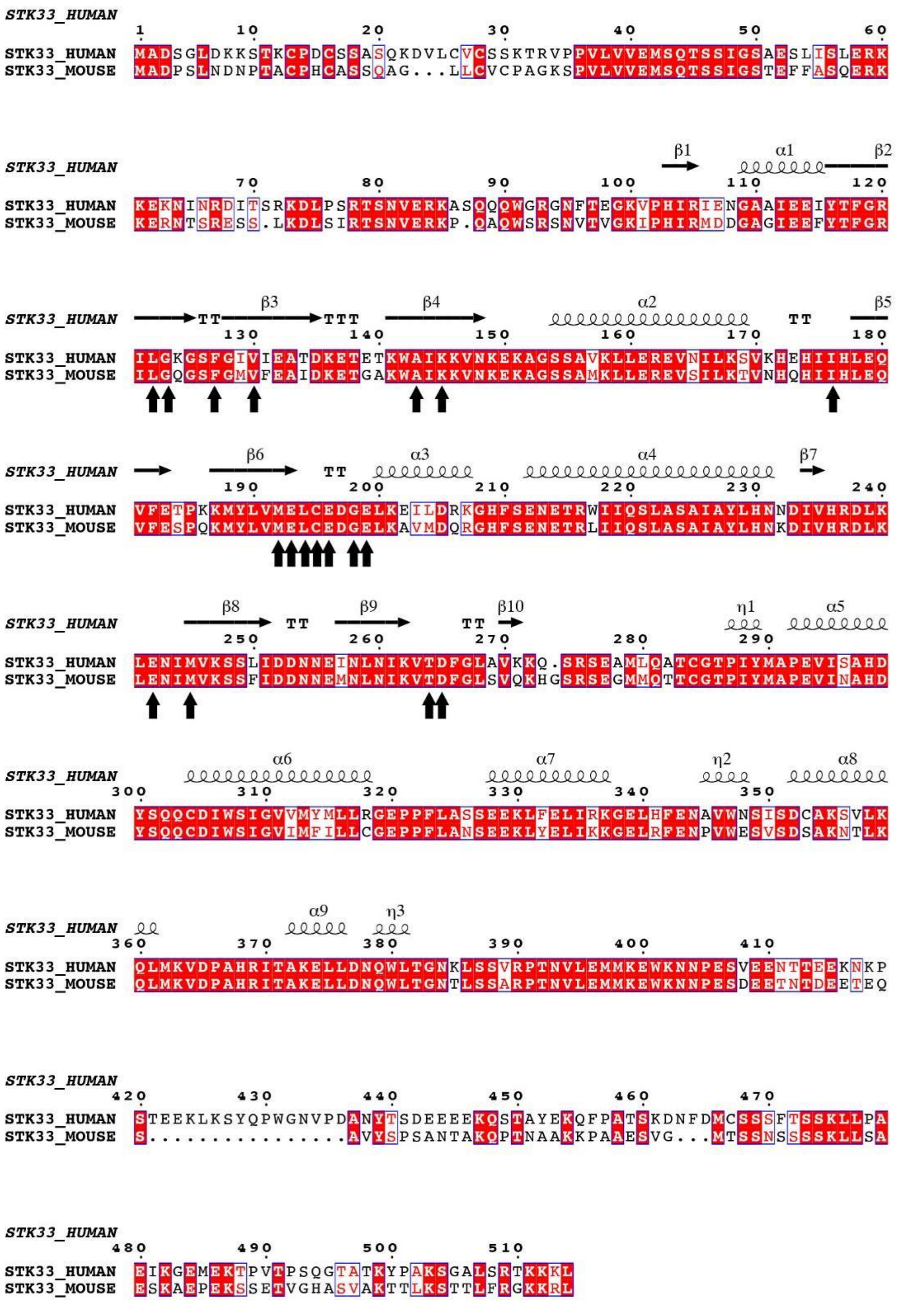

fig. S19. Sequence alignment between human and mouse STK33 proteins. Sequence alignment was performed using the CLUSTAL W program (*75*) and plotted with the ENDscript program (*76*). Identical amino acids, as identified by the default ESPript parameters (Risler, global score 0.7), are highlighted with white in red background; similar residues are in red. The secondary structures of human STK33 kinase domain are indicated above the sequence. CDD-2211 contacting residues in the human STK33/CDD-2211 complex are marked with arrows.

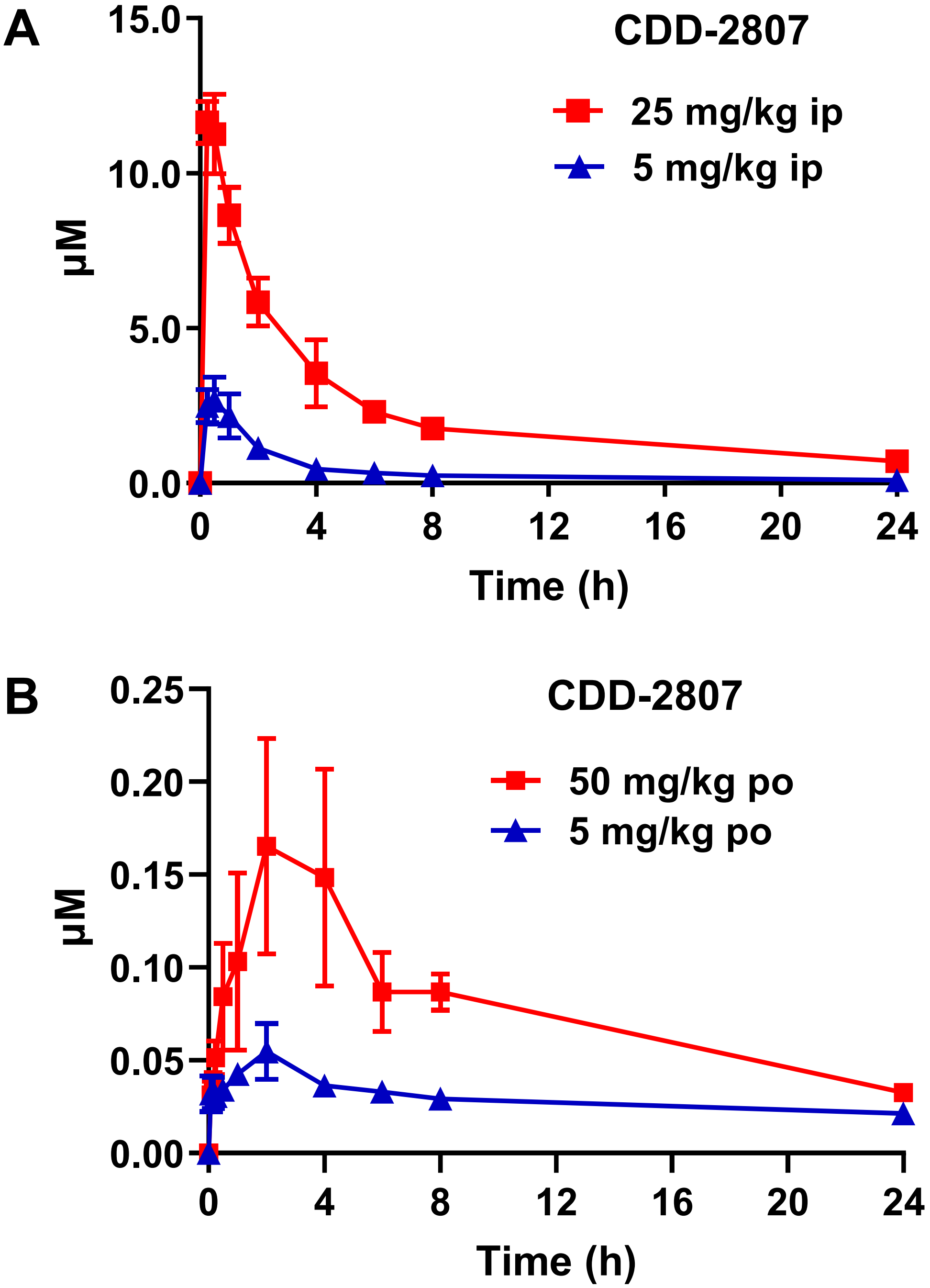

fig. S20. Evaluation of CDD-2807 PK in mice (n = 3 for each group). (**A**) PK of CDD-2807 via i.p. injection. (**B**) PK of CDD-2807 via oral administration.

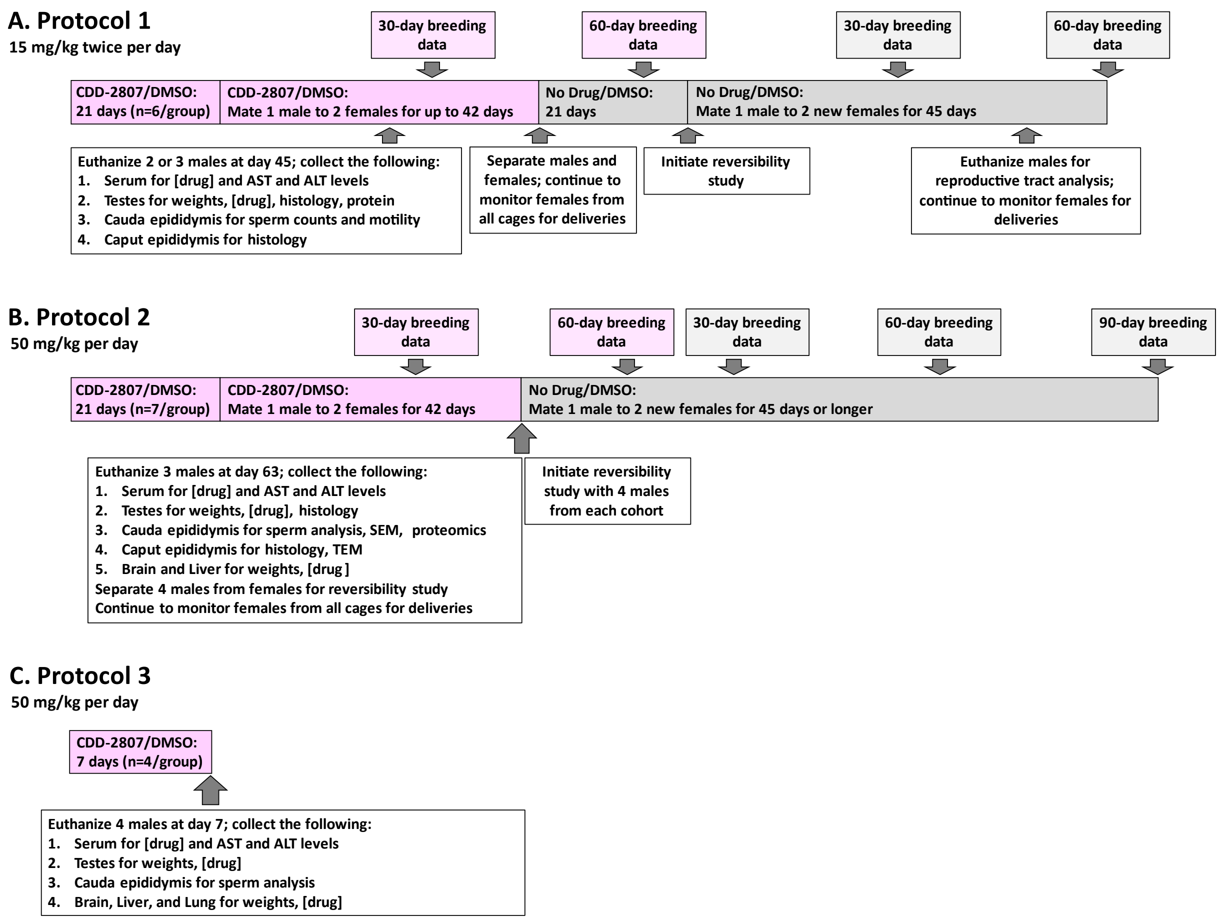

fig. S21. Protocol timelines of mouse breeding and reversibility study. (**A**) Protocol 1 represents the 15 mg/kg twice per day drug dosing of CDD-2807. Samples were collected at euthanasia on day 45 from half of the male mice, allowing for the completion of the 30-day breeding data to be collected and to give the male mice sufficient time with their female counterparts to initiate the day 60 breeding data. After completion of the entire 60-day breeding data, protocol 1 mice were then “rested” for 21 days prior to measuring their recovery period where they were housed with females to determine fertility. (**B**) Protocol 2 represents the 50 mg/kg per day drug dosing of CDD-2807. Samples were collected at euthanasia on day 63 from half of the male mice; this allowed for the entire completion of the initial breeding data prior to collections. Remaining mice were immediately shifted into the recovery period where we monitored males with females to determine the interval for the males to resume fertility. (**C**) Protocol 3 represents the 50 mg/kg per day short drug dosing of CDD-2807. Samples were collected at euthanasia on day 7. No breeding protocol was established with Protocol 3.

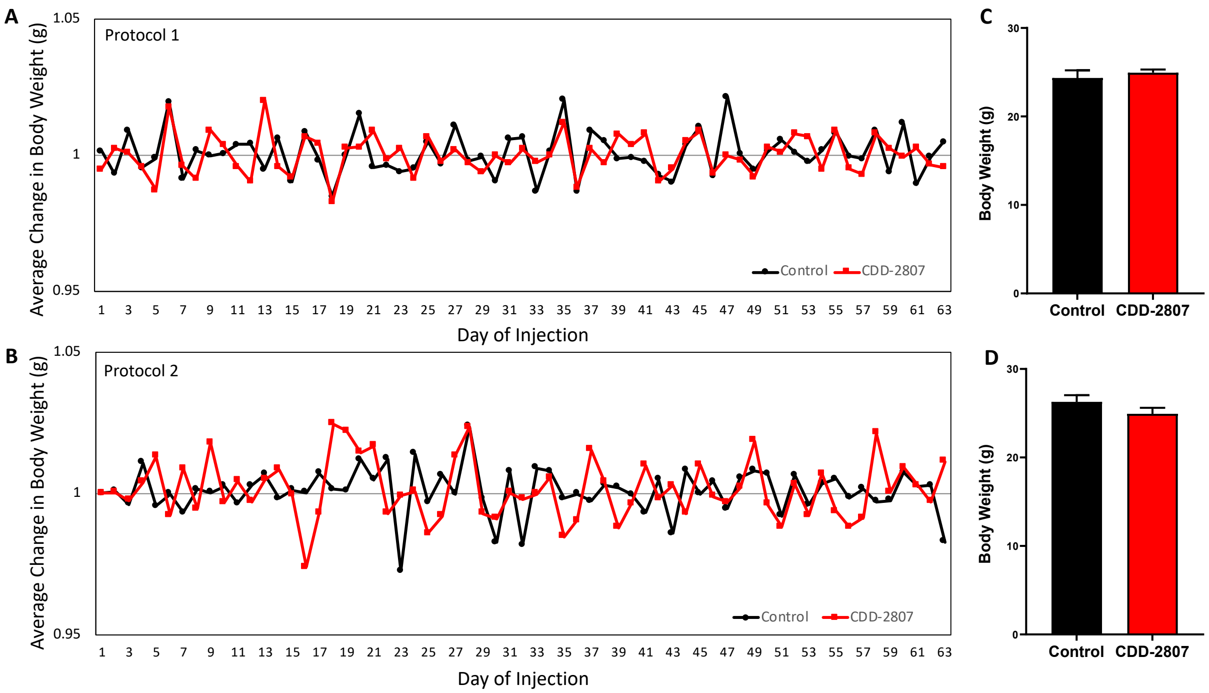

fig. S22. No body weight changes in mice treated long-term with CDD-2807. Body weight changes during treatment in protocol 1 (**A**; n = 3–6 mice) and body weight changes during treatment in protocol 2 (**B**; n = 4–7 mice). Body weights of protocol 1 mice euthanized at day 45 in protocol 1 (**C**; n = 2–3 mice) and body weights of protocol 2 mice euthanized at day 63 (**D**; n = 3 mice).

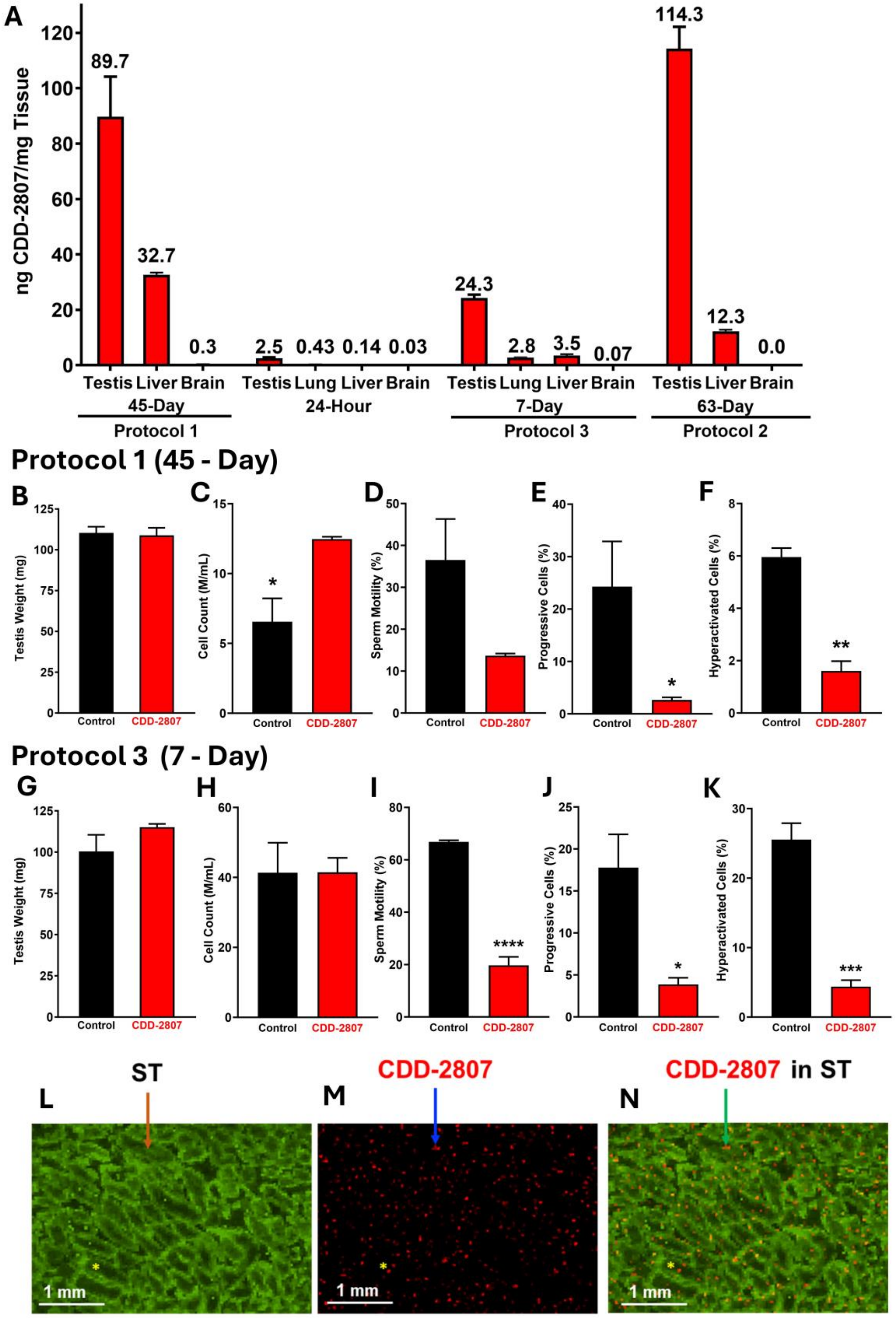

Lipid on ST: Green (m/z 798.5406); CDD-2807: Red (m/z 447.2179)

fig. S23. CDD-2807 crosses the blood-testis barrier to alter sperm parameters but testis weight in mice. (**A**) Concentrations of CDD-2807 in the tissues of protocol 1 (15 mg/kg twice per day) mice (n = 2) at day 45 and protocol 2 & 3 (50 mg/kg/day) mice at multiple time points (24-hours (n = 4), 7-days (n = 4), and day 63 (n = 3)); no CDD-2807 was observed in control mouse tissues. (**B**) Despite the contraceptive effect of CDD-2807, testis size of CDD-2807-treated mice from protocol 1 (n = 2) at day 45 were normal. (**C**) Vehicle control mice (n = 2) in protocol 1 unexpectedly had a low sperm count. In protocol 1, the control mice did have a lower-than-expected average cell count for a control group; we expected a value around 12 M/mL, and in this collection, we had an average of 6.55 M/mL which was unexpected. (**D**–**F**) CDD-2807-treated mice (n = 3) had decreased sperm motility (**D**), progressive sperm (**E**), and hyperactivated sperm (**F**) compared to vehicle control mice (n = 2). An additional collection for protocol 3 was taken after 7 days (**G**–**K**); in this collection, there was no observed difference between testis weight (**G**) between control and treatment groups (n = 4). While there was no difference in sperm count (**H**), there were observed differences in motility (**I**), progressive sperm cells (**J**), and hyperactivated sperm cells (**K**). Spatial distribution of a lipid with m/z 798.5406 (positive mode) outlines the seminiferous tubules (ST) (**L**), spatial distribution of CDD-2807 (positive mode) (**M**), and overlayed imaging of lipid and CDD-2807 (**N**). Yellow star: the second representative ST. The data were collected on the Bruker TimsTOF MS MALDI-2 instrument with the resolution at 40 µm/pixel. The ions were extracted within mass accuracy of ±3.5 ppm. P-values: <0.05 = *, <0.01 = **, <0.001 = ***, <0.0001 = ****. Scale bars are 1 mm.

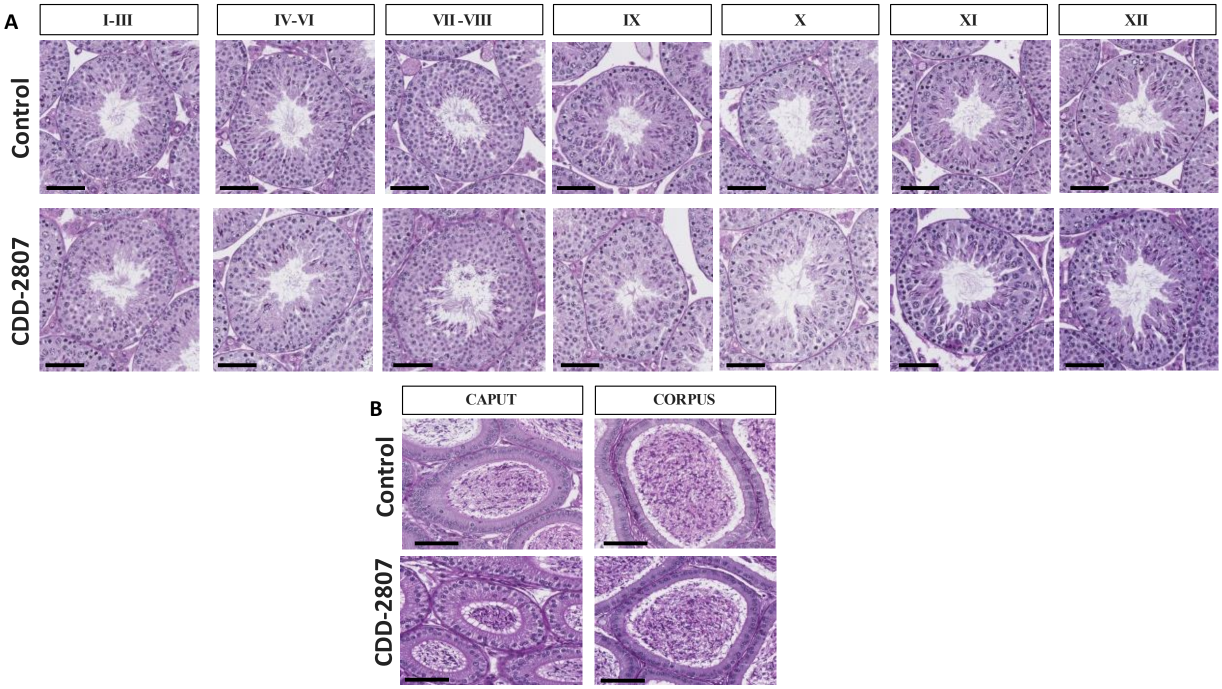

fig. S24. Histological analysis of testis and epididymis segments (caput & corpus) from control and CDD-2807-treated male mice from protocol 1. Bouin's fixed, 4 µm-cross sections of the testis (**A**) and horizontal sections of the epididymis (**B**) were stained with PAS-hematoxylin. All 12 stages of spermatogenesis are represented in both control and CDD-2807-treated mice and show normal tissue morphology and acrosome development. Epididymis from the control and CDD-2807-treated mice were similar histologically. Scale bars are 60 µm.

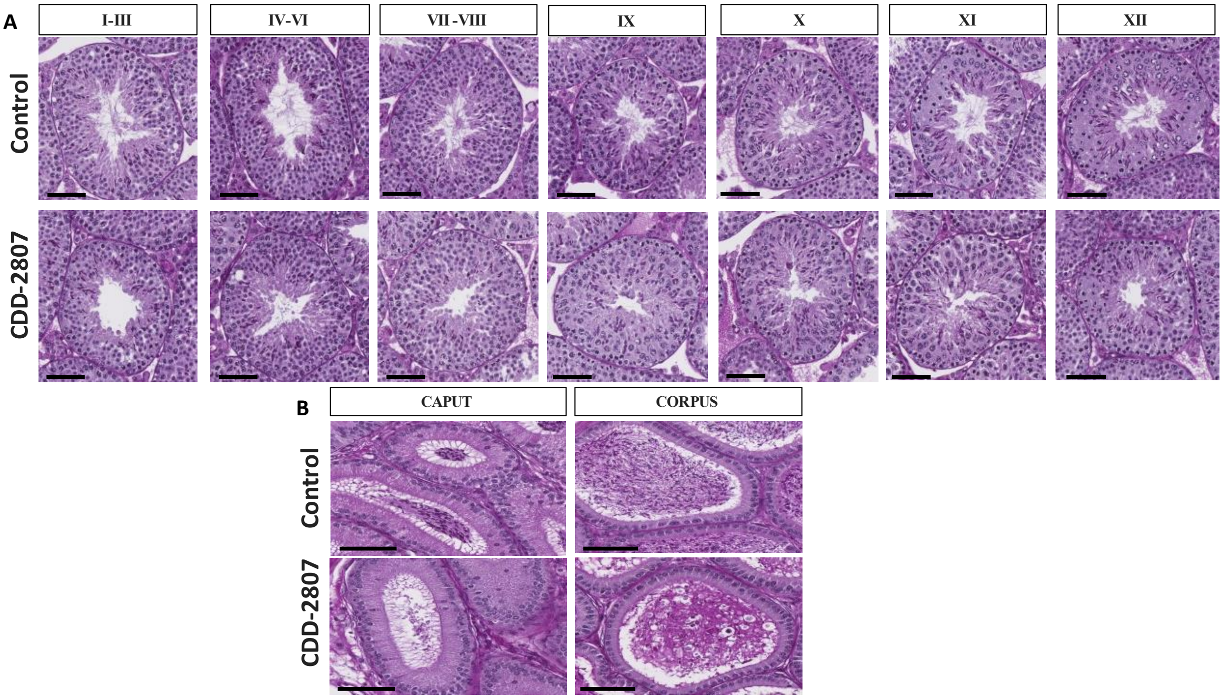

fig. S25. Histological analysis of testis and epididymis segments (caput & corpus) from control and CDD-2807-treated male mice from protocol 2. Bouin's fixed, 4 µm-cross sections of the testis (**A**) and longitudinal sections of the epididymis (**B**) were stained with PAS-hematoxylin. All 12 stages of spermatogenesis are represented in both control and CDD-2807-treated mice, although it was difficult to find all stages in the CDD-2807-treated mice. Epididymis from the control and CDD-2807-treated mice were very different histologically, control mice had normal sperm cells, while CDD-2807 mice had occasional round spermatids present in the epididymis. Scale bars are 60 µm.

Supplementary tables

Table S1. Data collection and refinement statistics.

Complex	STK33/CDD-2211
PDB ID code	8VF6
Data collection	
Space group	$P\ 2_12_12_1$
Cell Dimensions	
a, b, c (Å)	84.1 87.93 98.45
α, β, γ (°)	90, 90, 90
Resolution (Å)	43.94–2.70 (2.83–2.70)[a]
I/ σ (I)	7.0 (1.0)
Wilson B-factor	67.6
R_{merge}	0.05979 (0.6425)
R_{pim}	0.149 (1.53)
$CC_{1/2}$	0.994 (0.605)
Completeness (%)	96.77 (100)
Multiplicity	8.1 (8.6)
No. of unique reflections	19879 (1037)
R_{work}/R_{free}[b]	21.46/25.67 (35.01/42.15)
No. of atoms	
Protein	4272
Ligand	66
B-factors	
Protein	73.9
Ligands	52.6
R.m.s.[c] *deviations*	
Bond length (Å)	0.004
Bond angles (°)	0.77
Ramachandran plot (%)	
Favored region	96.64
Allowed region	3.17
Outliers	0.19

[a] Highest resolution shell is shown in parenthesis.
[b] 5% of the observed intensities was excluded from refinement for cross validation purposes.
[c] *R.m.s.*, root mean square.

Table S2. Liver microsomal metabolic stability and PK studies of CDD-2807.

(**A**) The half-life of CDD-2807 in liver microsomes with or without glucuronidation

Half-life of CDD-2807	liver microsomes	liver microsomes with glucuronidation
MLM, $t_{1/2}$ (min)	71	50
HLM, $t_{1/2}$ (min)	601	388

(**B**) Noncompartmental PK parameters for CDD-2807 (n = 3, mean ± S.E.M.)

**Parameter**	**Unit**	**i.p. injection**		**p.o. administration**	
		5 mg/kg	25 mg/kg	5 mg/kg	50 mg/kg
T_{max}	h	0.33 ± 0.14	0.33 ± 0.14	1.03 ± 0.96	2.67 ± 1.15
C_{max}	μM	2.95 ± 0.64	11.90 ± 0.40	0.06 ± 0.02	0.18 ± 0.09
$t_{1/2}$	h	11.90 ± 4.38	11.30 ± 3.41	36.14 ± 19.91	12.64 ± 1.50
AUC_{0-t}	h*μM	9.46 ± 1.47	55.48 ± 5.42	0.71 ± 0.07	1.88 ± 0.36
$AUC_{0-\infty}$	h*μM	11.17 ± 1.11	67.73 ± 12.41	1.85 ± 0.66	2.47 ± 0.34
CL/F	mg/(h*μM)/kg	7.77 ± 3.20	5.92 ± 0.79	2.91 ± 0.87	20.52 ± 3.02
Vd/F	mg/μM/kg	0.45 ± 0.04	0.38 ± 0.08	134.88 ± 26.14	376.76 ± 89.83
$MRT_{0-\infty}$	h	11.14 ± 4.43	12.24 ± 3.80	51.25 ± 28.94	16.46 ± 2.80

Table S3. Serum concentrations of ALT (IU/L) and AST (IU/L) for control and CDD-2807-treated mice from each collection point.

Protocol 1

45 Days

	ALT (IU/L)	AST (IU/L)	N
Control	47.74 ± 5.30	48.18 ± 23.43	2
CDD-2807	20.33 ± 5.30	35.80 ± 3.09	2

Protocol 2

7 Days

	ALT (IU/L)	AST (IU/L)	N
Control	31.53 ± 5.10	37.72 ± 10.80	4
CDD-2807	23.65 ± 2.50	21.66 ± 3.30	4

63 Days

	ALT (IU/L)	AST (IU/L)	N
Control	37.72 ± 13.54	42.14 ± 14.93	3
CDD-2807	45.97 ± 11.04	29.76 ± 6.79	3

References and notes

1. Available from https://www.worldometers.info/world-population/#milestones.

2. J. Castaneda, M. M. Matzuk, Toward a rapid and reversible male pill. *Science* **350**, 385–386 (2015). doi:10.1126/science.aad4425 Medline

3. Available from: http://consensusforaction.stanford.edu/see-scientific-consensus/consensus_english.pdf.

4. D. J. Anderson, D. S. Johnston, A brief history and future prospects of contraception. *Science* **380**, 154–158 (2023). doi:10.1126/science.adf9341 Medline

5. R. J. Aitken, M. A. Baker, G. F. Doncel, M. M. Matzuk, C. K. Mauck, M. J. K. Harper, As the world grows: Contraception in the 21st century. *J. Clin. Invest.* **118**, 1330–1343 (2008). doi:10.1172/JCI33873 Medline

6. K. Heinemann, F. Saad, M. Wiesemes, S. White, L. Heinemann, Attitudes toward male fertility control: Results of a multinational survey on four continents. *Hum. Reprod.* **20**, 549–556 (2005). doi:10.1093/humrep/deh574 Medline

7. B. T. Nguyen, T. L. Jacobsohn, Men's willingness to use novel male contraception is linked to gender-equitable attitudes: Results from an exploratory online survey. *Contraception* **123**, 110001 (2023). doi:10.1016/j.contraception.2023.110001 Medline

8. M. M. Matzuk, D. J. Lamb, The biology of infertility: Research advances and clinical challenges. *Nat. Med.* **14**, 1197–1213 (2008). doi:10.1038/nm.f.1895 Medline

9. S. T. Page, D. Blithe, C. Wang, Hormonal male contraception: Getting to market. *Front. Endocrinol.* **13**, 891589 (2022). doi:10.3389/fendo.2022.891589 Medline

10. H. M. Behre, M. Zitzmann, R. A. Anderson, D. J. Handelsman, S. W. Lestari, R. I. McLachlan, M. C. Meriggiola, M. M. Misro, G. Noe, F. C. W. Wu, M. P. R. Festin, N. A. Habib, K. M. Vogelsong, M. M. Callahan, K. A. Linton, D. S. Colvard, Efficacy and safety of an injectable combination hormonal contraceptive for men. *J. Clin. Endocrinol. Metab.* **101**, 4779–4788 (2016). doi:10.1210/jc.2016-2141 Medline

11. A. Thirumalai, J. Ceponis, J. K. Amory, R. Swerdloff, V. Surampudi, P. Y. Liu, W. J. Bremner, E. Harvey, D. L. Blithe, M. S. Lee, L. Hull, C. Wang, S. T. Page, Effects of 28 days of oral dimethandrolone undecanoate in healthy Men: A prototype male pill. *J. Clin. Endocrinol. Metab.* **104**, 423–432 (2019). doi:10.1210/jc.2018-01452 Medline

12. M. Y. Roth, G. Shih, N. Ilani, C. Wang, S. T. Page, W. J. Bremner, R. S. Swerdloff, R. Sitruk-Ware, D. L. Blithe, J. K. Amory, Acceptability of a transdermal gel-based male hormonal contraceptive in a randomized controlled trial. *Contraception* **90**, 407–412 (2014). doi:10.1016/j.contraception.2014.05.013 Medline

13. M. J. Robertson, K. Kent, N. Tharp, K. Nozawa, L. Dean, M. Mathew, S. L. Grimm, Z. Yu, C. Légaré, Y. Fujihara, M. Ikawa, R. Sullivan, C. Coarfa, M. M. Matzuk, T. X. Garcia, Large-scale discovery of male reproductive tract-specific genes through analysis of RNA-seq datasets. *BMC Biol.* **18**, 103 (2020). doi:10.1186/s12915-020-00826-z Medline

14. M. M. Matzuk, M. R. McKeown, P. Filippakopoulos, Q. Li, L. Ma, J. E. Agno, M. E. Lemieux, S. Picaud, R. N. Yu, J. Qi, S. Knapp, J. E. Bradner, Small-molecule inhibition

of BRDT for male contraception. *Cell* **150**, 673–684 (2012). doi:10.1016/j.cell.2012.06.045 Medline

15. M. Lyon, P. Li, J. J. Ferreira, R. M. Lazarenko, S. V. Kharade, M. Kramer, S. J. McClenahan, E. Days, J. A. Bauer, B. D. Spitznagel, C. D. Weaver, A. Borrego Alvarez, L. C. Puga Molina, P. Lybaert, S. Khambekar, A. Liu, C. W. Lindsley, J. Denton, C. M. Santi, A selective inhibitor of the sperm-specific potassium channel SLO3 impairs human sperm function. *Proc. Natl. Acad. Sci. U.S.A.* **120**, e2212338120 (2023). doi:10.1073/pnas.2212338120 Medline

16. M. Balbach, T. Rossetti, J. Ferreira, L. Ghanem, C. Ritagliati, R. W. Myers, D. J. Huggins, C. Steegborn, I. C. Miranda, P. T. Meinke, J. Buck, L. R. Levin, On-demand male contraception via acute inhibition of soluble adenylyl cyclase. *Nat. Commun.* **14**, 637 (2023). doi:10.1038/s41467-023-36119-6 Medline

17. E. B. Faber, N. Wang, K. John, L. Sun, H. L. Wong, D. Burban, R. Francis, D. Tian, K. H. Hong, A. Yang, L. Wang, M. Elsaid, H. Khalid, N. M. Levinson, E. Schönbrunn, J. E. Hawkinson, G. I. Georg, Screening through lead optimization of high affinity, allosteric cyclin-dependent kinase 2 (CDK2) inhibitors as male contraceptives that reduce sperm counts in mice. *J. Med. Chem.* **66**, 1928–1940 (2023). doi:10.1021/acs.jmedchem.2c01731 Medline

18. J. L. Kyzer, M. A. A. Noman, R. A. D. Cuellar, S. S. W. Chung, S. Maitra, T. Naqvi, J. E. Hawkinson, D. J. Wolgemuth, G. I. Georg, Investigation of selective retinoic acid receptor alpha antagonist ER-50891 and related analogs for male contraception. *Arch. Pharm.* **356**, e2300031 (2023). doi:10.1002/ardp.202300031 Medline

19. Z. Chang, W. Qin, H. Zheng, K. Schegg, L. Han, X. Liu, Y. Wang, Z. Wang, H. McSwiggin, H. Peng, S. Yuan, J. Wu, Y. Wang, S. Zhu, Y. Jiang, H. Nie, Y. Tang, Y. Zhou, M. J. M. Hitchcock, Y. Tang, W. Yan, Triptonide is a reversible non-hormonal male contraceptive agent in mice and non-human primates. *Nat. Commun.* **12**, 1253 (2021). doi:10.1038/s41467-021-21517-5 Medline

20. M. G. O'Rand, K. G. Hamil, T. Adevai, M. Zelinski, Inhibition of sperm motility in male macaques with EP055, a potential non-hormonal male contraceptive. *PLOS ONE* **13**, e0195953 (2018). doi:10.1371/journal.pone.0195953 Medline

21. G. Manning, D. B. Whyte, R. Martinez, T. Hunter, S. Sudarsanam, The protein kinase complement of the human genome. *Science* **298**, 1912–1934 (2002). doi:10.1126/science.1075762 Medline

22. A. Alonso, J. Sasin, N. Bottini, I. Friedberg, I. Friedberg, A. Osterman, A. Godzik, T. Hunter, J. Dixon, T. Mustelin, Protein tyrosine phosphatases in the human genome. *Cell* **117**, 699–711 (2004). doi:10.1016/j.cell.2004.05.018 Medline

23. K. S. Bhullar, N. O. Lagarón, E. M. McGowan, I. Parmar, A. Jha, B. P. Hubbard, H. P. V. Rupasinghe, Kinase-targeted cancer therapies: Progress, challenges and future directions. *Mol. Cancer* **17**, 48 (2018). doi:10.1186/s12943-018-0804-2 Medline

24. M. M. Attwood, D. Fabbro, A. V. Sokolov, S. Knapp, H. B. Schiöth, Trends in kinase drug discovery: Targets, indications and inhibitor design. *Nat. Rev. Drug Discov.* **20**, 839–861 (2021). doi:10.1038/s41573-021-00252-y Medline

25. R. Roskoski Jr., Properties of FDA-approved small molecule protein kinase inhibitors: A 2021 update. *Pharmacol. Res.* **165**, 105463 (2021). doi:10.1016/j.phrs.2021.105463 Medline

26. F. Carles, S. Bourg, C. Meyer, P. Bonnet, PKIDB: A curated, annotated and updated database of protein kinase inhibitors in clinical trials. *Molecules* **23**, 908 (2018). doi:10.3390/molecules23040908 Medline

27. M. E. Berginski, N. Moret, C. Liu, D. Goldfarb, P. K. Sorger, S. M. Gomez, The Dark Kinase Knowledgebase: An online compendium of knowledge and experimental results of understudied kinases. *Nucleic Acids Res.* **49** (D1), D529–D535 (2021). doi:10.1093/nar/gkaa853 Medline

28. Mammalian Genetic Database, version 2; https://orit.research.bcm.edu/MRGDv2.

29. A. O. Mujica, B. Brauksiepe, S. Saaler-Reinhardt, S. Reuss, E. R. Schmidt, Differential expression pattern of the novel serine/threonine kinase, STK33, in mice and men. *FEBS J.* **272**, 4884–4898 (2005). doi:10.1111/j.1742-4658.2005.04900.x Medline

30. L. R. Martins, R. K. Bung, S. Koch, K. Richter, L. Schwarzmüller, D. Terhardt, B. Kurtulmus, C. Niehrs, A. Rouhi, I. Lohmann, G. Pereira, S. Fröhling, H. Glimm, C. Scholl, Stk33 is required for spermatid differentiation and male fertility in mice. *Dev. Biol.* **433**, 84–93 (2018). doi:10.1016/j.ydbio.2017.11.007 Medline

31. H. Ma, B. Zhang, A. Khan, D. Zhao, A. Ma, J. Zhou, I. Khan, K. Khan, H. Zhang, Y. Zhang, X. Jiang, S. Dil, A. Zeb, F. Rahim, Q. Shi, Novel frameshift mutation in *STK33* is associated with asthenozoospermia and multiple morphological abnormalities of the flagella. *Hum. Mol. Genet.* **30**, 1977–1984 (2021). doi:10.1093/hmg/ddab165 Medline

32. C. Babij, Y. Zhang, R. J. Kurzeja, A. Munzli, A. Shehabeldin, M. Fernando, K. Quon, P. D. Kassner, A. A. Ruefli-Brasse, V. J. Watson, F. Fajardo, A. Jackson, J. Zondlo, Y. Sun, A. R. Ellison, C. A. Plewa, M. T. San, J. Robinson, J. McCarter, R. Schwandner, T. Judd, J. Carnahan, I. Dussault, STK33 kinase activity is nonessential in KRAS-dependent cancer cells. *Cancer Res.* **71**, 5818–5826 (2011). doi:10.1158/0008-5472.CAN-11-0778 Medline

33. M. Weïwer, J. Spoonamore, J. Wei, B. Guichard, N. T. Ross, K. Masson, W. Silkworth, S. Dandapani, M. Palmer, C. A. Scherer, A. M. Stern, S. L. Schreiber, B. Munoz, A potent and selective quinoxalinone-based STK33 inhibitor does not show synthetic lethality in KRAS-dependent cells. *ACS Med. Chem. Lett.* **3**, 1034–1038 (2012). doi:10.1021/ml300246r Medline

34. T. Luo, K. Masson, J. D. Jaffe, W. Silkworth, N. T. Ross, C. A. Scherer, C. Scholl, S. Fröhling, S. A. Carr, A. M. Stern, S. L. Schreiber, T. R. Golub, STK33 kinase inhibitor BRD-8899 has no effect on KRAS-dependent cancer cell viability. *Proc. Natl. Acad. Sci. U.S.A.* **109**, 2860–2865 (2012). doi:10.1073/pnas.1120589109 Medline

35. R. A. Goodnow Jr., C. E. Dumelin, A. D. Keefe, DNA-encoded chemistry: Enabling the deeper sampling of chemical space. *Nat. Rev. Drug Discov.* **16**, 131–147 (2017). doi:10.1038/nrd.2016.213 Medline

36. R. K. Modukuri, D. Monsivais, F. Li, M. Palaniappan, K. M. Bohren, Z. Tan, A. F. Ku, Y. Wang, C. Madasu, J.-Y. Li, S. Tang, G. Miklossy, S. S. Palmer, D. W. Young, M. M. Matzuk, Discovery of highly potent and BMPR2-selective kinase inhibitors using DNA-

encoded chemical library screening. *J. Med. Chem.* **66**, 2143–2160 (2023). doi:10.1021/acs.jmedchem.2c01886 Medline

37. P. A. Harris, S. B. Berger, J. U. Jeong, R. Nagilla, D. Bandyopadhyay, N. Campobasso, C. A. Capriotti, J. A. Cox, L. Dare, X. Dong, P. M. Eidam, J. N. Finger, S. J. Hoffman, J. Kang, V. Kasparcova, B. W. King, R. Lehr, Y. Lan, L. K. Leister, J. D. Lich, T. T. MacDonald, N. A. Miller, M. T. Ouellette, C. S. Pao, A. Rahman, M. A. Reilly, A. R. Rendina, E. J. Rivera, M. C. Schaeffer, C. A. Sehon, R. R. Singhaus, H. H. Sun, B. A. Swift, R. D. Totoritis, A. Vossenkämper, P. Ward, D. D. Wisnoski, D. Zhang, R. W. Marquis, P. J. Gough, J. Bertin, Discovery of a first-in-class receptor interacting protein 1 (RIP1) kinase specific clinical candidate (GSK2982772) for the treatment of inflammatory diseases. *J. Med. Chem.* **60**, 1247–1261 (2017). doi:10.1021/acs.jmedchem.6b01751 Medline

38. P. A. Harris, B. W. King, D. Bandyopadhyay, S. B. Berger, N. Campobasso, C. A. Capriotti, J. A. Cox, L. Dare, X. Dong, J. N. Finger, L. C. Grady, S. J. Hoffman, J. U. Jeong, J. Kang, V. Kasparcova, A. S. Lakdawala, R. Lehr, D. E. McNulty, R. Nagilla, M. T. Ouellette, C. S. Pao, A. R. Rendina, M. C. Schaeffer, J. D. Summerfield, B. A. Swift, R. D. Totoritis, P. Ward, A. Zhang, D. Zhang, R. W. Marquis, J. Bertin, P. J. Gough, DNA-encoded library screening identifies benzo[*b*][1,4]oxazepin-4-ones as highly potent and monoselective receptor interacting protein 1 kinase inhibitors. *J. Med. Chem.* **59**, 2163–2178 (2016). doi:10.1021/acs.jmedchem.5b01898 Medline

39. J. W. M. Nissink, S. Bazzaz, C. Blackett, M. A. Clark, O. Collingwood, J. S. Disch, D. Gikunju, K. Goldberg, J. P. Guilinger, E. Hardaker, E. J. Hennessy, R. Jetson, A. D. Keefe, W. McCoull, L. McMurray, A. Olszewski, R. Overman, A. Pflug, M. Preston, P. B. Rawlins, E. Rivers, M. Schimpl, P. Smith, C. Truman, E. Underwood, J. Warwicker, J. Winter-Holt, S. Woodcock, Y. Zhang, Generating selective leads for mer kinase inhibitors-example of a comprehensive lead-generation strategy. *J. Med. Chem.* **64**, 3165–3184 (2021). doi:10.1021/acs.jmedchem.0c01904 Medline

40. J. D. Vasta, C. R. Corona, J. Wilkinson, C. A. Zimprich, J. R. Hartnett, M. R. Ingold, K. Zimmerman, T. Machleidt, T. A. Kirkland, K. G. Huwiler, R. F. Ohana, M. Slater, P. Otto, M. Cong, C. I. Wells, B.-T. Berger, T. Hanke, C. Glas, K. Ding, D. H. Drewry, K. V. M. Huber, T. M. Willson, S. Knapp, S. Müller, P. L. Meisenheimer, F. Fan, K. V. Wood, M. B. Robers, Quantitative, wide-spectrum kinase profiling in live cells for assessing the effect of cellular ATP on target engagement. *Cell Chem. Biol.* **25**, 206–214.e11 (2018). doi:10.1016/j.chembiol.2017.10.010 Medline

41. M. B. Robers, J. M. Wilkinson, J. D. Vasta, L. M. Berger, B.-T. Berger, S. Knapp, Single tracer-based protocol for broad-spectrum kinase profiling in live cells with NanoBRET. *STAR Protoc.* **2**, 100822 (2021). doi:10.1016/j.xpro.2021.100822 Medline

42. A. C. Pike, P. Rellos, F. H. Niesen, A. Turnbull, A. W. Oliver, S. A. Parker, B. E. Turk, L. H. Pearl, S. Knapp, Activation segment dimerization: A mechanism for kinase autophosphorylation of non-consensus sites. *EMBO J.* **27**, 704–714 (2008). doi:10.1038/emboj.2008.8 Medline

43. F. Kong, T. Sun, X. Kong, D. Xie, Z. Li, K. Xie, Krüppel-like factor 4 suppresses serine/threonine kinase 33 activation and metastasis of gastric cancer through reversing epithelial-mesenchymal transition. *Clin. Cancer Res.* **24**, 2440–2451 (2018). doi:10.1158/1078-0432.CCR-17-3346 Medline

44. X. Li, M. Lin, M. Liu, H. Ye, S. Qin, Interaction between STK33 and autophagy promoted renal cell carcinoma metastasis by regulating mTOR/ULK1 signaling pathway. *Mol. Biol. Rep.* **50**, 5059–5067 (2023). doi:10.1007/s11033-023-08396-3 Medline

45. E. L. Sun, C. X. Liu, Z. X. Ma, X. Y. Mou, X. A. Mu, Y. H. Ni, X. L. Li, D. Zhang, Y. R. Ju, Knockdown of human serine/threonine kinase 33 suppresses human small cell lung carcinoma by blocking RPS6/BAD signaling transduction. *Neoplasma* **64**, 869–879 (2017). doi:10.4149/neo_2017_608 Medline

46. C. Chen, L. Huang, G. Zhang, Y. Li, L. Li, X. Bai, W. Liu, H. Wang, J. Li, STK33 potentiates the malignancy of hypopharyngeal squamous carcinoma: Possible relation to calcium. *Cancer Biol. Ther.* **17**, 976–984 (2016). doi:10.1080/15384047.2016.1210739 Medline

47. S. Zhang, H. Wu, K. Wang, M. Liu, STK33/ERK2 signal pathway contribute the tumorigenesis of colorectal cancer HCT15 cells. *Biosci. Rep.* **39**, BSR20182351 (2019). doi:10.1042/BSR20182351 Medline

48. W. Yu, Y. Li, H. Chen, Y. Cui, C. Situ, L. Yao, X. Zhang, S. Lu, L. Liu, L. Li, J. Ren, Y. Guo, Z. Huo, Y. Chen, H. Li, T. Jiang, Y. Gu, C. Wang, T. Zhu, Y. Li, Z. Hu, X. Guo, STK33 phosphorylates fibrous sheath protein AKAP3/4 to regulate sperm flagella assembly in spermiogenesis. *Mol. Cell. Proteomics* **22**, 100564 (2023). doi:10.1016/j.mcpro.2023.100564 Medline

49. M. Békés, D. R. Langley, C. M. Crews, PROTAC targeted protein degraders: The past is prologue. *Nat. Rev. Drug Discov.* **21**, 181–200 (2022). doi:10.1038/s41573-021-00371-6 Medline

50. S. Röth, N. M. Kocaturk, P. S. Sathyamurthi, B. Carton, M. Watt, T. J. Macartney, K.-H. Chan, A. Isidro-Llobet, A. Konopacka, M. A. Queisser, G. P. Sapkota, Identification of KLHDC2 as an efficient proximity-induced degrader of K-RAS, STK33, β-catenin, and FoxP3. *Cell Chem. Biol.* **30**, 1261–1276.e7 (2023). doi:10.1016/j.chembiol.2023.07.006 Medline

51. J. C. Faver, K. Riehle, D. R. Lancia Jr., J. B. J. Milbank, C. S. Kollmann, N. Simmons, Z. Yu, M. M. Matzuk, Quantitative comparison of enrichment from DNA-encoded chemical library selections. *ACS Comb. Sci.* **21**, 75–82 (2019). doi:10.1021/acscombsci.8b00116 Medline

52. S. Park, J. Fan, S. Chamakuri, M. Palaniappan, K. Sharma, X. Qin, J. Wang, Z. Tan, A. Judge, L. Hu, B. Sankaran, F. Li, B. V. V. Prasad, M. M. Matzuk, T. Palzkill, Exploiting the carboxylate-binding pocket of β-lactamase enzymes using a focused DNA-encoded chemical library. *J. Med. Chem.* **67**, 620–642 (2024). doi:10.1021/acs.jmedchem.3c01834 Medline

53. S. Chamakuri, S. Lu, M. N. Ucisik, K. M. Bohren, Y.-C. Chen, H.-C. Du, J. C. Faver, R. Jimmidi, F. Li, J.-Y. Li, P. Nyshadham, S. S. Palmer, J. Pollet, X. Qin, S. E. Ronca, B. Sankaran, K. L. Sharma, Z. Tan, L. Versteeg, Z. Yu, M. M. Matzuk, T. Palzkill, D. W. Young, DNA-encoded chemistry technology yields expedient access to SARS-CoV-2 M^{pro} inhibitors. *Proc. Natl. Acad. Sci. U.S.A.* **118**, e2111172118 (2021). doi:10.1073/pnas.2111172118 Medline

54. S. Dawadi, N. Simmons, G. Miklossy, K. M. Bohren, J. C. Faver, M. N. Ucisik, P. Nyshadham, Z. Yu, M. M. Matzuk, Discovery of potent thrombin inhibitors from a protease-focused DNA-encoded chemical library. *Proc. Natl. Acad. Sci. U.S.A.* **117**, 16782–16789 (2020). doi:10.1073/pnas.2005447117 Medline

55. D. M. Taylor, J. Anglin, S. Park, M. N. Ucisik, J. C. Faver, N. Simmons, Z. Jin, M. Palaniappan, P. Nyshadham, F. Li, J. Campbell, L. Hu, B. Sankaran, B. V. V. Prasad, H. Huang, M. M. Matzuk, T. Palzkill, Identifying Oxacillinase-48 Carbapenemase inhibitors using DNA-encoded chemical libraries. *ACS Infect. Dis.* **6**, 1214–1227 (2020). doi:10.1021/acsinfecdis.0c00015 Medline

56. O. B. C. Monty, P. Nyshadham, K. M. Bohren, M. Palaniappan, M. M. Matzuk, D. W. Young, N. Simmons, Homogeneous and functional group tolerant ring-closing metathesis for DNA-encoded chemical libraries. *ACS Comb. Sci.* **22**, 80–88 (2020). doi:10.1021/acscombsci.9b00199 Medline

57. J. Y. Li, G. Miklossy, R. K. Modukuri, K. M. Bohren, Z. Yu, M. Palaniappan, J. C. Faver, K. Riehle, M. M. Matzuk, N. Simmons, Palladium-catalyzed hydroxycarbonylation of (hetero)aryl halides for DNA-encoded chemical library synthesis. *Bioconjug. Chem.* **30**, 2209–2215 (2019). doi:10.1021/acs.bioconjchem.9b00447 Medline

58. H. C. Du, N. Simmons, J. C. Faver, Z. Yu, M. Palaniappan, K. Riehle, M. M. Matzuk, A mild, DNA-compatible nitro reduction using $B_2(OH)_4$. *Org. Lett.* **21**, 2194–2199 (2019). doi:10.1021/acs.orglett.9b00497 Medline

59. T. G. Battye, L. Kontogiannis, O. Johnson, H. R. Powell, A. G. Leslie, iMOSFLM: A new graphical interface for diffraction-image processing with MOSFLM. *Acta Crystallogr. D Biol. Crystallogr.* **67**, 271–281 (2011). doi:10.1107/S0907444910048675 Medline

60. P. R. Evans, An introduction to data reduction: Space-group determination, scaling and intensity statistics. *Acta Crystallogr. D Biol. Crystallogr.* **67**, 282–292 (2011). doi:10.1107/S090744491003982X Medline

61. A. J. McCoy, R. W. Grosse-Kunstleve, P. D. Adams, M. D. Winn, L. C. Storoni, R. J. Read, Phaser crystallographic software. *J. Appl. Crystallogr.* **40**, 658–674 (2007). doi:10.1107/S0021889807021206 Medline

62. J. Jumper, R. Evans, A. Pritzel, T. Green, M. Figurnov, O. Ronneberger, K. Tunyasuvunakool, R. Bates, A. Žídek, A. Potapenko, A. Bridgland, C. Meyer, S. A. A. Kohl, A. J. Ballard, A. Cowie, B. Romera-Paredes, S. Nikolov, R. Jain, J. Adler, T. Back, S. Petersen, D. Reiman, E. Clancy, M. Zielinski, M. Steinegger, M. Pacholska, T. Berghammer, S. Bodenstein, D. Silver, O. Vinyals, A. W. Senior, K. Kavukcuoglu, P. Kohli, D. Hassabis, Highly accurate protein structure prediction with AlphaFold. *Nature* **596**, 583–589 (2021). doi:10.1038/s41586-021-03819-2 Medline

63. P. V. Afonine, R. W. Grosse-Kunstleve, N. Echols, J. J. Headd, N. W. Moriarty, M. Mustyakimov, T. C. Terwilliger, A. Urzhumtsev, P. H. Zwart, P. D. Adams, Towards automated crystallographic structure refinement with phenix.refine. *Acta Crystallogr. D Biol. Crystallogr.* **68**, 352–367 (2012). doi:10.1107/S0907444912001308 Medline

64. P. Emsley, K. Cowtan, Coot: Model-building tools for molecular graphics. *Acta Crystallogr. D Biol. Crystallogr.* **60**, 2126–2132 (2004). doi:10.1107/S0907444904019158 Medline

65. W. DeLano, *The PyMOL molecular graphics system* (DeLano Scientific, 2002).

66. Schrödinger Release 2022-1: Schrödinger, LLC, New York, NY (2022).

67. S. Release, *2022-1: LigPrep* (Schrödinger, LLC, 2022).

68. R. C. Johnston, K. Yao, Z. Kaplan, M. Chelliah, K. Leswing, S. Seekins, S. Watts, D. Calkins, J. Chief Elk, S. V. Jerome, M. P. Repasky, J. C. Shelley, Epik: pKa and protonation state prediction through machine learning. *J. Chem. Theory Comput.* **19**, 2380–2388 (2023). doi:10.1021/acs.jctc.3c00044 Medline

69. R. A. Friesner, R. B. Murphy, M. P. Repasky, L. L. Frye, J. R. Greenwood, T. A. Halgren, P. C. Sanschagrin, D. T. Mainz, Extra precision glide: Docking and scoring incorporating a model of hydrophobic enclosure for protein-ligand complexes. *J. Med. Chem.* **49**, 6177–6196 (2006). doi:10.1021/jm051256o Medline

70. S. Release, *2023-4: Maestro* (Schrödinger, LLC, 2023).

71. P. Kuzmič, DynaFit—A software package for enzymology. *Methods Enzymol.* **467**, 247–280 (2009). doi:10.1016/S0076-6879(09)67010-5 Medline

72. X. Qin, C. Xie, J. M. Hakenjos, K. R. MacKenzie, S. R. Boyd, M. Barzi, K.-D. Bissig, D. W. Young, F. Li, The roles of Cyp1a2 and Cyp2d in pharmacokinetic profiles of serotonin and norepinephrine reuptake inhibitor duloxetine and its metabolites in mice. *Eur. J. Pharm. Sci.* **181**, 106358 (2023). doi:10.1016/j.ejps.2022.106358 Medline

73. K. Kent, K. Nozawa, C. Sutton, F. Daniel, M. Ikawa, T. X. Garcia, M. M. Matzuk, CUB domains are not required for OVCH2 function in sperm maturation in the mouse epididymis. *Andrology* **12**, 682–697 (2024). doi:10.1111/andr.13508 Medline

74. J. Pei, N. V. Grishin, PROMALS3D: Multiple protein sequence alignment enhanced with evolutionary and three-dimensional structural information. *Methods Mol. Biol.* **1079**, 263–271 (2014). doi:10.1007/978-1-62703-646-7_17 Medline

75. J. D. Thompson, D. G. Higgins, T. J. Gibson, CLUSTAL W: Improving the sensitivity of progressive multiple sequence alignment through sequence weighting, position-specific gap penalties and weight matrix choice. *Nucleic Acids Res.* **22**, 4673–4680 (1994). doi:10.1093/nar/22.22.4673 Medline

76. X. Robert, P. Gouet, Deciphering key features in protein structures with the new ENDscript server. *Nucleic Acids Res.* **42**, W320–W324 (2014). doi:10.1093/nar/gku316 Medline
