# Supplementary material for: Reversible male contraception by targeted inhibition of serine/threonine kinase 33
Source: Science. Author manuscript; Available in PMC 2025 Feb 20. (PMC11842024; doi:10.1126/science.adl2688)
Supplement: MDAR Reproducibility Checklist [file NIHMS2052210-supplement-MDAR_Reproducibility_Checklist.pdf]

Materials Design Analysis Reporting (MDAR) Checklist for Authors

The MDAR framework establishes a minimum set of requirements in transparent reporting applicable to studies in the life sciences (see Statement of Task: doi:10.31222/osf.io/9sm4x.). The MDAR checklist is a tool for authors, editors, and others seeking to adopt the MDAR framework for transparent reporting in manuscripts and other outputs. Please refer to the MDAR Elaboration Document for additional context for the MDAR framework.

For all that apply, please note where in the manuscript the required information is provided.

Materials:

Newly created materials	indicate where provided: page no/section/legend)	n/a
The manuscript includes a dedicated "materials availability statement" providing transparent disclosure about availability of newly created materials including details on how materials can be accessed and describing any restrictions on access.	Access to our in-house DNA-encoded libraries (DELs) can be achieved through open collaborative relationship; Supplemental Material: Page 2, "DEC-Tec affinity selections" section.<br>Assayed compounds are available by following the synthetic protocol or collaborative relationship; Supplemental Material: Page 8, "Experimental procedures and characterization data of the synthesized compounds" section.<br>The crystal structure (PDB ID: 8VF6) can be accessed after one year of hold from https://www.rcsb.org/; Supplemental Material: Page 45, "Table S1. Data collection and refinement statistics".	

Antibodies	indicate where provided: page no/section/legend)	n/a
For commercial reagents, provide supplier name, catalogue number and RRID, if available.		n/a

DNA and RNA sequences	indicate where provided: page no/section/legend)	n/a
**Short novel DNA or RNA including primers, probes:** Sequences should be included or deposited in a public repository.		n/a

Cell materials	indicate where provided: page no/section/legend	n/a
**Cell lines:** Provide species information, strain. Provide accession number in repository **OR** supplier name, catalog number, clone number, **OR** RRID.	SF9 cells (ThermoFisher Scientific, Catalog #: 12659017); Supplemental Material: Page 2, "Crystallization, data collection, and structure solution" section.<br>HEK293T cells (Human, fetus, ATCC 293T CRL-3216); Supplemental Material: Page 3, "NanoBRET Target Engagement intracellular kinase assay" section.<br>HEK293 cells (Human, fetus, ATCC CRL-1573) used by Promega; Supplemental Material: Page 4, "NanoBRET Target Engagement K192 assay" section.	
**Primary cultures:** Provide species, strain, sex of origin, genetic modification status.		n/a

Experimental animals	indicate where provided: page no/section/legend)	n/a
**Laboratory animals or Model organisms:** Provide species, strain, sex, age, genetic modification status. Provide accession number in repository **OR** supplier name, catalog number, clone number, **OR** RRID.	C57BL/6NJ male mice, 8–12-week-old; Supplemental Material: Page 4, "Pharmacokinetics (PK) of CDD-2807 in mice" section.<br>C57/129 hybrid male mice minimum of 8 weeks old during testing; Supplemental Material: Page 6, "Fertility analysis" section.	
**Animal observed in or captured from the field:** Provide species, sex, and age where possible.		n/a

Plants and microbes	indicate where provided: page no/section/legend)	n/a
**Plants:** provide species and strain, ecotype and cultivar where relevant, unique accession number if available, and source (including location for collected wild specimens).		n/a
**Microbes:** provide species and strain, unique accession number if available, and source.		n/a

Human research participants	indicate where provided: page no/section/legend) or state if these demographics were not collected	n/a
If collected and within the bounds of privacy constraints report on age, sex and gender or ethnicity for all study participants.		n/a

Design:

Study protocol	indicate where provided: page no/section/legend)	n/a
If study protocol has been pre-registered, provide DOI. For clinical trials, provide the trial registration number **OR** cite DOI.		n/a

Laboratory protocol	indicate where provided: page no/section/legend)	n/a
Provide DOI **OR** other citation details if detailed step-by-step protocols are available.	All procedures have been described in the Supplemental Material or described in previous references.	

Experimental study design (statistics details)		
**For in vivo studies:** State whether and how the following have been done	**indicate where provided: page no/section/legend. If it could have been done, but was not, write not done**	**n/a**
Sample size determination	Three mice in each group were used in the pharmacokinetics study, and no statistics analysis is needed; Supplemental Material: Page 4, "Pharmacokinetics (PK) of CDD-2807 in mice" section. Numbers of mice in the fertility assessment (and recovery) were selected to be able to accommodate the wide variety of experiments that were needed to complete all aspects of the research. An n of 6 was used in protocol 1 but given the dramatic effect on the breeding of mice when we began our collections, so we decided to increase the n to 7 in protocol 2 to ensure that we still had a minimum of 3 mice to undergo the recovery portion of the study; Fig. 4 (and legend) and Supplemental Material: Pages 39 and 41–42, "fig. S21. Protocol timelines of mouse breeding and reversibility study" legend and fig. S23 (and legend).	
Randomisation	Mice in the fertility assessment (and recovery) were randomly assigned to either control or CDD-2807 treatment groups; Fig. 4 (and legend) and Supplemental Material: Pages 39 and 41–42, "fig. S21. Protocol timelines of mouse breeding and reversibility study" legend and fig. S23 (and legend).	
Blinding		n/a
Inclusion/exclusion criteria		n/a

Sample definition and in-laboratory replication	indicate where provided: page no/section/legend	n/a
State number of times the experiment was replicated in laboratory.	For CDD-2807, metabolic stability assays in liver microsomes were performed twice for CDD-2807 and once for other compounds; Supplemental Material: Page 4, "Metabolic stability assay in liver microsomes" section.<br>The animal experiments for fertility analysis were conducted three times; one at a lower drug concentration for 45 days, and two at higher drug concentrations (one for 7 days and one for 63 days); Fig. 4 (and legend) and Supplemental Material: Pages 39 and 41–42, "fig. S21. Protocol timelines of mouse breeding and reversibility study" legend and fig. S23 (and legend).	
Define whether data describe technical or biological replicates.	Technical replicates were used for in vitro biochemical assays; Supplemental Material: Page 3, "In vitro biochemical assays" section.<br>Biological replicates were used for metabolic stability assays; Supplemental Material: Page 4, "Metabolic stability assay in liver microsomes" section.<br>A minimum of 3 biological replicates for each group	

	were used in the animal experiments for fertility analysis; Fig. 4 (and legend) and Supplemental Material: Pages 39 and 41–42, "fig. S21. Protocol timelines of mouse breeding and reversibility study" legend and fig. S23 (and legend).	

Ethics	indicate where provided: page no/section/legend	n/a
**Studies involving human participants:** State details of authority granting ethics approval (IRB or equivalent committee(s), provide reference number for approval.		n/a
**Studies involving experimental animals:** State details of authority granting ethics approval (IRB or equivalent committee(s), provide reference number for approval.	Animal procedures have been approved by the IACUC committee at Baylor College of medicine under protocol number AN-716; Supplemental Material: Page 6, "Fertility analysis" section.	
**Studies involving specimen and field samples:** State if relevant permits obtained, provide details of authority approving study; if none were required, explain why.		n/a

Dual Use Research of Concern (DURC)	indicate where provided: page no/section/legend	n/a
If study is subject to dual use research of concern regulations, state the authority granting approval and reference number for the regulatory approval.		n/a

Analysis:

Attrition	indicate where provided: page no/section/legend	n/a
Describe whether exclusion criteria were preestablished. Report if sample or data points were omitted from analysis. If yes report if this was due to attrition or intentional exclusion and provide justification.		n/a

Statistics	indicate where provided: page no/section/legend	n/a
Describe statistical tests used and justify choice of tests.	For in vitro biochemical assays, values of parameters (K_i and K_d) are expressed as mean ± standard errors; Figure 2B and Supplemental Material: Page 3, "In vitro biochemical assays" section.<br>For all statistics done on the animal studies, all data was tested for normality using the Shapiro-Wilk normality test. All data that had normal distribution were then analyzed using a t-test. Significance was denoted as any value ≥ 0.05; Supplemental Material: Page 6, "Fertility analysis" section.	

Data availability	indicate where provided: page no/section/legend	n/a
For newly created and reused datasets, the manuscript includes a data availability statement that provides details for access or notes restrictions on access.	There are no restrictions on LC-MS data for metabolic stability, pharmacokinetics, and tissue distribution. The raw data of LC-MS data can be accessed by contacting the corresponding author; Supplemental Material: Pages 4–6, "Metabolic stability assay in liver microsomes", "Pharmacokinetics (PK) of CDD-2807 in mice", and "CDD-2807 concentrations in mouse brain, liver, lung, and testis" sections.<br>Biochemical assays: datasets from ThermoFisher are available on demand by contacting the corresponding author; Supplemental Material: Page 3, "In vitro biochemical assays" section.	
If newly created datasets are publicly available, provide accession number in repository **OR** DOI **OR** URL and licensing details where available.	Structure coordinates and structure factors are deposited in https://www.rcsb.org/ as PDB ID: 8VF6. Material can be accessed after one year of hold; Supplemental Material: Page 45, "Table S1. Data collection and refinement statistics".	
If reused data is publicly available provide accession number in repository **OR** DOI **OR** URL, **OR** citation.		n/a

Code availability	indicate where provided: page no/section/legend	n/a
For all newly generated custom computer code/software/mathematical algorithm or re-used code essential for replicating the main findings of the study, the manuscript includes a data availability statement that provides details for access or notes restrictions.		n/a
If newly generated code is publicly available, provide accession number in repository, **OR** DOI **OR** URL and licensing details where available. State any restrictions on code availability or accessibility.		n/a
If reused code is publicly available provide accession number in repository **OR** DOI **OR** URL, **OR** citation.	iMosflm (version 7.4.0), Phenix (version 1.21rc1-5058), Coot (version 0.9.8.7 EL), and PyMOL (version 2.2.0 Open-Source) are freely available to all academic researchers (references 59, 63, 64, and 65); Supplemental Material: Page 2, "Crystallization, data collection, and structure solution" section.<br>Schrödinger software suite is freely available to all	

	academic researchers (references 66, 67, and 70); Supplemental Material: Page 2, “Computational modeling of STK33/CDD-2807 complex” section.<br>DynaFit software is freely available to all academic researchers (reference 71); Supplemental Material: Page 3, “Calculation of K_d values for kinase inhibitors that bind to the ATP pocket of a kinase from LanthaScreen binding data” section.<br>GraphPad Prism software is commercially available; Supplemental Material: Page 3, “Calculation of K_i values for kinase inhibitors from Z’-LYTE inhibition data” section.	

Reporting

MDAR framework recommends adoption of discipline-specific guidelines, established and endorsed through community initiatives. Journals have their own policy about requiring specific guidelines and recommendations to complement MDAR.

Adherence to community standards	indicate where provided: page no/section/legend	n/a
State if relevant guidelines (e.g., ICMJE, MIBBI, ARRIVE) have been followed, and whether a checklist (e.g., CONSORT, PRISMA, ARRIVE) is provided with the manuscript.		n/a
